
JOURNAL INFORMATION
==============================
NLM Title Abbreviation: Insights Imaging
Journal ID: Insights Imaging
NLM Journal ID: 101532453

Insights into Imaging

EISSN: 1869-4101
Publisher: Springer

ARTICLE INFORMATION
==============================
PMCID: PMC12222569
PMID: 40603764
DOI: 10.1186/s13244-025-02017-2
Article ID: 2017
Article version: 1
Subjects: Meeting Abstracts

ESGAR 2025 Book of Abstracts

Electronic publication date: 2025 Jul 2
Publication date: 2025 Dec
Volume: 16
Issue: Suppl 2
Issue sponsor: Publication costs were funded by the European Society of Gastrointestinal and Abdominal Radiology.
Electronic Location ID: 148
Copyright: © The Author(s) 2025
Copyright year: 2025
License: Open Access This article is licensed under a Creative Commons Attribution 4.0 International License, which permits use, sharing, adaptation, distribution and reproduction in any medium or format, as long as you give appropriate credit to the original author(s) and the source, provide a link to the Creative Commons licence, and indicate if changes were made. The images or other third party material in this article are included in the article’s Creative Commons licence, unless indicated otherwise in a credit line to the material. If material is not included in the article’s Creative Commons licence and your intended use is not permitted by statutory regulation or exceeds the permitted use, you will need to obtain permission directly from the copyright holder. To view a copy of this licence, visit http://creativecommons.org/licenses/by/4.0/.
License URL: https://creativecommons.org/licenses/by/4.0/

Conference name: ESGAR 2025 Annual Meeting
Conference location: Amsterdam, The Netherlands
Conference date: 13-16 May 2025
issue-copyright-statement: © European Society of Radiology (ESR) 2025

==============================

EUROPEAN SOCIETY OF RADIOLOGY

European Society ESGAR Gastrointestinal and Abdominal Radiology

### Insights into Imaging

ESGAR 2025 Book of Abstracts
Volume 16 / Supplement 2
June 2025

Springer

ESGAR 2025 / May 13 – 16, 2025 / Amsterdam, The Netherlands
36th Annual Meeting and Postgraduate Course

European Society
ESGAR
Gastrointestinal and Abdominal Radiology

### ESGAR

### 36th Annual Meeting and Postgraduate Course

### 2025

### May 13-16

AMSTERDAM
THE NETHERLANDS

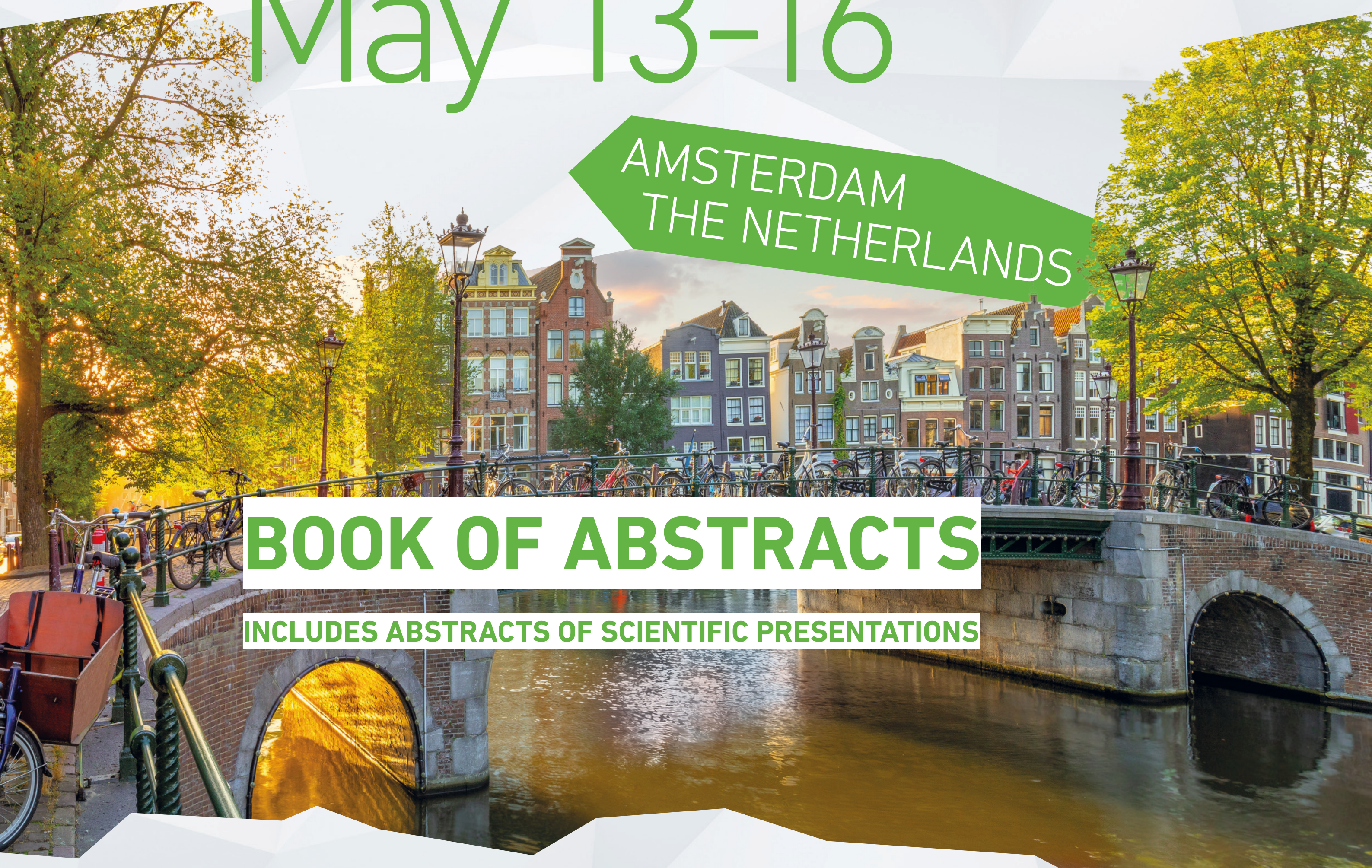

### BOOK OF ABSTRACTS

**INCLUDES ABSTRACTS OF SCIENTIFIC PRESENTATIONS**

*IMPORTANT ADDRESSES*

**ORGANISING SECRETARIAT**
**Central ESGAR Office**
Esslinggasse 2/3
AT – 1010 Vienna
Phone: +43 1 535 89 27
E-Mail: office@esgar.org www.esgar.org

### CME CREDITS

The "ESGAR European Society of Gastrointestinal and Abdominal Radiology" (or) "ESGAR 2025 – 36th ANNUAL MEETING AND POSTGRADUATE COURSE" is accredited by the European Accreditation Council for Continuing Medical Education (EACCME) to provide the following CME activity for medical specialists. The EACCME is an institution of the European Union of Medical Specialists (UEMS), www.uems.net.

The 36th Annual Meeting and Postgraduate Course ESGAR 2025, Amsterdam, Netherlands, 13/05/2025 – 16/05/2025 has been accredited by the European Accreditation Council for Continuing Medical Education (EACCME®) with 29 European CME credits (ECMEC®s). Each medical specialist should claim only those hours of credit that he/she actually spent in the educational activity.

### CONTRIBUTING SOCIETIES

### SPONSORS

ESGAR wishes to gratefully acknowledge the support of its Corporate Members and Sponsors:

The Final Programme of ESGAR 2025 is available on the ESGAR Website www.esgar.org

Date of publishing: May 2025

### TABLE OF CONTENTS

| | | |
|---|---|---|
| Scientific Sessions, Wednesday, May 14 | (SS 1.1 – SS 1.9) | 8-10 |
| Scientific Sessions, Wednesday, May 14 | (SS 2.1 – SS 2.10) | 10-13 |
| Scientific Sessions, Wednesday, May 14 | (SS 15.1 – SS 15.5) | 13-14 |
| Scientific Sessions, Wednesday, May 14 | (SS 3.1 – SS 3.10) | 15-16 |
| Scientific Sessions, Wednesday, May 14 | (SS 4.1 – SS 4.10) | 17-19 |
| Scientific Sessions, Wednesday, May 14 | (SS 5.1 – SS 5.10) | 19-21 |
| Scientific Sessions, Thursday, May 15 | (SS 6.1 – SS 6.10) | 22-24 |
| Scientific Sessions, Thursday, May 15 | (SS 7.1 – SS 7.10) | 24-26 |
| Scientific Sessions, Thursday, May 15 | (SS 16.1 – SS 16.5) | 27-28 |
| Scientific Sessions, Thursday, May 15 | (SS 8.1 – SS 8.10) | 28-30 |
| Scientific Sessions, Thursday, May 15 | (SS 9.1 – SS 9.10) | 31-33 |
| Scientific Sessions, Thursday, May 15 | (SS 10.1 – SS 10.10) | 34-36 |
| Scientific Sessions, Friday, May 16 | (SS 11.1 – SS 11.9) | 37-39 |
| Scientific Sessions, Friday, May 16 | (SS 12.1 – SS 12.9) | 39-41 |
| Scientific Sessions, Friday, May 16 | (SS 17.1 – SS 17.5) | 41-42 |
| Scientific Sessions, Friday, May 16 | (SS 13.1 – SS 13.10) | 43-45 |
| Scientific Sessions, Friday, May 16 | (SS 14.1 – SS 14.10) | 45-47 |
| AUTHORS' INDEX | | 49-54 |

European Society Gastrointestinal and Abdominal Radiology ESGAR

Insights Imaging (2025) Vol 16 (Suppl 2)
https://doi.org/10.1186/s13244-025-02017-2

### ESGAR EXECUTIVE COMMITTEE

PRESIDENT
*M. Zins, Paris/FR*

PRESIDENT-ELECT
*J. Stoker, Amsterdam/NL*

VICE PRESIDENT
*G. Brancatelli, Palermo/IT*

SECRETARY/TREASURER
*V. Vilgrain, Clichy/FR*

PROGRAMME COMMITTEE CHAIR
*J. Stoker, Amsterdam/NL*

EDUCATION COMMITTEE CHAIR
*C.J. Zech, Basel/CH*

MEMBERSHIP COMMITTEE CHAIR
*D. Tolan, Leeds/UK*

RESEARCH COMMITTEE CHAIR
*S.A. Taylor, London/UK*

WORKSHOP COMMITTEE CHAIR
*S. Gourtsoyianni, Athens/GR*

MEETING PRESIDENT 2025
*J. Stoker, Amsterdam/NL*

MEETING PRESIDENT 2026
*I. Millet, Montpellier/FR*

MEMBERS AT LARGE
*M.A. Bali, Rome/IT*
*M. Karcaaltincaba, Ankara/TR*
*N. Kartalis, Stockholm/SE*
*M. Radzina, Riga/LV*

ESGAR EXECUTIVE DIRECTOR
*B. Lindlbauer, Vienna/AT*

### ESGAR 2025 MEETING PRESIDENT

*Prof. Jaap Stoker*
*Dept. of Radiology and Nuclear Medicine*
*Amsterdam University Medical Center*
*The Netherlands*

### ESGAR 2025 PROGRAMME COMMITTEE

CHAIRMEN
*G. Brancatelli, Palermo/IT*
*J. Stoker, Amsterdam/NL*

MEMBERS
*M.A. Bali, Rome/IT*
*S. Gourtsoyianni, Athens/GR*
*M. Karcaaltincaba, Ankara/TR*
*N. Kartalis, Stockholm/SE*
*I. Millet, Montpellier/FR*
*M. Radzina, Riga/LV*
*S.A. Taylor, London/UK*
*D. Tolan, Leeds/UK*
*V. Vilgrain, Clichy/FR*
*C.J. Zech, Basel/CH*
*M. Zins, Paris/FR*

Young ESGAR
*N. Bogveradze, Vienna/AT*
*R. Cannella, Palermo/IT*
*F. Castagnoli, London/UK*
*K. De Paepe, Boston, MA/US*

### ESGAR 2025 DUTCH SUPPORTING GROUP

*R.G.H. Beets-Tan, Amsterdam/NL*
*K. Horsthuis, Amsterdam/NL*
*D. Lambregts, Amsterdam/NL*
*B. Martens, Maastricht/NL*
*C. Mihl, Maastricht /NL*
*J. Nederend, Eindhoven/NL*
*L. Oudenhoven, Almelo/NL*
*J.P. Pennings, Groningen/NL*
*J.B.C.M. Puylaert, Amsterdam/NL*
*J. Tielbeek, Haarlem/NL*
*I. Verpalen, Amsterdam/NL*
*F. Wessels, Utrecht/NL*

*O. Akhan, Ankara/TR*
*M.A. Bali, Rome/IT*
*T.V. Bartolotta, Palermo/IT*
*A. Ba-Ssalamah, Vienna/AT*
*N. Bastati-Huber, Vienna/AT*
*R.G.H. Beets-Tan, Amsterdam/NL*
*E. Biscaldi, Genoa/IT*
*A. Blachar, Tel Aviv/IL*
*L. Blomqvist, Stockholm/SE*
*N. Bogveradze, Vienna/IT*
*I. Boulay Coletta, Paris/FR*
*G. Brancatelli, Palermo/IT*
*D. Breen, Southampton/UK*
*R. Cannella, Palermo/IT*
*F. Caseiro Alves, Coimbra/PT*
*F. Castagnoli, London/UK*
*L. Curvo-Semedo, Coimbra/PT*
*G. d'Assignies, La Roche-sur-Yon/FR*
*M. D'Onofrio, Verona/IT*
*I. De Kock, Ghent/BE*
*K. De Paepe, Boston, MA/US*
*M. Dioguardi Burgio, Clichy/FR*
*A. Dohan, Paris/FR*
*R.F. Dondelinger, Liège/BE*
*A. Furlan, Pittsburgh, PA/US*
*M. Galia, Palermo/IT*
*V. Goh, London/UK*
*M. Gollub, New York, NY/US*
*T. Helmberger, Munich/DE*
*K. Horsthuis, Amsterdam/NL*
*S. Jackson, Plymouth/UK*
*M. Karçaaltincaba, Ankara/TR*
*N. Kartalis, Stockholm/SE*
*A. Laghi, Rome/IT*
*M. Lahaye, Amsterdam/NL*
*D. Lambregts, Amsterdam/NL*
*J.M. Lee, Seoul/KR*
*G.F. Lo Re, Palermo/IT*
*O. Lucidarme, Paris/FR*
*M. Maas, Amsterdam/NL*
*A.J. Madureira, Porto/PT*
*T. Mang, Vienna/AT*
*V. Maniatis, Sønderborg/DK*
*L. Martí-Bonmatí, Valencia/ES*
*C. Matos, Lisbon/PT*
*Y. Menu, Paris/FR*
*C. Mihl, Maastricht/NL*
*G. Morana, Treviso/IT*
*S. Mulé, Créteil/FR*
*J. Nederend, Eindhoven/NL*
*V. Obmann, Bern/CH*
*P. Paolantonio, Rome/IT*
*N. Papanikolaou, Lisbon/PT*
*A. Perez Girbes, Valencia/ES*
*A. Plumb, Esher/UK*
*S. Pötter-Lang, Vienna/AT*
*R. Pozzi Mucelli, Huddinge/SE*
*P. Prassopoulos, Thessaloniki/GR*
*M. Radzina, Riga/LV*
*C. Reiner, Zurich/CH*
*P. Rodríguez Carnero, Madrid/ES*
*M. Ronot, Clichy/FR*
*W. Schima, Vienna/AT*
*S. Schmidt Kobbe, Lausanne/CH*
*A. Schreyer, Brandenburg an der Havel/DE*
*E. Soloff, Seattle, WA/US*
*S. Stojanovic, Novi Sad/RS*
*J. Stoker, Amsterdam/NL*
*B. Sureka, Jodhpur/IN*
*A. Taibbi, Palermo/IT*
*B. Taouli, New York, NY/US*
*J.P. Tasu, Poitiers/FR*
*S.A. Taylor, London/UK*
*J. Tielbeek, Heemstede/NL*
*D. Tolan, Leeds/UK*
*C. Triantopoulou, Athens/GR*
*J. Van Griethuysen, Amsterdam/NL*
*F. Vernuccio, Palermo/IT*
*V. Vilgrain, Clichy/FR*
*M.-P. Vullierme, Epagny Metz-Tessy/FR*
*M. Wagner, Paris/FR*
*G. Zamboni, Verona/IT*
*C.J. Zech, Basel/CH*
*M. Zins, Paris/FR*

09:00 – 10:30 E 103/104

*Scientific Session SS 1*

### HCC: detection, classification and grading

### SS 1.1

### Artificial intelligence-powered automated detection of hepatocellular carcinoma on multiphasic contrast-enhanced MRI

M. Iskandar[1], P. Roca[2], B. Lambert[2], H. Dehaene[2], G. Jarre[2], S. Doyle[2], V. Vilgrain[1]; [1]*Clichy/FR*, [2]*Grenoble/FR*

**Purpose:** Non-invasive imaging, such as multiphasic contrast-enhanced MRI, is essential for the early diagnosis of hepatocellular carcinoma (HCC). Liver Imaging Reporting and Data System (LI-RADS) is used as a reading and standardization tool (specificity >95% of LI-RADS 5 in HCC diagnosis). However, the diagnosis of small HCC remains challenging, with a reported radiologist sensitivity of 71–73% (10–20 mm and 20–30 mm size, respectively). In this study, we propose an artificial intelligence (AI)-powered automated tool to assist radiologists in detecting LR5 observations in patients at risk for HCC.

**Material and Methods:** Data were taken from the CHIC multicentric study (prospective enrollment of patients at risk for HCC) and consisted of multiphasic contrast-enhanced MRI of cirrhotic patients presenting nodules of 10 to 30 mm. The segmentation of supracentimetric observations was performed by an expert radiologist using AI DReAM platform (GE HealthCare). After the automatic registration of delayed and portal phases on the arterial phase, a 3D U-Net was trained to segment LR5 observations using the three phases as input.

**Results:** The model was trained on 224 patients (80%, 197 LR5 observations) and evaluated on a separate validation dataset of 59 patients (20%, 54 LR5 observations). It achieved a lesion-level sensitivity of 81% (CI: 72–88) for detecting LR5 observations, with a mean of 1.9 false positives per scan. The sensitivity for automatically detecting LR5 observations was 77% (CI: 67–88) for 10–20 mm and 88% (CI: 77–100) for 20–30 mm.

**Conclusion:** With 81% sensitivity for detecting LR5 observations under 30 mm in patients at risk for HCC, the proposed model is a promising tool to assist radiologists in the diagnosis of small HCC.

### SS 1.2

### The value of Liver Imaging Reporting and Data System v2018 ancillary features for hepatocellular carcinoma ≤ 30 mm in Western and Eastern guidelines on both extracellular agent and gadoxetic acid-enhanced MRI

J. Zhou, D. Zhang, H. Chen, Y. Xiao, S. Ouyang, Y. Zeng, Z. Fang, J. Wang; *Guangzhou/CN*

**Purpose:** To evaluate the value of the Liver Imaging Reporting and Data System (LI-RADS) v2018 ancillary features (AFs) for HCCs ≤ 30 mm on extracellular contrast agent-enhanced MRI (ECA-MRI) and gadoxetate disodium-enhanced MRI (EOB-MRI).

**Material and Methods:** This retrospective study included patients at risk for HCC who underwent MRI from 2016 to 2022. The odds ratios of LI-RADS features and high alpha-fetoprotein (AFP) levels were assessed. Western and Eastern guidelines' diagnostic performances [LI-RADS v2018, European Association for the Study of the Liver (EASL) v2018, Korean Liver Cancer Association-National Cancer Center (KLCA-NCC) v2022, Asian Pacific Association for the Study of the Liver (APASL) v2017] were compared via generalized estimating equations.

**Results:** The ECA-MRI training/validation sets included 573 and 245 observations of HCCs ≤ 30 mm, respectively, in 641 patients, and the EOB-MRI validation set included 618 observations in 579 patients. Mild-moderate T2 hyperintensity plus fat in mass (or AFP ≥ 200 ng/mL), named "T2+F(A)", was driven from the features independently associated with HCC. Using the T2+F(A) criterion significantly improved the sensitivities of LI-RADS v2018 (80.7–85.0% vs. 73.1–77.1%, P < 0.001), EASL v2018 (82.0–84.3% vs. 73.4–75.7%, P < 0.001), KLCA-NCC v2022 (81.2–90.9% vs. 73.4–88.3%, P < 0.001) and APASL v2017 (82.6–94.8% vs. 73.7–90.9%, P < 0.001) for HCCs ≤ 30 mm in the training and validation sets without impairing specificity, except that of APASL v2017 in the EOB validation set (75.5% vs. 77.2%, P = 0.024).

**Conclusion:** The use of LI-RADS AFs and high AFP level can improve the sensitivity of Western and Eastern guidelines for diagnosing HCCs ≤ 30 mm.

### SS 1.3

### Value of threshold growth for the diagnosis of hepatocellular carcinoma using the Liver Imaging Reporting and Data System

J. Bae, J. Lee, J. Kim, S. Jeon, J. Yoo; *Seoul/KR*

**Purpose:** The utility of threshold growth (TG) in hepatocellular carcinoma (HCC) imaging remains contentious across major guidelines. This study aimed to investigate the diagnostic implications of TG in HCC diagnosis using the criteria set by Liver Imaging Reporting and Data System (LI-RADS).

**Material and Methods:** In this single-center retrospective study, three radiologists independently evaluated pre-transplantation hepatobiliary agent-enhanced MR images and prior CT/MR images using LI-RADS v2018 in consecutive patients who underwent liver transplantation between January 2010 and November 2022. TG was defined as a ≥50% size increase in ≤6 months. Explanted livers served as reference standards. Frequencies of TG between HCCs and non-HCCs were compared using Fisher's exact test, and interobserver agreement was assessed using Fleiss' κ statistics. The diagnostic performance of LI-RADS category 5 in the diagnosis of HCC was assessed with and without considering TG as a major feature. McNemar's tests were used to compare results.

**Results:** The cohort included 158 patients (mean age, 59.1 ± 7.5 years; 130 males) with 280 observations (207 HCCs, 5 non-HCC malignancies, and 68 benign lesions). TG was identified in 44 (15.7%) observations. Interobserver agreement on TG was moderate (κ = 0.280). Incorporating TG as a major feature significantly enhanced the sensitivity of LI-RADS category 5 in diagnosing HCC (33.8% vs. 40.6%, p < 0.001) without compromising specificity (100.0% vs. 94.5%, p = 0.125).

**Conclusion:** Incorporating TG as a major criterion in LI-RADS category 5 enhanced the diagnostic sensitivity for HCC in liver transplant candidates with minimal impact on specificity.

### SS 1.4

### Abbreviated MRI protocol for early detection of primary liver malignancies in patients with thalassemia

I. Sgantzou[1], T. Floros[1], C. Gouta[1], A. Bara[1], S. Gourtsoyianni[2], E. Alexiou[1]; [1]*Larissa/GR*, [2]*Athens/GR*

**Purpose:** Thalassemia, a hereditary blood disorder characterized by irregular hemoglobin synthesis, has been associated with an increased risk of developing hepatocellular carcinoma, mainly due to iron overload and hepatitis virus infection. This study emphasizes the importance of an abbreviated MRI protocol during imaging follow-up of this patient group for iron overload, for the early detection of liver malignancies.

**Material and Methods:** An abbreviated MRI protocol, comprising axial DWI (b: 50, 400, 800), axial high resolution T2 TSE and GRE/T1 in-opposed phase sequences, for primary liver tumor detection has been incorporated into the standard-of-care MRI performed in a Siemens Magnetom Avanto 1.5T scanner for iron quantification in patients with thalassemia. An IRB approval has been obtained for this study. A retrospective review of studies was performed in our department from 2017 to 2023. Patients with positive findings at the time were recalled for iv contrast administration, and lesion characterization based on LI-RADS criteria was performed.

**Results:** 317 patients have been monitored with the combined MRI protocol. 7 patients were found to have an HCC, T1a/b stage (n = 6) and T2 stage (n = 1). In addition, in 3 patients an intrahepatic cholangiocarcinoma was identified: T1a stage (n = 2) and T2 stage (n = 1). None of these patients had pathological findings in their recent abdominal ultrasound, which is routinely performed for screening.

**Conclusion:** We propose this abbreviated MRI protocol as a life-saving tool for the early detection of liver malignancies in this high-risk patient group.

### SS 1.5

### CT and MRI imaging features of hepatocellular carcinoma in patients with thalassemia

T. Floros[1], I. Sgantzou[1], F. Psoma[1], A. Ninika[1], S. Gourtsoyianni[2], E. Alexiou[1]; *[1]Larissa/GR, [2]Athens/GR*

**Purpose:** Hepatocellular carcinoma (HCC) is one of the most common malignancies in transfusion-dependent thalassemia (TDT) patients, with main aggravating factors being iron overload and hepatitis viruses (HBV, HCV). This study aims to describe the computed tomography (CT) and magnetic resonance imaging (MRI) features of HCC in TDT patients.

**Material and Methods:** From a pool of 317 thalassemia patients, three experienced radiologists retrospectively analyzed in consensus, CECT and MRI studies positive for HCC from 2013 to 2023. Major HCC criteria (arterial enhancement, washout, enhancing capsule) and fat content were evaluated with both modalities. Additional MRI features, including T1w, T2w, diffusion restriction and hepatobiliary phase uptake, were evaluated. SPSS v29 was used for statistical analysis.

**Results:** A total of 20 patients were identified [F: 8, mean age: 50.9 years], 17 had HCV and 8 HBV. The highest incidence was between 48 and 51 years. Alpha-fetoprotein was positive in 20% cases, while the majority was well chelated, with 14 patients demonstrating normal liver MRI T2* values (≥16 ms) and mean MRI T2* value being 17 ms. 20% presented with multifocal disease. Almost all lesions showed typical HCC imaging features with category LI-RADS 5 attributed to 19 patients (95%). Their overall survival correlated negatively with the T stage at the time of diagnosis, with good statistical significance (log Rank, $p < 0.05$).

**Conclusion:** Despite the relatively small number of HCCs identified, we can testify that HCC developing in thalassemia patients as depicted on CECT and/or MRI demonstrates typical imaging features, while higher T stage leads to poorer overall survival.

### SS 1.6

### Qualitative MRI features for predicting pathological grade of hepatocellular carcinoma: a systematic review and meta-analysis

T. Yang, B. Song; *Chengdu/CN*

**Purpose:** Accurate preoperative assessment of the pathological grade of hepatocellular carcinoma (HCC) is important for prognosis. However, the reported significant magnetic resonance imaging (MRI) features for predicting pathological grade of HCC were variable and conflicting. Thus, this meta-analysis aimed to evaluate the diagnostic value of preoperative MR imaging features for predicting pathological grade of HCC.

**Material and Methods:** PubMed, EMBASE, and the Cochrane Library were searched to find related original articles published up to September 4, 2024. Study quality was assessed using QUADAS-2. The bivariate random-effects model was used to pool the diagnostic odds ratio (DOR), sensitivity, specificity and their 95% confidence interval (CI) for each significant qualitative MRI feature for predicting pathological grade of HCC.

**Results:** A total of 23 original studies with 1829 HCCs were included. Of the 18 available MRI features, 5 were significantly associated with high-grade HCC: larger tumor size (>5 cm) (DOR = 4.7, 95% CI [2.3–9.8]), diffusion restriction (3.9, 95% CI [1.9–7.3]), arterial peritumoral enhancement (2.5, 95% CI [1.2–4.7]), peritumoral hypointensity on hepatobiliary phase imaging (HBP) (5.8, 95% CI [2.4–11.2]), non-smooth tumor margin (3.2, 95% CI [2.0–4.6]).

**Conclusion:** 5 MRI features including larger tumor size, diffusion restriction, arterial peritumoral enhancement, peritumoral hypointensity on HBP, non-smooth tumor margin were significant predictors for high-grade HCC.

### SS 1.7

### Performance of simultaneous multislice diffusion-weighted imaging using monoexponential, intravoxel incoherent motion, and diffusion kurtosis models: assessment of microvascular invasion and histologic grade in hepatocellular carcinoma

Y. Wu, Z. Ye, B. Song; *Chengdu/CN*

**Purpose:** Diffusion-weighted imaging (DWI), intravoxel incoherent motion (IVIM), and diffusion kurtosis imaging (DKI) are valuable for predicting hepatocellular carcinoma (HCC) aggressiveness but are limited by long scan times. Simultaneous multislice (SMS) technology has demonstrated feasibility in reducing scan times for liver diffusion imaging. However, its impact on assessing HCC aggressiveness remains underexplored.

**Material and Methods:** This prospective single-center study, conducted from June 2021 to December 2023, included 77 HCC patients who underwent preoperative 3T MRI with SMS and conventional sequences for DWI, IVIM, and DKI. Diffusion parameters (ADC, D, D*, f, MD, MK) were compared between SMS and conventional methods. Diagnostic performance for predicting microvascular invasion (MVI) and histologic grade was evaluated using ROC curves. Inter-reader consistency was assessed using ICC.

**Results:** A total of 77 patients were analyzed, of whom 29.9% were MVI-positive and 35.1% had high-grade HCC. SMS acquisition reduced scan times by up to 44.44%. Diffusion parameters from SMS were largely comparable to those from conventional sequences, except for slightly lower ADC and perfusion fraction (f) in SMS. Significant differences in ADC, D, D*, and MK values were observed between MVI-positive and MVI-negative cases, as well as between high-grade and low-grade HCC (all $p < 0.001$). SMS-MK demonstrated the highest diagnostic accuracy (AUC = 0.92 for MVI, 0.86 for histologic grade). Inter-reader consistency for diffusion parameters showed good to excellent agreement, with ICC values ranging from 0.719 to 0.885.

**Conclusion:** SMS diffusion imaging integrated with IVIM and DKI provides a fast, reliable alternative for preoperative HCC evaluation, maintaining diagnostic accuracy while significantly reducing scan times.

### SS 1.8

### Radiomics analysis of preoperative contrast-enhanced CT of hepatocellular nodules undergoing robotic surgery: correlation with histopathological grade and recurrence risk

A. Pecchi[1], G. Besutti[2], L. Alemanno[1], C. Bozzola[1], C. Beretta[1], L. Nocetti[1], P. Magistri[1], F. Di Benedetto[1], P. Torricelli[1]; *[1]Modena/IT, [2]Reggio/IT*

**Purpose:** We aimed to determine whether and which radiomic features from CT images of HCC nodules undergoing robotic surgery correlate with the histopathological tumour grade and the recurrence risk.

**Material and Methods:** This retrospective study included patients histologically diagnosed with HCC who underwent preoperative multiphase contrast-enhanced CT and robotic surgery in 2014–2022. Arterial and venous phase images were manually segmented and features were extracted with Pyradiomics. The 5 most important features for pathological grade and recurrence risk were selected with Maximum Relevance Minimum Redundancy algorithm.

**Results:** We included 64 patients (4 grade 1 HCC, 35 grade 2, 25 grade 3) of which 21 relapsed during follow-up (3 among grade 1, 8 among grade 2, 10 among grade 3). Median progression-free survival was obtained for each histological subgroup, respectively 44.5 months in grade 1, 28 months in grade 2 and 25 months in grade 3. The best classifier for histological grade 2 and 3 was Logistic Regression, fitted with features extracted from both arterial and portal venous phases (AUC = 0.80, sensitivity = 0.72, specificity = 0.91). The most statistically significant radiomic features were correlated to uniformity and variations in the homogeneity of gray levels within the lesions. Regarding the recurrence risk, Logistic Regression performed better when fitted only with features from arterial phase (AUC = 0.77, sensitivity = 0.78, specificity = 0.76). Cluster Shade was higher on arterial phase in patients with post-operative recurrence, while Kurtosis was higher on portal venous phase in patients with no recurrence.

**Conclusion:** This study shows the feasibility of a radiomic analysis of HCC nodules and its potential role in assessing the histological grade and the recurrence risk.

### *SS 1.9*

### Fractal dimension analyses based on Gd-EOB-DTPA-enhanced MRI for prediction of vessels that encapsulate tumour clusters and recurrence in patients with hepatocellular carcinoma

F. Che, B. Song*; Chengdu/CN*

**Purpose:** Fractal analysis is a promising tool for measuring vascular development and cancer cell characterization. It enables quantitative evaluation of the hidden information under the complexity structure by calculating the fractal dimension (FD) and lacunarity. It provides a way to quantitatively evaluate the high complexity underlying structural geometry. Thus, the aim of this study was to assess the potential role of fractal analysis from Gd-EOB-DTPA-enhanced MRI for vessels that encapsulate tumour clusters (VETC) and recurrence-free survival (RFS) prediction in hepatocellular carcinoma (HCC) patients.
**Material and Methods:** We conducted a retrospective study including 361 HCC patients (training: 253, Validation: 108) with Gd-EOB-DTPA-enhanced MRI before surgery. The FD and lacunarity were extracted from the hepatobiliary phase of tumor by box-counting algorithms. Clinical and semantic image features were included and evaluated. Multivariate analysis identified the independent predictors for VETC, with nomogram visualized the ultimately predictive models. RFS was analysed by the Kaplan–Meier method.
**Results:** VETC-positive HCC was shown to have higher FD and lacunarity than VETC-negative HCC ($p < 0.01$). Elevated AFP, ALT, intratumor necrosis, FD and lacunarity were found to be associated with VETC. After multivariate analysis, intratumor necrosis, FD and lacunarity were used for the nomogram construction of VETC prediction. The AUCs of VETC nomogram reached 0.803 (95% CI: 0.747–0.858) in training cohort and 0.804 (95% CI: 0.724–0.884) in validation cohort. Patients with model-predicted VETC had significantly shorter RFS than those without.
**Conclusion:** The 3D FD quantitatively described the VETC status in HCC patients. The combined model can be used as a meaningful biomarker for VETC diagnosis and RFS prediction in HCC patients.

**09:00 – 10:30** **E 105/106**

### *Scientific Session SS 2*

### Colorectal cancer: diagnosis and liver metastases

### *SS 2.1*

### CT colonography for colorectal cancer prevention and detection: 20-year experience at a U.S.-based academic medical center

MV. Golden, D.H. Kim, P.J. Pickhardt*; Madison, WI/US*

**Purpose:** To report the unique CT colonography (CTC) experience at a single U.S. academic center.
**Material and Methods:** Over a 20-year period ending March 2024, 11,830 patients underwent a total of 15,431 CTC examinations, including initial colorectal evaluation in 10,147 adult outpatients (5,465 women, 4,682 men; mean age, 56.7 years), which comprise the primary study cohort. Records were reviewed for colorectal findings, including any optical colonoscopy (OC) correlation, and resulting histopathology. Advanced adenomas were defined by large size (≥10 mm) and/or high-grade dysplasia or villous component.
**Results:**
Initial CTC evaluation was positive for polyps ≥6 mm, 6–9 mm, ≥10 mm, and ≥3 cm in 16.5% (1673/10,147), 12.6% (1280/10,147), 6.3% (638/10,147), and 0.9% (88/10,147) of patients, respectively. Of the 2665 CTC-detected polyps ≥6 mm, 60.8% were sessile, 18.7% were flat, and 16.6% were pedunculated; 47.8% were right-sided and 52.2% were left-sided. 647 patients with 6–9 mm polyps opted for CTC surveillance. Of the 1682 positive cases that eventually underwent OC, concordant lesions were found in 1539, corresponding to a PPV of 91.5%. The most aggressive-proven pathology per patient was invasive cancer (n = 39), advanced adenoma (n = 431), tubular adenoma (n = 344), sessile serrated lesion (n = 52), hyperplastic polyp (n = 123), and other benign pathology (n = 38). There were no colonic perforations or other major complications. Proven unsuspected extracolonic cancers were detected in 42 patients and AAAs were detected in 41 patients.
**Conclusion:** CT colonography represents a robust, non-invasive screening option that safely and effectively provides for both colorectal cancer prevention and detection, as well as extracolonic evaluation.

### *SS 2.2*

### Development and validation of preoperative CT-based stacking model for classifying colorectal polypoid lesions

X. Meng, R. Duan, L. Zheng*; Guangzhou/CN*

**Purpose:** Accurate preoperative staging of colorectal polypoid lesions is complex but essential. While treatment for adenoma/Tis and T2-4 lesions is well defined, managing T1 cancers requires further pathological evaluation. Therefore, we aim to develop a CT-based three-class classification model to support clinical decision-making.
**Material and Methods:** In this retrospective study, patients with polypoid lesions identified on CT were enrolled from two medical centers between November 2017 and November 2022. Radiomic features were extracted from contrast-enhanced CT images on tumor and peri-tumoral mesenteric regions. LASSO regression was applied for feature selection. Six classifiers were utilized to develop distinct evaluation models, and their output probabilities were integrated into a BPNet-based stacking model. Additionally, clinical and radiological features were incorporated into a multinomial logistic regression model, and its output probabilities were combined with the stacking model to form a hybrid model. Diagnoses were given by a senior radiologist and confirmed via postoperative pathology. The performance of models and the radiologist was assessed and compared.
**Results:** A total of 1032 patients were enrolled (mean age, 56.74 ± 11.60 years; 445 females) and divided into train set (n = 664), validation set (n = 285), external test set (n = 83). In the validation and external test sets, the hybrid model (micro-average AUC = 0.82, 0.78) significantly outperformed the senior radiologist (micro-average AUC: 0.82 vs 0.73, $p < 0.01$; 0.78 vs 0.62, $p < 0.01$) in the three-class classification task.
**Conclusion:** The stacking model can effectively differentiate adenoma/Tis, T1, and T2-4 lesions, offering limited yet valuable guidance for managing colorectal polypoid lesions.

### *SS 2.3*

### Improving colon cancer staging accuracy: a feasibility study on MRI utilizing ACS technology

Z. Guan, Y. Sun*; Beijing/CN*

**Purpose:** AI-assisted Compressed Sensing (ACS) technology has been successfully applied to improve scanning speed and image quality in MRI. However, its potential in colon MRI remains unexplored. This study aims to compare the image quality and diagnostic performance of ACS-MRI and CT for colon cancer.
**Material and Methods:** 33 colon cancer patients who underwent both CT and colon MRI scans at our hospital were prospectively enrolled. MRI was performed with a 3.0-T MRI scanner by using super soft body coil in the supine position. Oblique axial T2-weighted imaging (T2WI) was obtained. The image quality was evaluated by contrast-to-noise ratio (CNR) and sharpness. And the TN staging was also assessed using venous phase CT images and oblique axial T2WI. The Mann–Whitney U test compared CNR and sharpness differences between ACS-T2WI and CT. Diagnostic performance for tumor and lymph-node stage was calculated for both imaging techniques.
**Results:** The CNR was higher for ACS-T2WI than CT (4.71 ± 2.03 vs. 1.32 ± 0.72, $P < 0.001$). ACS-T2WI had lower ERD values compared to CT (2.94 ± 2.22 vs. 5.23 ± 1.91, $P < 0.001$). The accuracy, over-staging, and under-staging rates of ACS-T2WI assessed tumor staging were 78.8%, 18.2%, and 3.0%, respectively. For CT-assessed tumor staging, the accuracy, over-staging, and under-staging rates were 30.3%, 60.6%, and 9.1%, respectively. Regarding lymph-node staging, ACS-T2WI achieved an accuracy of 81.8%, sensitivity of 72.2%, and specificity of 93.3%. In contrast, CT had an accuracy of 51.5%, sensitivity of 83.3%, and specificity of 13.3% for lymph-node staging.
**Conclusion:** Colon MRI demonstrates excellent image quality, further improving the accuracy of preoperative staging in colon cancer.

### *SS 2.4*

### Pre-operative angio-CT colonography for right colectomy for cancer

M. Cadi, A. Cazelles, C. Labiad, F. Lecot, A. Mariani, M. Karoui, G. Manceau*; Paris/FR*

**Purpose:** Right hemicolectomy with lymph node (LN) dissection and complete mesocolic excision is the standard-of-care for patients with right-sided colon cancer but remains challenging. Pre-operative angio-CT colonography (A-CTC) may both improve the oncological quality of resection and decrease the risk of vascular injuries, as it provides useful information on the precise location of the tumor and detailed mesenteric vascular mapping. The aim of the study was to evaluate A-CTC as a pre-operative imaging modality prior to laparoscopic right hemicolectomy for cancer.
**Material and Methods:** Between 01/2020 and 12/2023, 161 patients underwent minimally invasive right colectomy for cancer. Among them, 44 were evaluated with A-CTC state-of-the-art technique combined with an IV contrast. The post-processing images consisted of fusing 3D arteriogram with 3D venogram and the whole colon to obtain 3D images. Among the 44 patients, the optical colonoscopy performed between 6 and 12 months was obtained for 30 patients.
**Results:** Histologically, the median number of harvested LN on the surgical specimen was 24, with 100% of included patients having more than 12 LN. In these 44 patients, A-CTC contributed to a change in the initial surgical plan for four imprecise tumor location (9%). A-CTC detected all tumors, and their precise location was confirmed during surgery. No intraoperative bleeding complication was reported. The follow-up endoscopic examination did not find any colon cancer and advanced adenomas.
**Conclusion:** Pre-operative 3D A-CTC may improve the quality of LN dissection during minimally invasive right hemicolectomy for cancer. Further comparative studies are still needed to confirm these preliminary results.

### *SS 2.5*

### CT-based radiomics for survival prediction in colorectal liver metastases: beyond clinical scores

A. Ammirabile, G. Matteucci, E. Ragaini, E. Lanza, M. Francone, G. Torzilli, F. Ieva, L. Viganò*; Milan/IT*

**Purpose:** Surgery with perioperative chemotherapy is the standard treatment for colorectal liver metastases (CRLM). Patient selection should rely on survival prediction, but available prognostic factors and scores have limited reliability. This study investigated the potential of CT-based radiomics for survival prediction in CRLM patients, focusing on its performance according to the CT-surgery interval and comparison with clinical scores.
**Material and Methods:** This single-center retrospective study included all consecutive patients undergoing liver resection for CRLM between 2010 and 2020. The main inclusion criterion was the availability of a high-quality CT scan performed ≤60 days before surgery with a detectable CRLM ≥10 mm. Manual tumor segmentation (Tumor-VOI) and automatic 5-mm peritumoral expansion (Margin-VOI) were performed on the portal phase of CT scans. A survival random forest approach with internal validation was used to build predictive models of overall survival (OS). The impact of the CT-surgery interval (> vs. ≤30 days) on model performance was analyzed.
**Results:** 306 patients were included (mean age 63 years) with a five-year OS rate of 40.9% (mean follow-up 34 months). The clinical model had a C-index of 0.627 and, in the entire cohort, radiomic data did not improve model performance. In the 212 patients with a CT-surgery interval ≤30 days, the Tumor-VOI radiomic model achieved a C-index of 0.692 and combining Tumor-VOI and Margin-VOI features increased the C-index to 0.718. The full clinical-radiomic model outperformed standard prognostic scores (Fong/GAME/c-MS; C-indices = 0.536–0.598).
**Conclusion:** Radiomic features of CRLM and peritumoral tissue from preoperative CT scans improve survival prediction, outperforming standard clinical scores. A shorter CT-surgery interval impacts performance, being associated with better prediction.

### *SS 2.6*

### MR imaging of fibrosis in colorectal liver metastases: late gadolinium enhancement is associated with overall survival in patients with CRLM

I. Morawska*; Warsaw/PL*

**Purpose:** This study aimed to assess whether progressive gadolinium enhancement on MRI of colorectal liver metastases (CRLM) following chemotherapy correlates with tumor fibrosis and associates with overall survival.
**Material and Methods:** A retrospective group of 98 patients (69 M, mean age 62.87 ± 10.73 years) undergoing preoperative gadobenate dimeglumine-enhanced MRI (2018–2023) after chemotherapy was analysed. Progressive target tumor enhancement (TTE), calculated as the mean contrast-to-noise ratio of up to two target lesions before contrast injection, through late arterial phase, until 5-min and 60-min delayed MRI, was independently assessed by two radiologists. Correlations between TTE and tumor fibrosis (histopathology) were evaluated using Spearman analysis, while Kaplan–Meier and Cox regression assessed the impact of TTE on overall survival.
**Results:** In our group, TTE significantly correlated with tumor fibrosis ($r = 0.45$, $p < 0.001$). Strong TTE was associated with improved 3-year survival (85.2% vs. 53.4%, $p = 0.005$), with a hazard ratio of 0.34 (95% CI: 0.16–0.85, $p = 0.006$) after adjusting for prognostic factors.
**Conclusion:** Late gadolinium MRI enhancement of CRLM after chemotherapy is associated with tumor fibrosis and overall survival. These findings support the integration of advanced MR imaging techniques in the routine evaluation of patients with CRLM, offering a valuable tool for improving treatment stratification and clinical outcomes.

### SS 2.7

### Total tumor volume predicts overall survival and response to systemic induction chemotherapy in patients with colorectal cancer liver metastases: an ancillary study of the randomized phase 3 CAIRO5 trial

M. Ali[1], M. Zeeuw[1], R. Kemna[1], S. van Eck[1], N. Wesdorp[1], J. Roor[2], J. Van Waesberghe[1], J. van den Bergh[1], I. Nota[1], S. Moos[1], S. van Dieren[1], M. van Amerongen[3], M. Bond[4], T. Chapelle[5], R. van Dam[6], M. Engelbrecht[1], M. Gerhards[1], D. Grunhagen[7], T. van Gulik[1], J. Hermans[3], K. de Jong[8], J. Klaase[8], N. Kok[1], W. Leclercq[9], M. Liem[10], K. van Lienden[11], I. Molenaar[4], G. Patijn[12], A. Rijken[13], T. Ruers[1], C. Verhoef[7], J. de Wilt[3], T. Buffart[1], R.-J. Swijnenburg[1], C. Punt[4], I. Verpalen[1], J. Stoker[1], J. Huiskens[1], G. Kazemier[1]; *[1]Amsterdam/NL, [2]Huizen/NL, [3]Nijmegen/NL, [4]Utrecht/NL, [5]Antwerp/BE, [6]Maastricht/NL, [7]Rotterdam/NL, [8]Groningen/NL, [9]Veldhoven/NL, [10]Enschede/NL, [11]Nieuwegein/NL, [12]Zwolle/NL, [13]Breda/NL*

**Purpose:** Total tumor volume (TTV) may better reflect objective tumor burden and treatment response compared to the currently used RECIST1.1 criteria, which are limited by their subjective and one-dimensional nature. This study aimed to compare the prognostic value of TTV versus RECIST1.1 for overall survival (OS) in patients with colorectal cancer liver metastases (CRLM) and to assess whether TTV could guide optimal systemic treatment selection for individual patients.

**Material and Methods:** Patients with unresectable liver-only CRLM from the CAIRO5 trial (NCT02162563) who received induction systemic treatment were included. Baseline TTV, change in TTV after systemic treatment, and RECIST1.1 were calculated using the CT scans before systemic treatment and at first follow-up, and were assessed for their prognostic and predictive values with multivariable Cox regression models.

**Results:** In total, 425 patients were included. Baseline TTV (adjusted HR [aHR] for 100 mL vs 10 mL, 2.44 [95% CI, 1.25–4.76]; P = 0.006) and relative change in TTV were the strongest predictors for OS (aHR for 0% change vs 50% decrease, 2.57 [1.83–3.60]; P < 0.0001). In contrast, RECIST1.1 was not independently associated with OS (aHR for partial response vs progressive disease, 0.63 [95% CI, 0.33–1.20]). Higher baseline TTV was predictive for a stronger treatment benefit of FOLFOX-/FOLFIRI-bevacizumab versus FOLFOX-/FOLFIRI-panitumumab on OS ($P_{interaction}$ = 0.017).

**Conclusion:** TTV is strongly prognostic for OS in CRLM patients receiving systemic treatment, while RECIST1.1 was not. Therefore, change in TTV may better assess treatment response compared to RECIST1.1. Moreover, baseline TTV might guide personalized treatment decision-making between bevacizumab and panitumumab.

### SS 2.8

### Prognostic value of circulating tumor DNA and radiological total tumor volume in patients with initially unresectable colorectal liver metastases

R. Kemna[1], N. Crnovrsanin[1], M. Zeeuw[1], M. Ali[1], B. Alipanahi[2], K. Lumbard[2], Z. Skidmore[2], L. Rinaldi[2], I. van 't Erve[1], N.J. Wesdorp[1], J. Huiskens[1], D. van Steijn[1], J. van Waesberghe[1], J. van den Bergh[1], I. Nota[1], S. Moos[1], M. Bond[3], L. Meiqari[1], I. Huitink[1], E. Giovannetti[1], J. Stoker[1], I. Verpalen[1], D. van den Broek[1], G. Meijer[1], R.-J. Swijnenburg[1], C. Punt[3], R. Scharpf[2], A. Leal[2], N. Dracopoli[2], V. Velculescu[2], N. Kok[1], G. Kazemier[1], R.J. Fijneman[1]; *[1]Amsterdam/NL, [2]Baltimore, MD/US, [3]Utrecht/NL*

**Purpose:** Computed tomography (CT) treatment response assessment is crucial in unresectable colorectal liver metastases (CRLM). Volumetric total tumor volume (TTV) therefore outperforms RECIST. CRLM has a significant molecular heterogeneity, and combining CT evaluation with circulating tumor DNA (ctDNA) could improve risk stratification. We studied the (combined) prognostic value of TTV and circulating tumor DNA (ctDNA) levels in patients with initially unresectable CRLM.

**Material and Methods:** 101 patients who received FOLFOX/FOLFIRI and bevacizumab for initially unresectable liver-only CRLM and participated in the phase 3 CAIRO5 trial (NCT02162563) were included. TTV was measured using semi-automatic segmentation of all metastases CT scans. ctDNA was assessed using DNA Evaluation of Fragments for early Interception-Tumor Fraction (DELFI-TF). Primary outcome was the prognostic value for overall survival (OS).

**Results:** At baseline, we found that measure of TTV (median 139 mL, IQR 23–497 mL) correlated with levels of and DELFI-TF (median 0.29, IQR 0.13–0.41, Spearman's ρ = 0.70 [95% CI, 0.57–0.79]). After systemic treatment, there was no correlation (Spearman's ρ = 0.09 [−0.01–0.42]), due to differences in dynamic changes. In multivariable analyses, baseline DELFI-TF (P = 0.001) and TTV (P = 0.002) and their change after systemic treatment were the strongest independent predictors of OS, outperforming current clinicopathological factors. The prognostic value of TTV on OS changes was dependent on DELFI-TF levels ($P_{interaction}$ = 0.002).

**Conclusion:** Combining TTV and ctDNA could improve risk stratification and systemic treatment response monitoring in patients with initially unresectable CRLM.

Scientific Sessions

Authors' Index

### SS 2.9

### Evaluation of treatment response to chemotherapy in patients with colorectal liver metastases undergoing liver transplantation

M. Vaiani, C. Angelucci, V. Magni, M. Maspero, C. Sposito, T. Cascella, V. Mazzaferro; *Milan/IT*

**Purpose:** To evaluate treatment response to chemotherapy in patients with colorectal liver metastases (CRLM) who subsequently underwent transplantation, correlating with histopathological assessment of tumor regression grade (TRG) in the explanted liver.

**Material and Methods:** We retrospectively assessed the radiological response of patients enrolled and transplanted within the COLT trial (NCT03803436) between 2019 and 2024. Tumor burden on available CTs and MRIs between trial enrollment and transplantation was assessed with three scoring systems: depth of response (DPR), tumor burden score (TBS) and RECIST. Measurements were conducted on CT portal venous phase using the Syngo.via automatic contouring system (Siemens Healthineers). The scoring systems were correlated with TRG.

**Results:** Sixteen patients were enrolled (44% female, median age 55 years). Ten (62.5%) had TRG ≤ 3. All had CT or MRI imaging available at trial enrollment, showing unresectable CRLM, pre-transplant CT scans and explant pathology. Median DPR was 94%, with 9 (56%) patients having >90% DPR. Median RECIST response was 59%. Median TBS response was 57%. In patients with DPR > 90%, TRG was ≤3 in 7 (78%) cases, while in patients with DPR ≤ 90%, TRG was ≤3 in 2 (29%) patients (p = 0.045). No correlation was present between TRG and RECIST or TBS response.

**Conclusion:** The study demonstrates that the DPR scoring system showed the strongest correlation with the tumor regression grade (TRG) in the explanted liver, particularly in identifying patients with a higher degree of tumor response (TRG < 4).

### SS 2.10

### Liver metastases and hepatocellular carcinoma: visualization with manganese-based orally administered contrast agent-enhanced liver MRI in patients with severe kidney disease—evidence from the SPARKLE study

N. Kartalis[1], A. Norlin[2], N. Hettiarachchie[3], V. Lucidi[4], T. Brismar[1], D. Geisel[5]; *[1]Stockholm/SE, [2]Malmo/SE, [3]Copenhagen/DK, [4]Bologna/IT, [5]Berlin/DE*

**Purpose:** To assess the improvement in visualization on MRI of focal liver lesions (FLL) in the subset of patients included in the SPARKLE study with liver metastases (LM) or hepatocellular carcinoma (HCC) after the ingestion of Ascelia Pharma manganese-based contrast agent (ACE-MBCA ) compared to unenhanced MRI (UMRI).

**Material and Methods:** In the SPARKLE study, patients (n = 85) with FLLs and severe kidney impairment received liver MRI before and 4 ± 1 h after the ingestion of ACE-MBCA. In the subset of patients with LM and HCC, visualization of FLL was evaluated by 3 independent readers (R1, R2, R3) by assigning a qualitative score to two co-primary variables [lesion contrast (LC) and border delineation (BD)] (scores from 1 = poor to 4 = excellent) in UMRI and combined MRI (CMRI). For the analysis, only FLLs matched between UMRI and CMRI were used. The LM-subgroup included n = 25 (R1), n = 23 (R2), and n = 26 (R3), and the HCC-subgroup n = 11 (R1), n = 10 (R2) and n = 10 (R3) patients. The hypothesis for the superiority of CMRI over UMRI was tested using a 1-sided paired t-test with the significance level set at 0.05.

**Results:** For the overall population, LC and BD improved significantly in CMRI compared to UMRI for all 3 readers (all, p < 0.001). Similarly, in the subgroup analysis, the mean LC-scores for UMRI/CMRI were 2.49/3.47 (R1), 2.84/3.86 (R2), and 2.51/3.33 (R3), and the mean BD-scores for UMRI/CMRI were 2.51/3.46 (R1), 3.00/3.80 (R2), and 2.31/2.97 (R3), respectively (all, p < 0.05).

**Conclusion:** ACE-MBCA significantly improves visualization on MRI of LM and HCC compared to unenhanced MRI in patients with severe kidney impairment.

**10:00 – 10:45** **Emerald Room**

*Scientific Session SS 15*

### Pancreatic cysts and more

### SS 15.1

### The greater mimicker of high-risk branch duct type intraductal papillary mucinous neoplasm

S. Youn, H. Kim, S. Jeon, J. Kim; *Seoul/KR*

**Purpose:** To evaluate key imaging features for differentiating serous cystadenoma (SCA) from branch duct intraductal papillary mucinous neoplasm (BD-IPMN) and to assess the diagnostic performance of CT and MRI.

**Material and Methods:** A total of 238 patients with SCA (n = 117) and BD-IPMN (n = 121) who underwent surgery from 2004 to 2023 were retrospectively included. Two radiologists assessed image findings of pancreatic cysts and the likelihood of SCA or BD-IPMN on a 5-point scale. Key features were identified through univariable analysis, with variables showing p < 0.05 included in a multivariate logistic regression analysis. The diagnostic performance of CT alone and combined CT/MRI was evaluated using receiver operating characteristic analysis.

**Results:** Multivariate analysis of CT/MRI identified body/tail rather than head/uncinate [Hazard ratio (HR), 4.767; p = 0.013] and thickened cyst wall [HR, 14.547; p = 0.002] as predictors for SCA. For BD-IPMN, predictors included older age [HR, 1.101; p = 0.001], multiplicity [HR, 4.172; p = 0.020], pleomorphic rather than honeycomb or multicystic shape [HR, 7.891; p = 0.001], and p-duct communication [HR, 13.596; p < 0.001]. Enhancing mural nodule [HR, 16.149; p < 0.001] and central scar [HR, 10.484; p = 0.017] were significant predictors for SCA on CT alone. Diagnostic performance was higher with combined CT/MRI than with CT alone (R1, area under the curve (AUC), 0.862 vs. 0.956, p < 0.001; R2, AUC, 0.852 vs. 0.893, p = 0.090).

**Conclusion:** CT and MRI findings are useful for distinguishing SCA from BD-IPMN, with key imaging features. MRI offers additional diagnostic value over CT alone for differentiating between SCA and BD-IPMN.

### SS 15.2

### Intraductal papillary mucinous neoplasms follow-up: the story from the MR suite of Verona Pancreas Center

B. Mascarin, E. Bardhi, F. Spoto, N. Cardobi, R. De Robertis, M. D'Onofrio; *Verona/IT*

**Purpose:** To evaluate the rate of malignant modification during MRI follow-up of intraductal papillary mucinous neoplasms (IPMN), analyzing if the current approach proposed in 2024 Kyoto guidelines is effective.

**Material and Methods:** This retrospective analysis included 1400 patients diagnosed with IPMN at the University Hospital of Verona during 20 years. These patients underwent MRI follow-ups, totaling 6165 contrast-enhanced MRI. The characteristics of IPMN (number of cysts, location, size), radiological and histological modification data were studied and compared. Chi-square test and independent samples t-tests were used for statistical evaluations.

**Results:** Among the 1400 patients, 118 (8.2%) showed radiological modifications, with 66 patients showing signs of modification at the first MRI, 27 occurring within the first 5 years and 25 after the fifth year of follow-up (p value > 0.05). Patients with radiological changes had a mean lesion size increase of 4.2 mm, compared to 1.3 mm in those without changes (p value < 0.05). Among those who exhibited radiological signs of modification within the initial five years of follow-up, 7/27 showed low-grade dysplasia (LGD) and 13/27 showed high-grade dysplasia (HGD). 4/27 patients were diagnosed with pancreatic ductal adenocarcinoma (PDAC), with 3 having synchronous PDAC and 1 having concomitant PDAC. Among patients who showed modifications from the fifth year onward, 11/25 exhibited dysplasia (10 with LGD, 1 with HGD) and 8/25 were diagnosed with PDAC, all located at the same anatomical site as the IPMN. One patient was histologically negative.

**Conclusion:** The risk of progression remains consistent regardless of the number of years and MR examinations. Individualized follow-up strategies are crucial to optimize patient management, avoiding overtreatment.

### SS 15.3

### Multiphasic spectral absorption curve measurements for differentiating between mucinous and non-mucinous pancreas cyst fluids

I. Dudás, Z. Somogyiné Nagy, B. Lovász, A.I. Vigh, Á. Szücs, M. Benke, P. Kaposi, A. Szijártó, P. Maurovich-Horvat, B. Budai; *Budapest/HU*

**Purpose:** Differentiating pancreatic cystic lesions (PCLs) remain a diagnostic challenge. Spectral imaging of photon-counting detector CT (PCD-CT) scanners enables the reconstruction of different keV value virtual monoenergetic images (VMI), allowing for spectral measurements. This study evaluated whether mucinous and non-mucinous PCLs exhibit distinct spectral absorption patterns on postcontrast scans, which could aid diagnostics.

**Material and Methods:** Our study included 53 mucinous cystic neoplasms and 23 non-mucinous PCLs all of which had arterial, pancreatic, and venous phase PCD-CT scans available. The densities were measured on the 40 keV and 70 keV VMIs by placing three circular regions of interest within the cyst fluid on each scan. The difference of the average measures was calculated (HUΔ40–70 keV) for each postcontrast phase. Group comparisons were performed using the Mann–Whitney U test, and diagnostic performance was assessed using ROC curve analysis.

**Results:** Mucinous PCLs showed significantly higher HUΔ40–70 keV values than non-mucinous PCLs for arterial ($p = 0.017$), pancreatic ($p < 0.0001$), and venous phases ($p < 0.0001$). The HUΔ40–70 keV yielded equally great performance on the pancreatic and venous phases with AUCs of 0.93 and 0.86; accuracies of 82% and 83%; sensitivity of 75% and 85% and specificity of 96% and 78%. Meanwhile, the arterial phase resulted in an AUC of 0.65, accuracy of 71%, sensitivity of 75%, and specificity of 61%.

**Conclusion:** Measuring the HUΔ40–70keV of cyst fluids on VMI reconstructions could be a promising non-invasive complementary approach for differentiating mucinous from non-mucinous PCLs.

### SS 15.4

### Prevalence and distribution of calcifications in the differential diagnosis of pancreatic lesions

F. Spoto, R. De Robertis Lombardi, N. Cardobi, B. Mascarin, F. Pasquazzo, M. D'Onofrio; *Verona/IT*

**Purpose:** To evaluate the incidence and pattern of calcifications in pancreatic neoplasms that commonly calcify, including serous cystadenomas (SCAs), solid pseudopapillary neoplasms (SPNs), neuroendocrine neoplasms (NENs) and mucinous cystic neoplasms (MCNs), on unenhanced CT and their potential role in differential diagnosis.

**Material and Methods:** Patients with pancreatic NENs, SCAs, MCNs and SPNs were retrospectively identified. Inclusion criteria were diagnosis established by pathology or endoscopic ultrasound and MR follow-up ≥1 year. Two radiologists reviewed unenhanced CT to evaluate lesion location, lesion size, presence of calcifications and pattern of calcifications (type 1, intratumoral punctate calcifications; type 2, curvilinear/elongated calcifications; type 3, coarse intratumoral calcifications).

**Results:** 311 patients were included, with a mean age of 61 ± 14 years and female prevalence (56.9%). Calcifications were found in 27.7% of the lesions. Calcified NENs and SPNs were more frequent in the body/tail than in the head ($p = 0.003$); calcified NENs were larger than non-calcified ones ($p < 0.001$). Type 1 calcifications were more common among NENs, while those of type 3 were more common among SCAs. Type 3 calcifications decreased the likelihood of NEN diagnosis and increased the likelihood of SCA diagnosis; sensitivity, specificity, accuracy and AUC-ROC of the models were 89.1%, 41.9%, 72.1% and 0.891 for NENs and 33.3%, 98.6%, 87.2% and 0.986 for SCAs, respectively.

**Conclusion:** Calcifications are found in approximately 30% of pancreatic NENs, SCAs, MCNs and SPNs, and different patterns of calcification seen on unenhanced CT may help differentiate pancreatic neoplasms, thus helping to optimize patient management.

### SS 15.5

### The prediction of pancreatic dysfunction with T1 mapping in diabetic patients

F. Kulali, G. Guner; *Istanbul/TR*

**Purpose:** We aimed to investigate the role of pancreatic T1 relaxation time in the prediction of early pancreatic dysfunction in diabetic patients.

**Material and Methods:** A total of 84 patients (43 diabetic patients with a mean age of 61 ± 10 years and 43 non-diabetic patients with a mean age of 50 ± 13 years) with abdominal MRI examinations including T1 mapping during a retrospective 24-month period were reviewed by two radiologists. Pancreatic T1 relaxation times were measured in head, corpus and tail at non-contrast images. In all patients, amylase and lipase levels were noted for evaluation of pancreatic function. The relationship between T1 relaxation times and lipase levels was analyzed by Mann–Whitney U test. ROC analysis was performed to estimate an optimal cut-off value for detection of pancreatic dysfunction. Interobserver correlation was evaluated by Pearson coefficient.

**Results:** Of 84, 43 were diabetic (25 men and 18 women) and 43 were non-diabetic patients (13 men and 30 women). T1 relaxation times were found statistically higher in diabetic patients compared to non-diabetic patients ($p = 0.007$, $p < 0.05$). The mean T1 relaxation time of diabetic and non-diabetic group was 708.95 ± 151 ms, and 625 ± 68 ms, respectively. Elevated lipase levels were correlated with high T1 relaxation times ($p = 0.007$, $p < 0.05$). Optimal cut-off value of >700 ms was estimated with high specificity (88%) and accuracy (76%) ratios (AUC = 0.957). Strong interobserver correlation was found ($r = 0.75$, $p < 0.05$).

**Conclusion:** T1 relaxation time with a cut-off value of >700 ms is a reliable non-invasive indicator for predicting early pancreatic dysfunction in diabetic patients.

**11:00 – 12:30** **E 103/104**

*Scientific Session SS 3*

### HCC: response evaluation and prognosis

### SS 3.1

### Comparison of radiological response criteria in patients with hepatocellular carcinoma undergoing immunotherapy treatment

M. Milazzo, R. Cannella, F. Vernuccio, G. Brancatelli; *Palermo/IT*

**Purpose:** To compare treatment response evaluations using RECIST 1.1, mRECIST, and LI-RADS criteria in patients with advanced hepatocellular carcinoma (HCC) treated with atezolizumab and bevacizumab.
**Material and Methods:** This retrospective study included patients with advanced HCC who underwent systemic therapy every three weeks between March 2022 and May 2024, with available contrast-enhanced CT and/or MRI. Baseline and follow-up imaging were reviewed to assess treatment response evaluated by RECIST 1.1 (based on tumor size), mRECIST (viable tumor on arterial phase imaging), and LI-RADS (CT/MRI TRA Nonradiation).
**Results:** Thirty-four patients were included, with a median age of 68.2 years (42–85 years), of which 28 were men. Fifteen had infiltrative HCC with a mean size of target lesions measuring 6.4 cm (1.4–18.9 cm). LR-5 and LR-TIV were assigned to 10 (29.4%) and 16 (47%) lesions at the baseline, respectively. At the first follow-up (mean: 109.5 days), RECIST 1.1 categorized responses as partial response in 26.7%, stable disease in 50%, and progressive disease in 23.3%, while mRECIST reported partial response in 40%, stable disease in 40%, and progressive disease in 20%. LI-RADS categorized 96.7% of lesions as viable. By the second follow-up, concordance between criteria increased, with 4.2% complete response, 20.8% partial response, 50% stable disease, and 25% progressive disease for both RECIST 1.1 and mRECIST.
**Conclusion:** mRECIST criteria may suggest an earlier response to immunotherapy in patients with advanced HCC compared to RECIST 1.1. However, the survival implications of these discrepancies still need to be explored.

### SS 3.2

### Efficacy and evaluation of response in HCC treated with stereotactic body radiotherapy

T. Falcone[1], F. Castagnoli[2], A. Villanacci[3], B. Frittoli[1], D.-M. Koh[2], L. Grazioli[1]; *[1]Brescia/IT, [2]London/UK, [3]Rome/IT*

**Purpose:** To evaluate changes in apparent diffusion coefficient (ADC) values, size, wash-in, wash-out, T2-weighted (T2w) signal intensity, capsule and necrosis in patients with HCC treated with stereotactic body radiotherapy (SBRT).
**Material and Methods:** 62 patients with HCC, treated with SBRT, who underwent baseline gadoxetate-enhanced MRI within 4 weeks before treatment and follow-up MRI within 3 months after finishing SBRT were prospectively enrolled (October2014–January2022); 44 patients underwent a further follow-up with gadoxetate-enhanced MRI 12 months after SBRT. 3 radiologists evaluated, in consensus, tumour size (δ), ADC values, T2w signal characteristics, presence/absence of wash-in, wash-out, capsule and necrosis of all lesions. Local control (LC) was evaluated after a median follow-up of 2.1 years. Delta (Δ)δ and ΔADC were correlated using Spearman correlation coefficient. Δδ and ΔADC were compared (Mann–Whitney test) between patients with and without (n = 5) LC. A logistic regression model was trained to estimate the probability of response of patients using categorical imaging variables.
**Results:** The median ADC for HCC ($10^{-3}$ $mm^2/s$) and dimension were 0.94 and 22.5 mm, respectively, for baseline. The ADC values increased by 41.1% for HCC ($p < 0.001$), whereas tumour size decreased by 52.7% ($p < 0.001$). At 3-month FU, there was a significant difference in Δ(δ) and change in wash-out between patients with and without LC (p = 0.05; p = 0.03). At 12-month FU, there was a significant difference in Δ(δ) and change in wash-out between patients with and without LC (p = 0.04; p = 0.02). No significant correlation was found for ΔADC and for changes in wash-in, T2w signal intensity, necrosis and capsule between patients with and without LC at both 3- and 12-month FU.
**Conclusion:** SBRT showed excellent LC for HCC, which was associated with the degree of size change after treatment and the absence of wash-out at follow-up.

### SS 3.3

### Evaluation of radiological response to transarterial chemoembolization in patients who underwent liver transplantation for hepatocellular carcinoma: a multicenter cohort study

G. Porrello[1], C. Hobeika[2], C. Francoz[2], F. Cauchy[3], M. Bouattour[3], K. boudjema[4], D. Pietrasz[5], K. Mohkam[6], O. Scatton[3], S. Chopinet[7], A. Laurent[5], L. Barbier[7], V. Paradis[2], M. Ronot[2], V. Vilgrain[2], M. Dioguardi Burgio[2]; *[1]Palermo/IT, [2]Clichy/FR, [3]Paris/FR, [4]Rennes/FR, [5]Creteil/FR, [6]Lyon/FR, [7]Marseille/FR*

**Purpose:** To evaluate the efficacy of transarterial chemoembolization (TACE) for hepatocellular carcinoma (HCC) as a bridging therapy in liver transplantation (LT) recipients.
**Material and Methods:** We analyzed 386 patients who underwent LT for HCC from 8 centers and ≥1 pre-LT TACE between 2012 and 2017. Radiological response was assessed using modified Response Evaluation Criteria in Solid Tumors. Survival regressions were Cox models with center-specific cluster-robust variance. A nodule-by-nodule analysis (NBN), including patients with ≤2 HCCs, was performed using a multilevel linear regression.
**Results:** There were 328 (85%) males, with the median age of 59 (interquartile range, IQR: 54, 63) years. Median number of HCCs was 2 [IQR: 1, 3], and the median sum of the target lesions size was 41 mm [IQR: 30, 51]. The response after TACE was complete, partial, stable, or progressive in 36.8%, 32.9%, 10.1%, and 20.2% patients, respectively. Five years after LT, 87% patients were free of recurrence and the estimated disease-free survival (DFS) was 76%. Response to TACE was independently associated with DFS [hazard ratio (HR) partial vs. complete: 2.04 (95% confidence interval, CI: 1.17, 3.55); stable vs. complete: 3.98 (95%CI: 2.39, 6.64); progressive vs. complete: 7.65 (95%CI: 4.27, 13.7); all $p < 0.01$]. NBN analysis included 281 HCCs nested in 239 patients; tumor downsizing correlated with pathological necrosis [correlation coefficient (r): −0.51 (95%CI: −0.59, −0.42)], $p < 0.001$). A 10% downsizing was associated with DFS (conditional HR 0.94; 95%CI: 0.92, −0.95 $p < 0.001$). Downsizing was independently associated with embolization (p = 0.047) and super-selective approach (p = 0.036).
**Conclusion:** The prognosis of patients with LT for HCC is associated with TACE response. A selective approach and embolization optimize the downsizing of targeted HCC.

### SS 3.5

### Preoperative assessment of microvascular invasion in HCC based on preoperative imaging features

F. Salahshour, M. Mehrabi Nejad, N. Ayoobi Yazdi, M. Safaei, M. Taher, A. Kasraian, S. Seifouri, A.M. Moradi; *Tehran/IR*

**Purpose:** To identify clinical and preoperative imaging characteristics that predict microvascular invasion (MVI) in the histopathology of HCC.
**Material and Methods:** This retrospective study was conducted at a tertiary referral hospital. Patients with hepatic lesions who underwent liver transplantation with histopathologically confirmed MVI and preoperative CT or MRI imaging since 2013 were included. Exclusion criteria were prior treatments, age below 18 years, or indeterminate histopathology results. Clinical and imaging variables were recorded for all patients, and their association with histopathologically confirmed MVI was analyzed.
**Results:** A total of 47 patients (mean age: 52.95 ± 11.04 years; 78.7% male) with 122 liver lesions were included in the analysis. Of those, 72 (57.6%) lesions had MVI. Older age (P = 0.001) was significantly associated with MVI. Imaging features predictive of MVI included tumor size (P = 0.04), peritumoral arterial hyperenhancement (P = 0.002), peritumoral portal/delayed-phase washout (P = 0.02), indistinct lesion borders (P = 0.007), capsule disruption (P = 0.02), diffusion restriction (P = 0.001), mild-to-moderate T2 hyperintensity (P = 0.008), and mosaic architecture (P = 0.038). Conversely, LI-RADS category (P = 0.864) did not show significant associations with MVI. The optimal cutoff for lesion size was determined to be 38.5 mm.
**Conclusion:** This study highlights that certain clinical and imaging features are predictive of MVI in patients with HCC. These findings may aid in preoperative planning and improve treatment outcomes by enabling early identification of patients at higher risk of poor prognosis.

### SS 3.8

### Intraindividual comparison of promising prognostic features between CT and ECA-MRI in solitary BCLC 0/A HCC: implications for clinical outcomes

X. Jia, Y. Wang, B. Song, H. Jiang; *Chengdu/CN*

**Purpose:** MRI-based imaging features can predict prognoses for hepatocellular carcinoma (HCC) patients, but whether they can be effectively assessed and retain comparable prognostic value on CT remains unclear. This intraindividual study compared CT with ECA-MRI for detecting promising prognostic characteristics and predicting early (≤2 years) recurrence-free survival (E-RFS), overall survival (OS), and microvascular invasion (MVI).
**Material and Methods:** This single-center retrospective study (March 2014–March 2018) included consecutive patients with solitary BCLC 0/A HCC who underwent curative resection and had both preoperative contrast-enhanced CT and ECA-MRI. Three blinded radiologists independently evaluated 16 prognostic features in randomized manner with a ≥2-week washout. McNemar's test compared feature frequencies. Inter-modality agreement was calculated by intraclass correlation coefficient (ICC) or Cohen's kappa coefficient. Cox regression analyses were conducted for E-RFS and OS, while logistic regression analyses were conducted for MVI.
**Results:** Of 127 patients (median age 50 years; 106 men), 46.1% were MVI-positive. Early recurrence occurred in 33.7%, and 29.2% patients died during a median 66.0-month follow-up. CT and ECA-MRI indicated significant differences in frequencies of eight prognostic imaging features (i.e., corona enhancement; P < .001–.03). For E-RFS (HR, 2.10–3.94 on CT; 2.07–2.93 on MRI) and for OS (HR, 2.21–3.10 on CT; 1.95–5.84 on ECA-MRI). Tumor size demonstrated excellent inter-modality agreement (ICC, 0.95), while binary features ranged from poor to good (κ, 0.17–0.77). Tumor size predicted E-RFS on both modalities (P = .001–.02), whereas infiltrative appearance and blood products in mass predicted OS only on ECA-MRI.
**Conclusion:** ECA-MRI may hold better potential than CT for evaluating most promising prognostic features.

### SS 3.9

### Bone mineral density as a prognostic indicator of overall survival in patients with hepatocellular carcinoma and portal vein tumor thrombosis

L. Müller[1], R. Kloeckner[2], L. Heim[1], F. Hahn[1], F. Stoehr[1], T. Emrich[1], D. Graafen[1], J.-P. Grunz[3], D. Pinto dos Santos[4], A. Weinmann[1], F. Foerster[1], J. Mittler[1], J. Marquardt[2], T. Bäuerle[1], A. Mähringer-Kunz[1]; [1]*Mainz/DE*, [2]*Lübeck/DE*, [3]*Würzburg/DE*, [4]*Frankfurt/DE*

**Purpose:** Low bone mineral density (BMD) has emerged as a risk factor in hepatocellular carcinoma (HCC). This study explores the potential of BMD as a prognostic indicator in patients with HCC complicated by portal vein tumor thrombosis (PVTT), which is characterized by an exceptionally poor prognosis.
**Material and Methods:** This retrospective study included 462 patients with HCC and PVTT at our tertiary care center between January 2005 and December 2020. BMD was ascertained by measuring the CT attenuation at the mid-vertebral core of the first lumbar vertebra using the established cut-off of 160 HU. Analyses were performed at initial HCC diagnosis and PVTT onset. We analyzed the impact of BMD on median overall survival (OS) and conducted multivariate analyses with established survival predictors.
**Results:** The median BMD was 136 HU (interquartile range [IQR] 113–160 HU) at HCC diagnosis and 134 HU (IQR 109–159 HU) at PVTT diagnosis. At initial HCC diagnosis, the median OS was 10.4 months in patients with BMD ≥ 160 HU and 5.5 months in those with BMD < 160 HU (P < 0.001). Similarly, at PVTT diagnosis, the median OS was 8.5 months in individuals with higher BMD and 4.7 months in patients with lower BMD (P < 0.001). In multivariate analysis, BMD remained an independent prognostic factor alongside growth type and ALBI grade.
**Conclusion:** BMD is an independent prognostic marker of survival in patients with HCC and PVTT. Incorporating BMD into existing classification and scoring systems could enhance the accuracy of survival predictions and inform clinical decision-making processes.

### SS 3.10

### Prognostic factors for initially unresectable hepatocellular carcinoma after conversion and subsequent resection: a multi-center study

H. Jiang[1], B. Song[1], P. Wu[2], C. An[3]; [1]*Chengdu/CN*, [2]*Guangzhou/CN*, [3]*Beijing/CN*

**Purpose:** Assessing recurrence risk for patients with initially unresectable hepatocellular carcinoma (HCC) after conversion and subsequent curative-intent resection is critical for individualized management. This study aimed to investigate the risk factors for different postoperative recurrence patterns and survival outcomes.
**Material and Methods:** From April 2009 to September 2022, this multi-center retrospective study included 659 consecutive patients receiving conversion therapy and subsequent curative-intent resection for initially unresectable BCLC A/B HCC from seven tertiary-care hospitals. Central masked review for baseline CT and postoperative pathological features were performed by two independent radiologists and pathologists, respectively. Aggressive recurrence and advanced-stage recurrence were defined as first recurrence beyond the Milan criteria and at the BCLC C stage, respectively.
**Results:** All patients received transarterial chemoembolization, 292 (44.3%) combined with systemic therapy. Microvascular invasion (MVI) and the macrotrabecular-massive subtype were detected on postoperative pathology for 248 (37.6%) and 208 (31.6%) patients, respectively. Intratumoral artery on baseline CT (HRs, 1.18–37.42; p < 0.001–0.024) and objective response to conversion therapy per mRECIST (HRs, 0.29–0.60; p < 0.001–0.011), and MVI (HRs, 1.67–1.91; p = 0.001–0.027) were consistently associated with recurrence-free survival (RFS), aggressive RFS, advanced-stage RFS, and overall survival at the multivariable Cox analyses. Additionally, baseline serum des-gamma-carboxyprothrombin (>400 ng/ml; HR 1.16; p = 0.023) and α-fetoprotein (>400 ng/ml; HR, 1.50; p = 0.012) were independently associated with RFS and aggressive RFS, respectively, while the macrotrabecular-massive subtype was independently associated with advanced-stage RFS (HR, 1.70; p = 0.034).
**Conclusion:** Intratumoral artery, objective response to conversion therapy, and MVI were key predictive factors of different recurrence patterns and survival outcomes for patients receiving conversion therapy and subsequent curative-intent resection for initially unresectable BCLC A/B HCC.

11:00 – 12:30 E 105/106

### *Scientific Session SS 4*
### Crohn's disease

### *SS 4.1*
**MR enterography and intestinal ultrasound signs of Crohn's disease activity—are they interchangeable?**

M. Hameed[1], B. Adebusoye[1], S. Mallett[1], A. Higginson[2], S.A. Taylor[1], &. METRIC Trial Investigators[1]*; [1]London/UK, [2]Portsmouth/UK*

**Purpose:** MR enterography (MRE) and intestinal ultrasound (IUS) are often used interchangeably for Crohn's disease follow-up, although practice varies widely. This presents a challenge when comparing between modalities in the same patient. We aimed to establish the concordance between validated disease activity signs common to MRE and IUS.
**Material and Methods:** A retrospective review of the METRIC prospective cohort study database of 284 patients with newly diagnosed or suspected relapse Crohn's disease undergoing contemporaneous MRE and IUS was performed. Inter-modality agreement for disease activity variables defined in the METRIC protocol was assessed. Bland-Altman plots were constructed for continuous data and percentage agreement for categorical data.
**Results:** 158 patients, median age of 36 (range 17–68), 81/158 (51%) female, with terminal ileal disease were included. There was generally good inter-modality agreement for mural thickness (mean difference 1.19 mm, 95% limit of agreement [LOA] −3.95 to 6.33 mm) and length of disease, particularly <20 cm (mean difference 0.80 cm, 95% LOA −27.46 to 29.07 cm). Mural oedema (increased mural T2 signal) on MRE had the highest concordance with IUS submucosal layer thickening (91%) and lower concordance with IUS submucosal clarity (52%) or echogenicity (32%). Mesenteric oedema (increased perimural T2 signal) showed limited agreement with IUS mesenteric changes (32–48%). There was a 74% agreement for functional obstruction.
**Conclusion:** There is generally good agreement for the main disease activity criteria, particularly for mural thickness. MRE mural oedema has the highest concordance with IUS submucosal thickening. There is inter-modality disagreement between signs of mesenteric oedema. Further work on inter-modality concordance to inform imaging treatment follow-up pathways and training is needed.

### *SS 4.2*
**Exploring the role of large language models in structured reporting: a study on MR enterography impressions**

E. Cigdem Karatayli, E. Zengin, S. Evrimler*; Ankara/TR*

**Purpose:** Structured reporting (SR) aims to standardize radiological documentation to improve accuracy, reduce errors, and ensure adherence to guidelines. Large Language Models (LLMs), such as ChatGPT, might be helpful to create SR. Existing studies have promising results with concerns such as hallucinations of LLMs and data privacy. The adaptation of LLMs for structured reporting could reduce cognitive load on radiologists. This efficiency might translate to faster diagnoses, improved patient outcomes, and cost savings for healthcare systems. In this study, we planned to evaluate the success of LLMs in writing impression of statements in MRI enterography reports, as suggested by Guglielmo et al.
**Material and Methods:** Sixty synthetic MRI enterography reports, that mimic real radiology reports in structure and content, were created by an experienced abdominal radiologist and a fourth-year radiology resident. A reference impression was written by abdominal radiologist for each report. Three freely available LLMs—Claude 3.5, ChatGPT 4.0, and Perplexity—were instructed by a resident on writing impressions. Sample cases without impression of statements were presented to each LLM and asked to write the appropriate impression of statement. LLMs' impression of statements were compared with the reference statements.
**Results:** Impressions were categorized as inflammation, stricture, penetrating disease, perianal disease, and other complications (e.g., femoral head avascular necrosis). Claude 3.5 achieved the highest performance across categories, with kappa (k) and F1 scores of k = 1, F1 = 1; k = 1, F1 = 1; k = 0.96, F1 = 0.97; k = 0.91, F1 = 0.92; k = 0.92, F1 = 0.93; with $p < 0.05$. ChatGPT ranked second, followed by Perplexity.
**Conclusion:** LLMs represent a transformative opportunity for SR in radiology by accuracy. However, realizing their potential hinges on overcoming technical, regulatory, and ethical barriers.

### *SS 4.3*
**Predictive value or inflammatory burden and severity of inflammation measured by MR enterography to assess therapeutic response in small bowel Crohn's disease**

J.M. Saidman, A. Fernàndez-Clotet, J. Puig, M. Comas, I. Ordás, J. Rimola*; Barcelona/ES*

**Purpose:** Identifying predictors of therapeutic response is the cornerstone of personalised medicine. We aim to identify findings on MR enterography (MRE) 46 weeks after initiating biological treatment in small bowel Crohn's disease (SBCD) that predict adverse long-term outcomes.
**Material and Methods:** Post-hoc analysis of a prospective longitudinal single-centre study included patients with SBCD requiring biological treatment. MRE data were obtained at baseline and at week 46. Patients were followed two years afterwards. A logistic regression analysis was performed to assess the prognostic value of different radiological findings, including length of inflammation (inflammatory burden) and severity of lesions measured by sMaRIA, for various predefined adverse clinical outcomes.
**Results:** Among the 65 patients included, 39 (60%) had ≥1 adverse outcome during follow-up: 29 (44.6%) had clinical recurrence; 20 (30.76%) required surgery, and 12 (18.46%) hospitalization. At week 46, healing of severe lesions (defined as sMaRIA < 2) was found in 24/65 (36.92%). Reduction of length of inflammation of any severity or of severe inflammation was not associated with adverse clinical outcomes ($p > 0.055$ and $p > 0.059$, respectively). In the multivariate analysis, persistence of severe lesions at week 46 predicts higher risk of surgery (OR = 1.71 [95%CI 1.18–2.58] p = 0.007) and clinical relapse (OR = 1.93 [95%CI 1.31–3.06]; p = 0.002), whereas improvement of radiological severity (reduction of sMaRIA of at least 1.5 units) predicts the absence of adverse outcomes (OR = 0.46 [95%CI 0.27–0.73]; p = 0.002).
**Conclusion:** Improvement in radiological severity in SBCD measured by sMaRIA has a positive impact on clinically relevant disease outcomes, whereas changes in the reduction of inflammatory burden (disease length) do not show such an association.

### *SS 4.4*
**MRI assessment of body composition for prediction of therapeutic response to anti-TNF[illegible] agents in patients with Crohn's disease**

N.S. Sakai[1], A. Plumb[2], N. Ahmed[1], K. Chowdhury[1], Y. Kilic[1], M. Hameed[1], A. Patel[3], A. Bhagwanani[4], E. Helbren[5], R. Hyland[6], G. Bhatnagar[1], H. Sidhu[1], H. Lambie[6], J.M. Franklin[7], M. Mohsin[6], E. Thomson[6], D. Boone[1], D. Tolan[6], S. Rahman[8], N. Ding[9], A. Menys[1], S. Halligan[1], S.A. Taylor[1]*; [1]London/UK, [2]Esher/UK, [3]Edinburgh/UK, [4]Slough/UK, [5]Hull/UK, [6]Leeds/UK, [7]Bournemouth/UK, [8]Epsom/UK, [9]Melbourne/AU*

**Purpose:** Altered body fat and muscle in Crohn's disease (CD) has been linked to adverse disease course and outcomes. Accurate prediction of treatment response or remission (RoR) of small bowel CD (SBCD) to biologic therapy remains challenging. We aimed to establish the prognostic value of body composition parameters measured using MR enterography (MRE) for RoR at one year in patients with SBCD commencing biologic therapy.
**Material and Methods:** Participants were identified from those recruited to a prospective, multicentre study investigating the predictive ability of motility MRI for one year RoR in patients starting biologic therapy for active SBCD (MOTILITY trial). Myopenia, skeletal muscle:fat and visceral:subcutaneous fat were measured from baseline MRE. RoR at one year was judged using a composite of clinical and morphological MRE parameters. We compared the likelihood of RoR in patients with and without myopenia, and low skeletal muscle:fat, using logistic regression models.
**Results:** Ninety-six participants were included (mean age 38.2 years; 40 [42%] female). There were 34 (35%) responders. There was no significant difference in RoR at one year between those patients with and without skeletal muscle myopenia (OR: 0.85, 95% CI: 0.27, 2.66, p-value: 0.78), and those with or without low skeletal muscle:fat (OR: 0.71, 95% CI: 0.19, 2.71, p-value: 0.62).
**Conclusion:** Body composition parameters demonstrated no value for predicting therapeutic RoR in patients commencing biologic therapy for SBCD.

### SS 4.5

### Deciphering gut microbiota and metabolites interactions with magnetic resonance enterography phenotypes of bowel damage in Crohn's disease: toward developing a multidimensional diagnostic model

J. Meng, M. Wang; *Zhengzhou/CN*

**Purpose:** Crohn's disease tends to result in bowel damage (BD) which often requires surgery. BD is closely related to the changes of intestinal microbiota and metabolites, but no detail has been reported yet. As a preferred imaging method, magnetic resonance enterography (MRE) provides indispensable value for the BD extent evaluation. A multi-omics approach incorporating genomics, metabolomics and MRE was used for exploration of the potential pathogenesis in BD, and prediction models were established in predicting BD.
**Material and Methods:** 230 patients including BD and no bowel damage (NBD) by Lémann Index and 30 healthy controls were enrolled. The feces and serum from the subjects were collected. 16S rRNA sequencing, ultra-high performance liquid chromatogen-mass spectrometry and MRE including T2-weighted imaging (T2WI), contrast-enhanced and diffusion-weighted imaging (DWI) were performed. The association network, random forest classifier and receiver operating characteristic curve were used for analysis.
**Results:** MRE signs including penetration, intestinal enhancement extent and perienteric T2WI signal etc. were significantly different between BD and NBD. The potential mechanism of BD may involve intestinal microbiota Blautia, fecal arachidonic acid, serum ceramide and MRE signs including penetration and perienteric T2WI signal. The AUCs of models with microbiota, metabolites and MRE separately and the combined model were 0.588–0.708 ($P < 0.05$).
**Conclusion:** Microbiota Blautia, fecal arachidonic acid and serum ceramide may participate in the mechanism of BD through lipids and glucose metabolism disorders, oxidative stress and inflammation, and are characterized by MRE signs including penetration and perienteric T2WI signals. The efficacy of the combined model is superior to that of the model with microbiota, metabolites, and MRE signs separately in predicting BD.

### SS 4.6

### Inter- and intra-observer variability of software quantified bowel motility measurements of Crohn's disease: findings from the MOTILITY trial

M. Hameed[1], A.A. Plumb[1], K. Chowdhury[1], N. Ahmed[1], S. Rahman[2], G. Bhatnagar[3], E. Thomson[4], M. Mohsin[4], J. Holmes[1], S. Halligan[1], S.A. Taylor[1], &. MOTILITY Trial Investigators[1]; *[1]London/UK, [2]Epsom/UK, [3]Frimley/UK, [4]Leeds/UK*

**Purpose:** Motility MRI (mMRI) is a potential marker of disease activity of small bowel Crohn's disease (SBCD) but there is limited data on its reproducibility. We assessed inter- and intra-observer agreement as part of a prospective multicentre trial investigating whether mMRI can predict longer term response to biologic therapy in active, non-stricturing SBCD.
**Material and Methods:** 297 segmental small bowel motility scores from 104 SBCD patients (mean age 38.9 years, 43 female) recruited to the MOTILITY trial were measured independently by two radiologists experienced in mMRI, using GIQuant software (Motilent UK). Twenty-six datasets were re-read by both radiologists to test intra-observer variability after a washout period of >6 weeks. Five gastrointestinal radiologists inexperienced in mMRI derived 66 segmental motility scores from the same 30 randomly selected patients. Agreement was quantified using the intra-class correlation coefficient (ICC).
**Results:** There was good to excellent agreement for mMRI-derived segmental small bowel motility measurements for both mMRI experienced and inexperienced radiologists (ICC 0.74 [95% CI: 0.68, 0.80] and 0.92 [95% CI: 0.89, 0.95], respectively). Agreement remained excellent combining the experienced trial MRI reader measurements with those of the five inexperienced radiologists (ICC 0.93 [95% CI: 0.90, 0.95]. Intra-observer agreement for the two mMRI experienced radiologists was excellent (0.83 [95% CI: 0.61–0.92] and 0.83 [95% CI: 0.62, 0.92]).
**Conclusion:** There is good to excellent inter- and intra-observer agreement for mMRI measurements of segmental small bowel motility for both experienced and inexperienced radiologists. Study findings support the continued clinical translation of mMRI as a reproducible biomarker of disease activity and treatment response in Crohn's disease.

### SS 4.7

### Impact of objective motility MRI scoring of Ileal Crohn's disease on gastroenterologists' treatment assessment: the CONTEXT trial

A. Menys[1], S.A. Taylor[1], I. Naim[1], H.E. Fitzke[1], J. Barber[1], K. Patel[1], N. Patel[1], A. Ocholi[1], D. Khodabakhshi[1], S. Baillie[1], N. Onyeador[1], V. Jennings[2], S. Vuyyuru[2], C. Clarke[2], R. Munyal[2], G. Bhatnagar[3], T. Shepherd[3], S. Upponi[4], J. De la Revilla Negro[4], K. Sethi-Arora[5], A. Kuzhiyanjal[5], J. Limdi[5], A. Win[5], P. Ghodekar[5], A. Siddiuqui[5], D. Tolan[6], H. Lambie[6], W. Weston[1], P. Harrow[1], J. Yeo[1], M. Hameed[1], G. Moran[2]; *[1]London/UK, [2]Nottingham/UK, [3]Frimley/UK, [4]Cambridge/UK, [5]Manchester/UK, [6]Leeds/UK*

**Purpose:** MRI-derived small bowel motility in Crohn's disease (CD) is a validated biomarker of disease activity, but its impact on clinical decision-making remains unknown. We evaluated this in a real-world setting.
**Material and Methods:** Ten practicing gastroenterologists across 6 sites identified CD patients with baseline and 1-y treatment follow-up MR enterography. For both time points, a motility score (GIQuant, Motilent) was derived by a radiologist using a region of interest placed in the most active small bowel segment. Using available clinical information including imaging but excluding GIQuant scores, gastroenterologists graded treatment response on a 6-point scale: much worse, somewhat worse, slightly worse, or much better, together with their opinion on 12-month prognosis and surgical likelihood. GIQuant scores were then revealed, and gastroenterologists updated clinical impressions guided by the quantitative change in SB motility scores.
**Results:** 170 ileal or ileocolonic CD patients (61% male, mean age 36 years) were included. Most (84%) patients were on biologics. Revealing GIQuant scores led to changes in clinical opinion in 74 (44%) for disease response status, 65 (38%) for 12-month prognosis, and 44 (26%) for surgical referral likelihood. Most (76%) adjustments for disease response status were 1-step changes (e.g., "slightly better to "somewhat better"), but 24% were more substantial 2- or 3-step changes (e.g., from "slightly worse" to "somewhat better). There was a reversal in clinical impression, e.g. better to worse in 36 cases (21%).
**Conclusion:** Objective motility scoring impacts clinical impression of response in the majority of patients and reverses this impression in about a fifth of patients.

### SS 4.8

### Patient's attitudes to magnetic resonance imaging in perianal fistulizing Crohn's disease: a global survey

E. Anand[1], J. Devi[2], A. Antoniou[3], S. Joshi[1], A. Hart[1], P. Lung[1], P. Tozer[1], D. Ballard[2], P. Deepak[2], J. Stoker[4]; *[1]London/UK, [2]St. Louis, MO/US, [3]Oxford/UK, [4]Amsterdam/NL*

**Purpose:** There is limited patient involvement in radiological research for perianal fistulizing Crohn's disease (pfCD), despite MRI's critical role in diagnosis and management. Patient and public involvement is essential for aligning research with patient priorities. This study aimed to gather patient perspectives on the use of MRI in pfCD.
**Material and Methods:** A mixed-methods approach was used, following the Guidance for Reporting Involvement of Patients and the Public (GRIPP2) guidelines. An online survey, co-developed with a patient representative, included open and closed questions on MRI experiences, advantages, challenges, and the potential for AI-generated reports. This was followed by a virtual session for further exploration of patient views. Thematic analysis was conducted on the data.
**Results:** Forty-seven patients with Crohn's disease (37 with pfCD) from six countries participated, with 28/37 (76%) completing the survey. Key themes included patient expectations for MRI, preferences for scan intervals and report content. Most (93%) wanted MRI reports to compare with previous scans, highlighting fistula changes and new abscesses. A majority (57%) preferred MRI scans annually when well, and more frequently after surgery (64.3% preferred scans at 3 months). Emotional relief was associated with MRI improvements, though access to services and report clarity remained challenges. Interest in AI-generated patient-friendly summaries was expressed if clearly explained and validated by professionals.
**Conclusion:** This is the first study exploring patient views on MRI use in pfCD, emphasizing the need for patient-centred MRI reporting and clearer communication. Future work should enhance patient access and validate AI-generated patient-friendly summaries of MRI reports.

### SS 4.9

### Defining radiological healing in perianal fistulising Crohn's disease: a global modified Delphi consensus

E. Anand[1], J. Devi[2], S. Joshi[1], A. Antoniou[3], A. Hart[1], P. Lung[1], P. Tozer[1], D. Ballard[2], P. Deepak[2], J. Stoker[4]; *[1]London/UK, [2]St. Louis, MO/US, [3]Oxford/UK, [4]Amsterdam/NL*

**Purpose:** Magnetic resonance imaging (MRI) is considered the gold standard for assessing healing in perianal fistulizing Crohn's disease (pfCD), but a lack of consistent radiological definitions complicates clinical interpretation and trial design. This study aimed to establish an international consensus on defining radiological healing in pfCD.

**Material and Methods:** In Phase 1, a systematic review of the literature identified MRI-based variables and indices used to define radiological healing in pfCD. The quality of these indices was assessed using the COnsensus-based Standards for the selection of health Measurement INstruments (COSMIN) framework, following PRISMA-COSMIN guidelines. Phase 2 involved a two-round online Delphi consensus survey of international experts in pfCD, followed by a stakeholder meeting. Consensus was defined by an agreement threshold of over 80%, reported in line with Accurate Consensus Reporting Document (ACCORD) guidelines.

**Results:** Eighty-four experts from 16 countries participated, achieving a strong consensus (95%) on the definition of a radiologically healed fistula on MRI: absence of T2-weighted hyperintensity, a completely fibrotic fistula tract, and no contrast enhancement on post-contrast T1-weighted images (where used). Radiological improvement of a fistula was defined by 80% consensus as a predominantly fibrous fistula tract, or an unequivocal reduction in any of the following: T2-weighted hyperintensity, fistula diameter, length, abscess size, or hyperintensity on contrast-enhanced T1-weighted images (where used).

**Conclusion:** This consensus represents a significant step toward standardizing radiological endpoints in pfCD. Adopting this definition will improve the consistency of pfCD evaluations in both clinical practice and research. Future studies will validate this definition and explore its correlation with long-term clinical outcomes.

### SS 4.10

### The PTRACK-generated normalised average T2 signal is an accurate and sensitive measure of perianal Crohn's disease activity

I. Naim[1], A. Menys[1], G. Bhatnagar[2], C. Greer[2], L. Lindsay[1], L. Carter[1], G.W. Moran[3]; *[1]London/UK, [2]Frimley/UK, [3]Nottingham/UK*

**Purpose:** MRI is the gold standard for diagnosing and monitoring perianal Crohn's disease (pCD). Fistula T2 signal intensity and volume are key predictors of treatment response. We developed pTRACK, a tool enabling radiologists to measure fistula volume, normalised T2 signal intensity, and anatomical relationships, generating 3D models of pCD within minutes. This study evaluates pTRACK metrics against MAGNIFI-CD and their predictive value for clinical outcomes.

**Material and Methods:** We retrospectively included consecutive adult patients (>18 years) with pCD who underwent pelvic MRI and clinical assessment. Two independent, blinded abdominal radiologists scored MAGNIFI-CD and pTRACK metrics on axial T2-STIR TSE sequences. Clinical outcomes (remission, response, non-response) were scored using the fistula drainage assessment (FDA). Correlations, unpaired two-tailed t-tests, and leave-one-patient-out cross-validation (LOPOCV) was used to compare MRI metrics and predict FDA categories. T2 signal intensity was normalised using a region of interest in adjacent pelvic muscles.

**Results:** 36 patients (CRP = 12.9 ± 22 mg/L) with 77 MRI scans were included. 58.4% were non-responders, 14.3% responders, and 27.3% in remission. Significant differences were observed between remission and active groups: normalised T2 signal (1.65 vs. 2.98, $p < 0.001$), fistula volume (1.58 vs. 4.55 mL, $p = 0.03$), and MAGNIFI-CD (5.86 vs. 12.41, $p < 0.001$). Fistula volume and T2 signal correlated moderately with MAGNIFI-CD ($r = 0.72$ and $r = 0.49$, $p < 0.001$, respectively). LOPOCV AUCs were 0.77, sensitivity = 87% for T2 signal; 0.74, sensitivity = 95% for fistula volumes; and 0.84, sensitivity = 77% for MAGNIFI-CD.

**Conclusion:** pTRACK's normalised T2 signal demonstrated comparable accuracy to MAGNIFI-CD, with higher sensitivity for predicting pCD activity and distinguishing active from non-active disease. These findings underscore pTRACK's potential utility, warranting prospective validation in cohorts with more severe diseases.

**14:30 – 16:00** **E 103/104**

*Scientific Session SS 5*

### Pancreatic ductal adenocarcinoma

### SS 5.1

### The PANORAMA study results: pancreatic cancer diagnosis—radiologists meet AI

N. Alves, M. Schuurmans, P. Vendittelli, G. Litjens, J. Hermans, H. Huisman; *Nijmegen/NL*

**Purpose:** The PANORAMA study evaluates radiologists and artificial intelligence (AI) in detecting pancreatic ductal adenocarcinoma (PDAC) using contrast-enhanced computed tomography (CECT).

**Material and Methods:** This retrospective study includes 3338 CECT abdominal scans of patients without prior history of treatment or positive histopathology findings of PDAC acquired between 2006 and 2021 from 5 centres (Netherlands, Norway, and Sweden). 2238 cases (676 PDAC) were publicly released to develop and train AI algorithms, and 100 and 1000 cases were sequestered for AI tuning and testing, respectively. The test set comprises data from two external centres not present in the other cohorts. A subset of 400 testing cases is used for the PANORAMA reader study. In total, 57 radiologists registered. Both AI and radiologists indicate PDAC likelihood and localization of lesions. Additionally, radiologists provide a PANC-RADS score for its urgency for expert referral. AI is developed by teams from all over the world and submitted to the same evaluation metrics using the Grand Challenge platform. Patient-level performance is assessed using the area under the receiver operating characteristic curve (AUROC), while lesion localization performance is assessed using average precision (AP).

**Results:** The PANORAMA reader study and Grand Challenge results will be presented at ESGAR 2025. Currently, 57 radiologists (39 centers, 13 countries, 2–30 years of experience, median: 9 years) participate in the study. The baseline AI algorithm provided by the challenge organization team (nnU-Net with cross-entropy loss) achieves 0.9776 AUROC and 0.7226 AP in the tuning set.

**Conclusion:** Transparently benchmarked, expert AI can enable opportunistic screening to start catching PDAC at earlier stages and improve patient survival.

### SS 5.2

### Spectral imaging for pancreatic cancer: elevating diagnostic confidence with PCD-CT

A.I. Vigh, Z. Somogyiné Nagy, B. Budai, E. Tóth, Á. Szücs, P. Kaposi, A. Szijártó, P. Maurovich-Horvat, I. Dudás; *Budapest/HU*

**Purpose:** This study aimed to assess whether advanced photon-counting detector CT (PCD-CT) imaging techniques, including virtual monoenergetic imaging (VMI) and iodine map reconstructions, can help radiologists in the diagnosis of pancreatic adenocarcinomas in challenging cases.

**Material and Methods:** Patients who underwent PCD-CT for suspected pancreatic adenocarcinoma between January 2022 and January 2024 were retrospectively identified. Twenty-nine challenging cases, where radiologists lacked diagnostic confidence, were selected based on radiology reports. Diagnoses were confirmed as malignant (n = 24) by biopsy/histology or non-tumorous (n = 5) through further imaging/biopsy assessment. For this study, two radiologists, blinded to patient records, reviewed pancreatic-phase spectral CT scans using 70 keV VMI, 40 keV VMI, and iodine maps sequentially. Tumor detection, diagnostic confidence (5-point scale), vascular status, and T-stage were evaluated. Differences were analyzed using the Wilcoxon signed-rank test.

**Results:** One tumor was missed by the junior radiologist. Among the five non-tumorous cases, 40 keV VMIs helped to exclude one false-positive diagnosis from the 70 keV VMIs. Vascular assessments (tumor contact, stenosis/thrombosis) changed in 7/24 and 4/24 cases using 40 keV VMIs and 9/24 and 5/24 using iodine maps. T-staging changed in 4/24 and 5/24 cases for 40 keV VMIs and iodine maps, respectively. Diagnostic confidence improved from 3.38 ± 0.73 (70 keV VMI) to 4.00 ± 1.00 (40 keV VMI, $p < 0.001$) and 3.93 ± 0.88 (iodine maps, $p = 0.001$). The radiologists found 40 keV VMIs and iodine maps helpful in >90% and >75% of the cases, respectively.

**Conclusion:** 40 keV VMIs and iodine maps significantly enhance diagnostic confidence in detecting pancreatic adenocarcinomas, especially in challenging cases.

### SS 5.3

### Diagnostic performance of CT in the assessment of perineural infiltration in borderline resectable pancreatic head adenocarcinoma

G. Giacomelli[1], M. Marchegiani[2], A. Zannotti[3], A. Borgheresi[1], A. Agostini[1], A. Benedetti Cacciaguerra[1], M. Vivarelli[1], A. Giovagnoni[1]*; [1]Ancona/IT, [2]Passo di Treia/IT, [3]Cupramontana/IT*

**Purpose:** To assess the diagnostic performance of CT findings associated with perineural infiltration in patients with borderline resectable adenocarcinoma of the pancreatic head (PHDAC).

**Material and Methods:** We included patients who underwent surgical resection (January 2017–January 2019) for borderline resectable PHDAC who had available preoperative multiphasic contrast-enhanced CT (within 1 month; LightSpeed VCT, GE, 120 kV, variable mA, ASIR 60%, thickness/spacing: 2.5/2.5 mm, Iopamidol 370; 1.5 ml/kg @3–5 ml/s) with complete pathological report available. Patients undergoing neoadjuvant treatment were excluded. CT images were reviewed by two expert radiologists in consensus and blinded to pathology. The presence of a CT abnormality in peripancreatic fat (haziness, linear or reticular lesions, soft tissue, or mixed), the continuity with the primary tumor, the relation with the vascular landmarks (pancreaticoduodenal arteries) of the pancreatic plexus (PNP), and the presence of enlarged or dysmorphic celiac/mesenteric ganglia were evaluated. The statistical analysis was performed with receiver operating characteristic (ROC) analysis and logistic regression.

**Results:** 41 patients (21M, 20F, median age 70 y.o.) were included. At pathology, the infiltration of the PNP was detected in 36/42 cases (86%). The presence of any CT finding in continuity with the primary tumor along the vascular landmarks of the PNP was significantly correlated with perineural infiltration at ROC analysis (AUC: 0.874; SE 94.74%, SP 80.00%) and was an independent predictor of perineural invasion (logistic regression $p < 0.0001$, $R2 = 0.610$).

**Conclusion:** The assessment of perineural infiltration in borderline resectable PHDAC is feasible in CT, with high sensitivity and good specificity.

### SS 5.4

### Key factors for predicting vascular invasion and recurrence of conversion surgery after chemotherapy in pancreatic cancer: integrating preoperative CT and clinicopathological data

S. Hwang, J. Kim, J. Park, R. Ryu*; Seoul/KR*

**Purpose:** To investigate the predictors of vascular invasion and recurrence-free survival (RFS) in patients with pancreatic cancer undergoing conversion surgery after chemotherapy using computed tomography (CT) and histopathological data.

**Material and Methods:** We retrospectively analyzed 98 patients with pancreatic cancer showing vascular encasement on CT who underwent chemotherapy after conversion surgery. Two reviewers evaluated the baseline and preoperative CT scans to assess resectability. Clinical and histopathological factors, such as T- and N-stage, tumor differentiation, presence of vascular invasion, and resection status, were assessed. RFS was analyzed using the Kaplan–Meier method. The predictors were identified using Cox proportional hazards and logistic regression models. A 3-point scale was used to evaluate arterial and venous involvement.

**Results:** Of the 98 patients, 32 (32.7%) had pathological vascular invasion. Multivariate analysis showed T stage (odds ratio [OR], 10.3; $p = 0.040$), microvessel invasion (OR, 8.5; $p = 0.002$), perineural invasion (OR, 12.4; $p = 0.008$), and changes in tumor size (OR, 3.1; $p = 0.022$) as significant predictors of vascular invasion. Total A and V scores were strongly correlated with vascular invasion ($p < 0.001$; areas under the receiver operating characteristic curve were 0.924 and 0.803, respectively). The median RFS was 21 months, which was influenced by lymph node involvement at baseline and preoperative CT (hazard ratio [HR] 2.3; $p = 0.009$ and HR 2.6; $p = 0.013$, respectively), tumor differentiation (HR 0.3; $p = 0.002$), microvessel invasion (HR 2.2; $p = 0.012$), and elevated CA 19-9 levels (HR 2.0; $p = 0.039$).

**Conclusion:** The changes in tumor size on CT, T stage, microvessel invasion, and perineural invasion are important for predicting vascular invasion. Additionally, CT-based scoring systems can accurately predict vascular invasion.

### SS 5.5

### Development of a CT radiomics model for assessing perineural and perivascular infiltration of adenocarcinoma of the pancreatic head

M. Marchegiani, G. Giacomelli, A. Borgheresi, A. Zannotti, F. Mariotti, A. Benedetti Cacciaguerra, F. Mocchegiani, A. Agostini, A. Giovagnoni*; Ancona/IT*

**Purpose:** To develop a CT radiomics model for detecting perineural infiltration (PNI) and perivascular infiltration (PVI) in patients with adenocarcinoma of pancreatic head (PHDAC).

**Material and Methods:** Patients who underwent surgical resection for PHDAC (January 2017–January 2019), with preoperative CT (within 1 month; Light-Speed VCT, GE, 120 kV, variable mA; Iopamidol 370; 1.5 ml/kg @3–5 ml/s) and complete pathological report, were included. Patients undergoing neoadjuvant treatment were excluded. On the venous phase on the axial section, two readers in consensus placed two circular region-of-interest (diameter 8 mm), the first including part of the tumor and of the peripancreatic fat and the second subtracting the tumor. The radiomics features were extracted from normal and filtered images (LoG, Sqrt, Sqr, Log, Wavelet, Exp), with validated software (PyRadiomics on Syngo.via Frontier, Siemens) by applying a bin-width of 25 (ImageJ, NIH), a resampling of 1x1x1, with univariate, multivariate analysis and Random Forest (RF) Machine Learning (trained and tested with k-cross validation endpoint: PNI and PVI).

**Results:** 39 patients were included (median age 69 years): 32/39 had PNI 29/39 had PVI. The multivariate analysis identified 7 features belonging to the glszm, first-order, and gldm classes (wavelet and Sqr filter) and 10 features from the ngtdm, glcm, glszm, gldm, glrlm, and first-order classes (wavelet filter) as independent predictors ($p < 0.001$) of PNI and PVI. The RF models achieved AUCs of 0.82 and 0.76 with accuracies of 0.89 and 0.72 for PNI and PVI, respectively.

**Conclusion:** Radiomics analysis is feasible and effective in the preoperative detection of PNI and PVI in PHDAC.

### SS 5.6

### Inter-observer agreement of pancreatic tumor size measurement before and after neoadjuvant therapy: is MRI as reproducible as CT?

A. Licha, C. Touloupas, A. Delpla, A. Pouvelle, M. Zins*; Paris/FR*

**Purpose:** To assess inter-observer agreement of pancreatic ductal adenocarcinoma (PDAC) tumor size measurement on CT and MRI, before and after neoadjuvant therapy (NAT).

**Material and Methods:** We reviewed all patients with a histological diagnosis of PDAC at Paris Saint-Joseph Hospital, between 2010 and 2022 and who underwent CT and MRI, both before and after NAT. Three independent radiologists anonymously evaluated the large axial tumor axis on 2 CT acquisitions and 6 MRI sequences. Inter-observer agreement was assessed by intra-class correlation coefficients (ICCs) and by LOAM graphs (Bland & Altman extension for multiple observers).

**Results:** The final population consisted of 50 patients. On CT exams, inter-observer agreement was excellent before NAT (ICC of 0.83 [0.73; 0.90] at arterial phase and 0.84 [0.74; 0.90] at portal-venous phase) and decreased but remained good after NAT (ICC of 0.66 [0.52; 0.78] at arterial phase and 0.65 [0.51; 0.77] at portal-venous phase). On MRI exams, inter-observer agreement was moderate to good before NAT (best sequence being T1 at arterial phase with ICC of 0.67 [0.53; 0.79]) and decreased becoming moderate for all sequences after NAT (best sequence being T1 at late phase with ICC of 0.55 [0.37; 0.71]).

**Conclusion:** Inter-observer agreement of PDAC tumor size measurement is better on CT than on MRI, and decreases between pre-NAT and post-NAT imaging, both on CT and MRI. These results encourage us to keep on performing technically perfect CT scans, without questioning the need for preoperative hepato-pancreatic MRI in non-metastatic patients.

### SS 5.7

### The value of routine liver MRI in staging of potentially operable pancreatic cancer

R. Ghaffar; *Birmingham/UK*

**Purpose:** To assess the value of routine liver MRI in staging of operable pancreatic cancer.
**Material and Methods:** In our practice, CT was the main diagnostic modality for staging pancreatic cancer. The role of liver MRI was to clarify indeterminate findings on CT. A few years ago, routine MRI liver has been added to the pathway of fast-track referrals/potentially operable cancer pancreas patients, based on the published data of the MRI liver added value. However, we need to evaluate our practice/ set up local standard based on local evidence. This study is one of the largest single-centre studies in this topic. We reviewed all fast-track referrals from June 2019 to July 2023, totaling 371 patients. Only patients who underwent liver MRI with contrast and DWI have been included. Patients with benign pathology, alternative malignancy or metastasis on CT have been excluded. The total study population was 201 patients.
**Results:** MRI diagnosed liver metastasis when CT was negative in 7/201 patients (3.5%) which is significantly lower than the published figures. A pictorial review of those cases has been done for learning purposes. MRI clarified approximately 60% of the indeterminate lesions on CT (5% of the study population). MRI caused confusion/indeterminate in 5/201 patients (2.5% of the total study population) when CT was negative. MRI missed liver metastasis in 3/201 patients (1.5%) which was found intraoperatively.
**Conclusion:** An economic module is required to assess the value/cost effectiveness of adding liver MRI to the staging pathway of all potentially operable pancreatic cancers in view of our results, as opposed to using it for problem solving.

### SS 5.8

### The role of sarcopenia on clinical outcomes in patients with pancreatic ductal adenocarcinoma undergoing neoadjuvant chemotherapy followed by curative-intent resection

S. Malakzadeh[1], R. Sartoris[2], A. Sabounchi[3], J. Grégory[2], M. Dioguardi Burgio[2], A. Sauvanet[2], M. Ronot[2]; [1]*Sion/CH*, [2]*Clichy/FR*, [3]*Tehran/IR*

**Purpose:** This study aims to investigate the role of sarcopenia in predicting clinical outcomes in pancreatic ductal adenocarcinoma (PDAC) patients treated with neoadjuvant therapy (NAT) and subsequent curative-intent resection. The analysis focuses on both static and dynamic changes in muscle quality and quantity during treatment.
**Material and Methods:** Retrospective cohort of 143 PDAC patients (63.5 ± 9.6 years, 51% male) who underwent NAT followed by pancreatoduodenectomy between 2015 and 2020 were studied. Sarcopenia-related metrics were assessed at the L3 vertebral level on contrast-enhanced CT, using pre- and post-treatment psoas muscle index (PMI), psoas muscle thickness ratio (PMTR), and intramuscular fat content (IMFC). Overall (OS) and recurrence-free survival (RFS) were compared with log-rank tests and Cox proportional hazard models.
**Results:** The mean OS and RFS were 29.5 ± 19.0 months and 22.9 ± 19.5 months, respectively. Patients without sarcopenia according to pre-treatment PMI exhibited a 12-month OS benefit compared to those with PMI-defined sarcopenia ($p < 0.01$), and pre-treatment PMI was an independent predictor of OS (HR: 3.33, $p = 0.02$) and RFS (HR: 1.79, $p = 0.03$) in multivariable analyses. Changes in PMTR during NAT were associated with OS (HR: 0.83, $p = 0.04$), with patients showing stable PMTR values experiencing a mean OS advantage of 10 months compared to those with declining values (34.2 ± 18.3 vs. 24.1 ± 16.9 months, $p < 0.01$). IMFC was not significantly associated with clinical outcomes ($p > 0.05$).
**Conclusion:** Pre-treatment PMI and PMTR changes during NAT are strong predictors of survival outcomes in PDAC patients. These metrics could help identify high-risk individuals who may benefit from targeted nutritional and physical interventions during NAT, ultimately improving treatment outcomes.

### SS 5.9

### Early pancreatic ductal adenocarcinoma viscoelastic changes after neoadjuvant chemotherapy

S. Almutawakel, U. Pelzer, I. Sack, S.R. Marticorena Garcia; *Berlin/DE*

**Purpose:** The purpose of this study is to investigate viscoelastic changes of primarily non-resectable pancreatic ductal adenocarcinoma (PDAC) after treatment with neoadjuvant/palliative chemotherapy by using multifrequency magnetic resonance elastography (MRE).
**Material and Methods:** In this prospective study, 17 participants (7 female, median age 61 [IQR 56–74] years) with histological confirmed PDAC (n = 10 with locally advanced pancreatic cancer [LAPC] and n = 7 with metastasis [MET]) were examined by multifrequency MRE at 3T using tomoelastography post-processing at frequencies of 30, 40, 50 and 60 Hz providing shear wave speed (SWS) in m/s as surrogate of soft tissue stiffness. All participants were examined at baseline and after 3 months completing the sixth treatment cycle of neoadjuvant/palliative chemotherapy (FOLFIRINOX [n = 14] or GEM-Pac). MRE (response ≥10% SWS-change) was compared to maximum PDAC diameter based on clinical routine computed tomography (CT) corresponding to RECIST 1.1. Wilcoxon-test was used for group (PDAC) and subgroup analysis (LAPC/MET).
**Results:** The median SWS in PDAC decreased from 2.20 (IQR 2.00–2.67) m/s to 2.17 (1.69–2.54) m/s, $p = 0.03$, and from 2.18 (1.93–2.32) m/s to 1.85 (1.58–2.19) m/s in LAPC, $p = 0.01$. The median PDAC diameter decreased from 4.20 (3.0–5.2) cm to 3.6 (2.9–5.0) cm, $p = 0.01$, and from 3.9 (2.7–4.9) cm to 3.2 (2.6-–4.3) cm in LAPC, $p = 0.02$. No differences were observed in MET (SWS/diameter, p = 0.8/0.3). Partial response, stable disease and progressive disease were observed by MRE (n = 11/3/3) and CT/RECIST 1.1 (n = 6/10/1).
**Conclusion:** Treatment effects after chemotherapy might be reflected earlier in PDAC viscoelasticity changes compared to RECIST 1.1 alone.

### SS 5.10

### Quantitative 3t MR and MR elastography assessment of pancreatic texture and its correlation with surgeon's palpation and histopathology

P. Taprania, B. Sureka, T. Yadav, V. Varshney, S.C. Soni, P. Elhence, J.R. Vishnoi, P.S. Khera; *Jodhpur/IN*

**Purpose:** To study various MR imaging features that quantifies texture (soft vs firm) of pancreas and its correlation with surgeon's palpation method and histopathology in predicting the post-operative complications related to pancreatic texture.
**Material and Methods:** This prospective single-centre study included a total of 42 cases that underwent Whipple's surgery between July 2022 and January 2024, out of which 30 were operated. Various MR parameters and elastography values were studied and correlated with intra-operative palpation and histopathology of the resected stump to predict the texture (soft vs firm) of pancreas.
**Results:** We found that out of 30 cases, intra-operatively 21 patients had soft pancreatic texture and 9 had firm pancreatic texture. On MR quantitative analysis, duct diameter less than 2.6 ($p = 0.014$, sensitivity 88.9%, specificity 61.9%), MR Elastography less than 2.65kPa ($p = 0.034$, sensitivity 88.9, specificity 71.4%) and pancreas-to-muscle SI ratio more than 0.99 ($p = 0.05$, sensitivity 90.5%, specificity 77.8%) are suggestive of soft pancreas. Histopathological analysis for fat quantification and fibrosis of surgical specimen was also done in these patients. Inter-observer agreement was also calculated for various findings and it was found to have moderate to strong agreement (κ value 0.5–1.0).
**Conclusion:** We recommend duct diameter > 2.6mm, MR Elastography > 2.65kPa and pancreas to muscle SI < 0.99 for predicting firm pancreas.

**09:00 – 10:30** **E 103/104**

*Scientific Session SS 6*

### Liver steatosis, MASLD, fibrosis

### SS 6.1

### A prospective comparison of two ultrasound attenuation imaging modes using different frequencies for assessing hepatic steatosis

H. Jang, J. Jang*; Seoul/KR*

**Purpose:** To compare diagnostic performance between two attenuation imaging modes for assessing hepatic steatosis, using histopathology as the reference standard.
**Material and Methods:** This prospective single-center study included participants with suspected metabolic dysfunction-associated steatotic liver disease (MASLD) who were scheduled for liver biopsy or surgery between June 2023 and June 2024. ACs were consecutively measured using the penetration and general modes of ATI, along with simultaneous measurement of skin-to-region of interest distance (SRD). Spearman correlation was used to evaluate the relationships of AC with histopathological hepatic fat fraction (HFF) and SRD, while linear regression was used to identify factors affecting AC. Diagnostic performance was assessed using area under the receiver operating characteristics curve (AUROC).
**Results:** A total of 119 participants (mean age, 37.2 ± 12.0 years; 87 men) were included, with 73 (61.3%) diagnosed with MASLD. HFF ranged from 0% to 50%. Despite a greater depth of measurement in penetration mode, AC values were significantly higher than in general mode (0.61 vs. 0.54 dB/cm/MHz) (both P < .001). HFF significantly affected AC in both modes, while SRD impacted AC in general mode (both P < .001). AC was positively correlated with HFF in both modes (rs > 0.514; P <.001), and negatively correlated with SRD in general mode (rs = −0.338; P < 0.001). The AUROC for hepatic steatosis did not significantly differ between the two modes (0.751 vs. 0.771; P = .61).
**Conclusion:** Penetration mode produced higher ACs than general mode. Given the negative correlation between SRD and AC in general mode, penetration mode may be more suitable for assessing hepatic steatosis in patients with MASLD, particularly those with thick subcutaneous fat.

### SS 6.2

### Quantification of hepatic steatosis by ultrasound before bariatric surgery: role of the attenuation imaging using MRI as a reference

A. Soler-Perromat, J. Rimola, M. Moreno, A. De Hollanda, A. Pané, M.-Á. García-Criado*; Barcelona/ES*

**Purpose:** To evaluate the applicability of the attenuation imaging (ATI) technique to quantify hepatic steatosis by ultrasound in patients with severe obesity. To establish the prevalence of steatosis and hepatic fibrosis in obese patients undergoing bariatric surgery.
**Material and Methods:** Prospective observational single-center study involving patients eligible for bariatric surgery. Attenuation coefficient measurements were performed using ATI algorithm, and liver stiffness measurements were done using two-dimensional shear wave elastography (2D-SWE). Magnetic resonance imaging proton density fat fraction (MRI-PDFF) and MRI elastography (Elasto-MR) served, respectively, as the reference standards. These measurements were both done before bariatric surgery.
**Results:** Seventy-five individuals were included (median age: 50 years, 66% female, median BMI: 41.7 kg/m$^2$). Steatosis grades (MRI-PDFF): none (36%), mild (39%), moderate (19%) and severe (6%). Attenuation coefficient (ATI, median): 0.76 dB/cm/MHz (IQR: 0.65–0.81). Liver stiffness (2D-SWE, median): 6 kPa (IQR: 5.1–6.5). Liver steatosis (MRI-PDFF, median): 2.28 (IQR: 2.10–3.03). Liver stiffness (Elasto-MR, median): 2.28 kPa (IQR: 2.10–3.03). Patients with higher attenuation coefficient (ATI) values exhibited higher MRI-PDFF (p < 0.0001).
**Conclusion:** ATI technique is applicable for quantifying hepatic steatosis in population with morbid obesity. 64% of the patients showed some degree of steatosis. Only 6% had severe steatosis. Patients with higher attenuation coefficient (ATI) values demonstrated higher MRI-PDFF.

### SS 6.3

### The diagnostic accuracy of quantitative ultrasound with fat fraction parameter for the assessment of hepatic steatosis in patients with metabolic dysfunction-associated fatty liver disease

A. Blandino, R. Cannella, G. Mattana, A. Tulone, S. Petta, T. Bartolotta*; Palermo/IT*

**Purpose:** To investigate the performance of quantitative ultrasound with US Fat Fraction (USFF) in patients with metabolic dysfunction-associated steatotic liver disease (MASLD).
**Material and Methods:** Patients with MASLD prospectively underwent same-day MRI (3T), using the mDixonQuant sequence for fat fraction quantification, and quantitative ultrasound, consisting of Tissue Attenuation Imaging (TAI), Tissue Scatter-distribution Imaging (TSI), and USFF, performed by a radiologist and a radiology resident to assess inter-reader reliability. Spearman's rank-order correlation was calculated between USFF and MRI fat fraction. The diagnostic performance was investigated with the area under the receiver operating characteristics curve (AUC), sensitivity and specificity according to the optimal cutoff.
**Results:** Sixty-four patients (43 males, median age: 60 years) were enrolled. Among them, 51 (79.7%) had grade ≥1 steatosis and 24 (37.5%) had grade ≥2 steatosis. There was a high positive correlation between USFF and MRI fat fraction (rho 0.856, p < 0.001). For the diagnosis of grade ≥1 steatosis, the AUCs were 0.908 (95% CI: 0.809, 0.966) for TAI, 0.802 (95% CI: 0.648, 0.891) for TSI, and 0.912 (95% CI: 0.814, 0.968) for USFF. An USFF > 8.0 had a sensitivity of 86.27% and a specificity of 84.62% for grade ≥1 steatosis. For the diagnosis of grade ≥2 steatosis, the AUCs were 0.953 (95% CI: 0.869, 0.990) for TAI, 0.878 (95%CI: 0.772, 0.947) for TSI, and 0.950 (95% CI: 0.865, 0.989) for USFF. An USFF > 13.7 had a sensitivity of 91.7% and a specificity of 90.0% for grade ≥2 steatosis. Reproducibility of USFF was excellent (ICC: 0.98; 95% CI: 0.97, 0.99).
**Conclusion:** USFF provides excellent performance and reproducibility for quantifying hepatic steatosis in patients with MASLD.

### SS 6.4

### Ultrasound hepatorenal index for the diagnosis of steatosis in patients with type 2 diabetes: a prospective validation study

M. Hamadouche[1], V. Vilgrain[1], C. Hobeika[1], L. Castera[1], D. Valla[1], P.-E. Rautou[1], P. Garteiser[2], B. Van Beers[1], J.-M. Correas[2], M. Ronot[1], M. Dioguardi Burgio[1]*; [1]Clichy/FR, [2]Paris/FR*

**Purpose:** To evaluate the performance of the ultrasound hepatorenal index (US-HRI) for the diagnosis of hepatic steatosis in a prospective cohort of patients with metabolic dysfunction-associated steatotic liver disease (MASLD) and type 2 diabetes, using magnetic resonance imaging-proton density fat fraction (MR-PDFF) as reference.
**Material and Methods:** Two cohorts were prospectively recruited in two centers, including 129 and 291 patients in the training and validation cohort, respectively. Hepatic steatosis was classified according to MR-PDFF as S0: ≤6.5%, S1: 6.5–16.5%, S2: 16.5–22%, S3: ≥22. Obuchowski measurement (OB) was performed to assess diagnostic performance of US-HRI in the whole cohort. Receiver operating characteristic curve analysis was performed to identify optimal cut-offs (maximization of Youden index) of US-HRI for diagnosing ≥S1 and ≥S2 in the training cohort. Optimal cut-offs were then applied to the validation cohort.
**Results:** OB diagnostic performance of US-HRI (four class problem S0 to S3) was 0.79 +/− 0.01. Pairwise accuracy was good for S0 vs the other grades (0.78 to 0.89), but decreased for differentiating S1 from higher grades (0.54 to 0.62). Optimal US-HRI threshold for diagnosing ≥S1 was 1.16, with a sensitivity of 82.7% (95% CI 77.4–87.3), specificity of 80.0% (95% CI 56.3–94.3), and area under the curve (AUC) of 0.814 (95%CI 0.761–0.859) in the validation cohort. For diagnosing ≥S2, the threshold was 1.47, with a sensitivity of 49.0% (95% CI 39.1–59.0), specificity of 73.0% (95% CI 65.3–79.7), and AUC of 0.610 (95% CI 0.548–0.669) in the validation cohort.
**Conclusion:** US-HRI was a reliable screening tool for diagnosing steatosis in MASLD patients with type two diabetes. However, its performance in assessing higher degree of steatosis was inadequate.

### SS 6.5

### Correlation of fatty liver with myosteatosis and liver function test on PDFF-MRI

B. Yetim[1], E. Aydin[1], R.E. Buyuktoka[1], S. Sahin[2]; [1]Izmir/TR, [2]Elazıg/TR

**Purpose:** We assessed the effect of fatty liver disease on the degree of abdominal myosteatosis and liver function tests.
**Material and Methods:** Images of 66 patients were retrospectively analysed. Each patient underwent 1.5T abdominal MRI; the acquisition protocol included axial proton density fat fractionation (PDFF). 36 Patients were classified as fatty liver (more than %6.5 steatoz) and 30 patients as normal liver (less than %6.5 steatosis). PDFF measurements were performed by placing region of interest (ROI) in right lobe of liver. Autochthonous back muscles, quadratus lumborum and psoas major were drawn with free hand ROI at the L3 vertebral level and the percentage of myosteatosis was calculated. ALT and AST values were noted.
**Results:** The mean age of the patients was 54 ± 13 years and the female/male ratio was (28/8) in the patient group and (21/9) in the control group. ALT levels were significantly higher in the patient group (25.81 ± 12.40 U/L) compared to the control group (18.27 ± 6.35 U/L) (p = 0.04). Similarly, AST levels were significantly higher in the patient group (30.18 ± 20.10 U/L) compared to the control group (14.27 ± 5.82 U/L) (p < 0.001). When muscle groups were evaluated, no significant difference was found between the patient and control groups in terms of autochthonous back muscles (19.04 ± 9.51 vs. 17.31 ± 7.52, p = 0.420), quadratus lumborum (15.22 ± 5.66 vs. 13.12 ± 4.69, p = 0.110) and psoas muscle (12.34 ± 4.51 vs. 10.45 ± 4.57, p = 0.097).
**Conclusion:** Liver fat percentage was significantly and positively correlated with ALT and AST. In contrast, it was not correlated with the myosteatosis. Further studies with more patient groups are needed.

### SS 6.6

### Assessing the influence of major hepatic vessel on proton density fat fraction and R2* quantification using automatic whole liver segmentation in MR images

A. Jimenez-Pastor, B. Pereira, A. Alberich-Bayarri, D. Marti-Aguado, L. Marti-Bonmati; Valencia/ES

**Purpose:** Multi-echo chemical-shift encoded MR sequence enables simultaneous quantification of proton density fat fraction (PDFF) and R2*. In recent years, automatic segmentation algorithms have been developed to evaluate liver fat and iron, some including large hepatic vessels within the analyzed region. The bias introduced by this inclusion remains unexamined. This study evaluates the differences in PDFF and R2* quantification when analyzing the entire hepatic volume versus excluding hepatic vessels.
**Material and Methods:** This prospective, multicentric study included patients with chronic liver disease recruited between 2017 and 2024, who underwent MRI and liver biopsy. MR series were automatically segmented using QP-Liver® (Quibim, Valencia, Spain). For comparison, masks with and without hepatic vessels exclusion were generated. PDFF and R2* values were computed for both masks. Statistically significant differences and correlations were assessed with the Mann–Whitney U test and Spearman's rank correlation coefficient due to non-normal distributions (Shapiro–Wilk's test).
**Results:** A total of 165 patients (58% women, 54.67 ± 11.92 years) with varying histological steatosis grades (42.4% S0, 20.0% S1, 20.0% S2, 17.6% S3) and iron overload (78.79% Fe0, 6.06% Fe1, 4.85% Fe2, 6.67% Fe3, 3.64% Fe4) were included. The PDFF and R2* values, using masks with and without the hepatic vessel exclusion, were 10.78 ± 4.68% vs. 10.71 ± 4.57% and 58.16 ± 27.34 s-1 vs. 57.69 ± 26.94 s-1, respectively, with liver volumes of 1425.79 ± 400.73 mL vs. 1452.19 ± 409.69 mL. No statistically significant differences (p < 0.001) and strong correlations (r > 0.999, p < 0.001) were observed for all biomarkers and subgroups.
**Conclusion:** Inclusion of hepatic vessels in MRI analysis does not significantly impacts PDFF and R2* quantification. However, excluding them is recommended to enhance radiologist confidence and improve user experience.

### SS 6.7

### Lipid droplet size explains discordances between histology and magnetic resonance imaging proton density fat fraction in steatotic liver disease

D. Marti Aguado[1], C. Alfaro-Cervello[1], M. Fernandez-Paton[1], A. Ten-Esteve[1], A. Sanchez-Martin[1], A. Crespo[1], I. Navarrete-Perez[1], C. Mongort[1], M. Ballester[1], A. Perez Girbes[1], C. Monton[1], J. Perez-Rojas[1], P. Victor[1], A. Ferrandez[1], V. Aguilera[1], D. Escudero Garcia[1], S. Benlloch[1], A. Jimenez-Pastor[1], A. Alberich[1], C. Sirlin[2], L. Marti-Bonmati[1]; [1]Valencia/ES, [2]San Diego, CA/US

**Purpose:** To evaluate with digital image analysis (DIA) whether the size distribution of lipid droplets (LDs) is associated with disagreements between histology and magnetic resonance imaging proton density fat fraction (MRI-PDFF)-derived hepatic steatosis grades.
**Material and Methods:** This is a prospective, multicentric study of 355 patients with chronic liver disease, having paired liver biopsy and MRI. Using conventional microscopy, steatosis was graded by pathologists (S0-S3), based on the proportion of hepatocytes containing large LDs. DIA automatically segmented and quantified LDs as the proportionate area of tiny (<1 μm²), small (1–90 μm²), large (> 90 μm²), and total droplets. MRI-PDFF graded steatosis as PDFF-S0 < 5.75%; PDFF-S1 ≥ 5.75%; PDFF-S2 ≥ 15.5%; PDFF-S3 ≥ 21.35%. Multivariable modeling was performed to identify predictors of discordance between histology- and PDFF-derived steatosis grade.
**Results:** DIA showed that as steatosis accumulates in the liver, the size of LDs progressively increases. Histology- and PDFF-derived steatosis grades were discordant in 50% (n = 178). The independent predictors of discordances included greater proportionate areas of tiny and small LDs, and lower proportionate area of total LDs. Within histology-derived S0, disagreements were due to higher MRI grading having higher proportionate areas of tiny and small LDs. Within histology-derived S2 and S3, disagreements were due to lower MRI grading having lower proportionate areas of large and total LDs.
**Conclusion:** LDs size distribution explains the discordances between histology and MRI-PDFF. While PDFF captures the whole spectrum of LDs size, pathologists underestimate steatosis grade in patients with abundant tiny and small LDs, as these are disregarded in their scoring system. Conversely, conventional histology overestimates steatosis grade in patients with abundant large LDs, indicating a bias in subjective visual assessment.

### SS 6.8

### MRI assessment of liver steatosis following pancreatic surgery

A. Hoppe, V. Englmaier, J. Jonas, D. Stocker, S. Stocker-Ghafoor; Zurich/CH

**Purpose:** To evaluate the incidence and severity of liver steatosis on MRI following pancreatic surgery and to analyze associated clinical factors.
**Material and Methods:** This retrospective single-center institutional review board-approved study included patients with pancreatic surgery (August 2009–September 2022) and pre- and post-operative MRI (mean time-interval: 3.3 years). Circular region-of-interest measurements on in- and opposed-phase sequences assessed hepatic fat fraction (HFF; mild: 5–15%, moderate: 15–30%, severe: >30%). Measurements were taken at two points in each liver lobe. Clinical data, including age, gender, BMI, diagnosis, and surgery type, were collected.
**Results:** Ninety patients (mean age: 61 years, BMI: 24.5 kg/m², 42% female) were evaluated. Pre-existing steatosis was present in 24.4%, 13.6% of which showed grade progression post-operatively. De-novo liver steatosis developed in 10.3%, all cases being mild and associated with malignant (n = 6) or borderline malignant (n = 1) lesions. Most patients (71.4%) with de-novo steatosis underwent non-head-sparing pancreatic surgery. Mean pre-operative BMI was significantly higher in patients with de-novo steatosis (26.4 ± 3.6 vs. 22.9 ± 2.8 kg/m²; p = 0.003). Diabetes mellitus pre-existed in 14.6%, and developed de-novo in 13.3%, 70% of which had non-head-sparing pancreatic surgery.
**Conclusion:** De-novo liver steatosis occurred in 10% of patients following pancreatic surgery, all with malignant or borderline diagnoses. Most patients with de-novo liver steatosis underwent non-head-sparing surgery, and affected patients had a significantly higher BMI. Further studies in this context are warranted to facilitate early identification of patients at risk for the development of liver steatosis following pancreatic surgery.

### SS 6.9

### Epicardial and pericoronary adipose tissue as predictors of liver disorders

T. Polidori, D. De Santis, B. Masci, M. Polici, G. Tremamunno, C. Santangeli, L. Capece Minutolo del Sasso, M. Zerunian, D. Caruso, A. Laghi; *Rome/IT*

**Purpose:** Hepatic steatosis and fibrosis are critical characteristics in liver disease progression, often leading to severe liver dysfunction and increased mortality. While the role of systemic metabolic factors in liver disease is well established, the influence of local fat depots, such as epicardial adipose tissue (EAT) and pericoronary adipose tissue (PCAT), remains largely unexplored. This study aims to assess the potential of EAT and PCAT as independent risk factors for hepatic steatosis and fibrosis.
**Material and Methods:** We conducted a prospective study on 108 patients undergoing coronary computed tomography angiography and liver magnetic resonance imaging (MRI). EAT and PCAT volumes were quantified using semi-automated segmentation techniques, while hepatic steatosis and fibrosis were assessed via MRI. Statistical analyses, including multivariate logistic regression, were performed to evaluate the relationship between cardiac adipose tissues and liver pathology.
**Results:** Hepatic fibrosis was significantly associated with increased volume of EAT ($p < 00001$), and the ROC curve analysis indicates good predictive performance for EAT volume (AUC = 0.889; $p < 0.01$) and EAT fat ratio (AUC = 0.844; $p < 0.01$). In contrast, PCAT did not show significant correlation with liver fibrosis. Liver steatosis did not exhibit a significant correlation with either EAT or PCAT. These findings suggest that EAT may play a more substantial role in hepatic fibrosis than in steatosis.
**Conclusion:** EAT and PCAT are potential biomarkers for hepatic fibrosis, with EAT possibly exerting a systemic influence beyond cardiovascular health. Further research is warranted to explore the mechanisms underlying this relationship and its implications for liver disease management.

### SS 6.10

### Post-Covid-19 liver injury: long-term multiparametric ultrasound follow-up with updated fibrosis quantification

M. Radzina, D. Putrins, I. Vanaga, O. Kolesova, L. Viksna; *Riga/LV*

**Purpose:** To evaluate multiparametric ultrasound (mpUS) markers of liver injury in patients following COVID-19 infection.
**Material and Methods:** MpUS was performed on patients 3–6 months after COVID-19 and repeatedly 3 years later, including stiffness evaluation (2D-SWE), dispersion imaging (SWD) and quantitative attenuation analysis (ATI) using the same scanner system. Results were stratified according to the SRU's consensus "Rule of four."
**Results:** A total of 58 patients were evaluated (28 women and 30 men) with a mean age of 46.4 ± SD 12.9 years. In the 3-year follow-up, mean attenuation values increased from 0.56 to 0.60 dB/cm/MHz ($p < 0.001$), whereas mean stiffness values decreased from 5.09 to 4.60 kPa ($p < 0.001$). Dispersion values remained unchanged ($p > 0.05$). The difference between liver stiffness groups was insignificant ($p > 0.05$). Compensated advanced chronic liver disease (cACLD) could be ruled out in the majority of patients (97%). Two patients had liver stiffness values that could be indicative of cACLD.
**Conclusion:** Slight long-term increase in hepatic attenuation was observed in patients after COVID-19 infection, which, although under the threshold for hepatic steatosis, could indicate initial stages of increased hepatic fat accumulation. Conversely, the slight decrease in liver stiffness values could suggest reduction or resolution of hepatic inflammation; however, the clinical significance of this is unclear with further research warranted for precise evaluation of the cellular mechanisms in long-term liver injury.

**09:00 – 10:30** **E 105/106**

*Scientific Session SS 7*

### Bile ducts and gallbladder

### SS 7.1

### Clinical feasibility of accelerated 3-dimensional sampling perfection with application-optimized contrasts using different flip angle evolutions with deep learning reconstruction in MR cholangiopancreatography: comparison of image quality

S. Bae[1], J. Hwang[1], S. Hong[1], E. Lee[1], M. Paek[1], D. Nickel[2]; *[1]Seoul/KR, [2]Erlangen/DE*

**Purpose:** To evaluate the clinical feasibility of accelerated 3D sampling perfection with application-optimized contrasts using different flip angle evolution (3D SPACE) with deep learning reconstruction in MR cholangiopancreatography (DL-MRCP) and to compare the image quality and acquisition time of DL-MRCP with conventional respiratory-triggered 3D MRCP and breath-hold compressed-sensing (BH-CS) 3D MRCP.
**Material and Methods:** This prospective study, approved by our institutional review board, included 75 consecutive patients who underwent 3T abdominal MRI with informed consent. MRCP was performed using BH-CS, conventional 3D SPACE, and 3D SPACE with DL reconstruction. Image quality (overall image quality, delineation and sharpness of ductal structures, background suppression, motion artifact, and noise) was assessed by two radiologists using a 5-point scale and compared among three MRCP sequences. Subgroup analysis was performed for elderly patients (≥70 years, n = 22).
**Results:** Mean acquisition time for DL-MRCP (161.08 ± 51.73 sec) was significantly shorter ($P < 0.05$) than conventional MRCP (395.73 ± 138.59 sec). BH-CS-MRCP (16 sec) had the shortest acquisition time, though it was not feasible in six patients due to difficulty in breath hold, five of whom were elderly. DL-MRCP showed superior image quality scores compared to conventional MRCP for all parameters ($P < 0.05$). DL-MRCP outperformed BH-CS-MRCP in pancreatic duct delineation and background suppression, with no significant difference in other quality parameters. In the elderly subgroup, DL-MRCP provided better overall image quality than BH-CS-MRCP ($P < 0.05$).
**Conclusion:** DL-MRCP is a clinically feasible and efficient technique, particularly for elderly patients, offering comparable image quality to BH-CS-MRCP and superior quality with a significant reduction in acquisition time compared to conventional MRCP.

### SS 7.2

### Inter- and intra-reader agreement of ANALI and district scores in patients with primary sclerosing cholangitis

N. Kutaiba, T. Goodwin, B. Paulino Di Muzio, S.-H. Winata, R.P. Lim, M. Goodwin, W. Kemp, S. Roberts, A. Majeed, N. Tan; *Melbourne/AU*

**Purpose:** Assessment of severity of primary sclerosing cholangitis (PSC) using magnetic resonance cholangiopancreatography (MRCP) is prone to subjective interpretation and intra/inter-reader variability. Radiological severity scores including ANALI and DiStrict scores have been proposed as objective tools to correlate with clinical outcomes. We aimed to assess the inter- and intra-reader agreement of these scores among five radiologists in a multicentre Australian cohort of PSC patients.
**Material and Methods:** This is a retrospective study of patients with PSC who underwent MRCP between 2006 and 2018 at three metropolitan centres. Five radiologists assessed MRCPs and one radiologist repeated their assessment. ANALI (without gadolinium) and DiStrict scores were calculated for each MRCP. Inter- and intra-reader agreement was assessed using intra-class correlation (ICC) coefficient.
**Results:** We included 92 patients (mean age 41.7 [SD ± 18]; males 52 [56.5%]; cirrhosis 49 [53.2%]; IBD 61 [66.3%]). For ANALI score, the inter-reader agreement was moderate (ICC, 0.72; 95% CI, 0.64–0.78) and the intra reader agreement was good (ICC, 0.86; 95% CI 0.80–0.90). For DiStrict score, the inter- and intra-reader agreement were both moderate (ICC, 0.67; 95% CI 0.58–0.74; and ICC, 0.63; 95% CI 0.34–0.78, respectively).
**Conclusion:** Both ANALI and DiStrict scores demonstrate moderate inter- and moderate to good intra-reader agreement. This allows for confidence in using such scores in clinical practice. Future research and comparison of the prognostic value of these scores are needed.

### SS 7.3

### Validation of district score, a classification and prognostic score for individuals with primary sclerosis cholangitis

A. Grigoriadis[1], S. Hamma[1], G. Kemmerich[2], I. Björk[2], K. Horsthuis[3], J. Reimerink[3], S. Jiménez-Serrano[4], G. Rafart Martinez[4], M. Seager[5], M. Limbada[5], C. Singh[6], S. Czerniak[6], D. Joshi[5], I. Olivas[4], T. Middelburg[3], M. Londoño[4], K. Kaasen Jørgensen[7], T. Folseraas[2], J. Nayagam[5], D. Assis[6], C. Ponsioen[3], M. Vesterhus[8], A. Bergquist[1]; *[1]Stockholm/SE, [2]Oslo/NO, [3]Amsterdam/NL, [4]Barcelona/ES, [5]London/UK, [6]New Haven, CT/US, [7]Lørenskog/NO, [8]Bergen/NO*

**Purpose:** To validate the reproducibility and prognostic value of the DiStrict-score in a multicenter international cohort.
**Material and Methods:** The DiStrict-score is an MRCP-based classification of the severity of ductal changes (range 1–8) for individuals with primary sclerosing cholangitis (PSC), is reproducible, and is associated with liver-transplantation and liver-related death. However, it has not been validated. For this retrospective multicenter study, hepatologists from eight international PSC-centers retrieved data from consecutive adult PSC individuals (MRCP, demographics, liver-tests, hepatobiliary cancer development, liver transplantation, death, cause of death). Two radiologists from each center independently applied the DiStrict-score to individuals of their center. Cases of disagreement were resolved by consensus. Interreader agreement was assessed with the intraclass correlation coefficient. The association of DiStrict-score with outcomes (transplant-free survival, hepatobiliary malignancy development) was assessed with Cox-regression. Survival estimates were calculated with Kaplan–Meier curves, and the curves were compared with log-rank test.
**Results:** Four hundred-fifteen individuals (271 males, 248 with ulcerative colitis) with a median diagnosis age of 39 years were included. During a median follow-up time of 84 months, 101 individuals developed outcomes (liver transplantation; n = 78, liver-related death; n = 10, hepatobiliary malignancy; n = 13). The interreader agreement ranged between 0.61 and 0.91 for the different centers. Individuals with high DiStrict-scores (5–8) had worse survival (log-rank test; $p < 0.001$), and a higher risk of developing outcomes compared to individuals with low scores (1–4), with a hazard ratio of 1.98 (95% CI; 1.32–2.96, p = 0.001).
**Conclusion:** The DiStrict-score is reproducible and associated with transplant-free survival, and development of hepatobiliary malignancy in individuals with PSC.

### SS 7.4

### Prevalence, prognostic value, and interreader agreement of high-grade and relevant strictures in individuals with primary sclerosing cholangitis

S. Hamma, C. Villard, A. Bergquist, A. Grigoriadis; *Stockholm/SE*

**Purpose:** To assess the agreement of the identification, and the association of high-grade and relevant strictures with outcomes in individuals with primary sclerosing cholangitis (PSC).
**Material and Methods:** Relative strictures were defined according to their original definitions as extrahepatic strictures (AASLD) or high-grade strictures (EASL) with signs or symptoms of obstructive cholestasis and/or bacterial cholangitis. Two radiologists independently evaluated MRCPs of consecutive individuals with PSC for the presence of extrahepatic, and high-grade strictures. Clinical-laboratory and outcome data (liver-transplantation, liver-related death, hepatobiliary cancer) were collected. Interreader agreement was assessed with the intraclass correlation coefficient (ICC). The association of strictures with outcomes was evaluated with Cox-regression. Survival estimates were assessed with Kaplan–Meier curves, and the curves were compared with log-rank test.
**Results:** One hundred seventy-one individuals with PSC (107 males) were included. Eighty had high-grade strictures, 39 and 53 had relevant strictures according to EASL and AASLD definitions, respectively. During a median follow-up of 124 months, 50 individuals developed outcomes (liver transplantation = 37, liver-related death = 5, hepatobiliary cancer = 8). Agreement for identification of high-grade strictures was moderate (ICC = 0.72; 95% CI 0.64–0.78). High-grade strictures were associated with worse survival but were not independently associated with outcomes. Individuals with relative strictures had poorer survival and relative strictures were independently associated with outcomes (HR EASL = 2.12, 95% CI 1.13–3.98; HR AASLD = 2.15, 95% CI 1.16–3.98).
**Conclusion:** Relevant and high-grade strictures are common and can be identified with satisfactory agreement. Relevant strictures are independently associated with outcomes, and worse survival. High-grade strictures are associated with worse prognosis; however, they are not independent predictive factors.

### SS 7.5

### Predicting prognosis of primary sclerosing cholangitis with gadoxetate-enhanced MRI

K. Sandrasegaran, A. Silva, C.P. Thompson, A.N. Silva, S.R. Bonkur, A.S. Shah, E.J. Carey, C.R. Jayasekara; *Scottsdale, AZ/US*

**Purpose:** We wanted to determine if parameters from gadoxetate-enhanced MRI were able to predict adverse outcomes in PSC patients, independently of clinical and imaging scoring systems.
**Material and Methods:** In this institutional review board-approved retrospective case-controlled study, 55 patients with gadoxetate-enhanced MRI were enrolled. Three radiologists independently determined the presence of gadoxetate in the proximal and distal common bile duct (CBD) at 5 and 20 minutes after contrast, as well as relative enhancement indices of CBD and liver. MRI parameters were compared with clinical scoring systems (Mayo Risk Score, MELD-3.1, Amsterdam-Oxford PSC Score, UK PSC Score) and imaging scoring systems (FLIS and ANALI with Gadolinium) for prediction of adverse outcomes within two years of MRI.
**Results:** Mean age of patients was 41.1 years. There were 32 males and 23 females. Adverse outcomes within 2 years of MRI were death (n = 4), sepsis (n = 12), need for transplantation (n = 7), variceal bleeding (n = 3). On logistic regression analysis, only Mayo Risk Score and the presence of gadoxetate in distal CBD at 20 minutes predicted need for transplant (p = 0.01 and 0.02, respectively). The sensitivity and specificity of gadoxetate in the distal CBD for predicting transplantation were 86.7% and 83.0%, respectively. The area under ROC curve (95% confidence interval) was 0.84 (0.72–0.93). None of the variables were retained in the model when predicting death or sepsis.
**Conclusion:** Gadoxetate-enhanced MRI parameters, in particular the presence of contrast in the distal CBD at 20 minutes, give useful prognostic information in PSC patients, which may be equivalent or superior to clinical and imaging scoring systems.

### SS 7.6

### Multiparametric MRI in IgG4-related sclerosing cholangitis and autoimmune pancreatitis: novel quantitative metrics for disease assessment

M. Pansini[1], E. Jackson[2], Y. Deng[2], A. Triay-Bagur[2], G. Ralli[2], A. Bailey[2], S. Olodo-Atitebi[2], E. Selvaraj[3], M. Pavlides[2], H. Bungay[2], E. Culver[2]; *[1]Lugano/CH, [2]Oxford/UK, [3]Warwick/UK*

**Purpose:** To evaluate standardized quantitative MRI metrics for characterizing IgG4-related sclerosing cholangitis and autoimmune pancreatitis (IgG4-SC/AIP), distinguishing IgG4-SC from primary sclerosing cholangitis (PSC) and monitoring disease activity.
**Material and Methods:** Ten IgG4-SC/AIP patients underwent multiparametric MRI including standardized scanner-referenced T1 mapping (srT1) of pancreatic parenchyma and quantitative MRCP analysis. Pancreatic and common bile duct metrics (diameter, visible length) were compared with age-matched PSC patients (n = 10) and healthy volunteers (n = 10) from the MoBILISE study. Pancreatic srT1 values and volume indices were compared with age, sex, and BMI-matched healthy (n = 100) and type 2 diabetic (n = 100) controls from UK Biobank.
**Results:** Pancreatic srT1 was significantly elevated in active IgG4-SC/AIP compared to matched controls (1371 ms in active disease vs 744 ms healthy, 757 ms diabetic; $p < 0.001$). All patients with active disease had srT1 values above the abnormality threshold of 836 ms. Active disease showed characteristic pancreatic duct changes with either compression (reduced diameter and visible length) or focal dilation. Common bile duct diameter was elevated in IgG4-SC/AIP patients compared to PSC and healthy controls (median 6.1 mm vs 4.7 mm PSC, 5.0 mm healthy; $p < 0.05$), regardless of disease activity. Pancreatic volume index (ml/kg) was significantly lower in active disease compared to inactive disease and controls.
**Conclusion:** Multiparametric MRI metrics reveal characteristic patterns distinguishing active IgG4-SC/AIP from PSC and controls, offering potential objective measures for disease monitoring and differential diagnosis.

### SS 7.7

### Risk of malignancy in gallbladder polyps in patients with primary sclerosing cholangitis: a systematic review and meta-analysis

S. Curran, M. Campbell, D. Gleeson, B. Rea*; Sheffield/UK*

**Purpose:** Gallbladder polyps (GBP) in patients with primary sclerosing cholangitis (PSC) are associated with increased risk of malignancy. However, research is often limited to single-centre, retrospective, observational studies. We aimed to estimate the risk of malignancy in GBP in PSC, and evaluate size as a prognostic factor.

**Material and Methods:** This PROSPERO-registered systematic review and meta-analysis included patients >16 years with PSC and confirmed GBP on imaging. Metastatic gallbladder cancers (GBC) were excluded. Demographic details, GBP size, imaging findings, surgical outcomes and histology post-cholecystectomy were collected.

**Results:** An initial search identified 1040 abstracts, and following blinded screening, 13 papers were included. In total, 231 patients with polyps were recorded with an overall risk of malignancy of 25.1%. Polyp size data were available for 75 patients [16mm (2–55 mm); median (range)], including 44 cancers [20 mm (4–55 mm)]. For every 1mm increase in polyp size, the risk of GBC increased by 9% with good predictive ability (p = 0.002; AUROC 0.72). Positive predictive value for malignancy in polyps < 10mm was 27%, rising to 88% in polyps > 30 mm (p = 0.006). In contrast, gender and age were not significant predictors of malignancy (p > 0.05). Polyp size > 10mm had a sensitivity of 91% and specificity 35% for GBC, with a relative risk for malignancy (over polyps < 10 mm) of 2.5.

**Conclusion:** A paucity of literature surrounding GBP in PSC exists, with low level evidence. In this meta-analysis, we demonstrated GBP in PSC are associated with a high risk of malignancy, with increasing size being a significant prognostic indicator. GBP > 10mm were two-and-a-half times more likely to contain cancer.

### SS 7.8

### Are intrahepatic cholangiocarcinoma subtypes associated with CT imaging features? An investigation of small duct versus large duct ICC in a Western population

W. Law, S. Lobaugh, C. Sigel, M. El Homsi, A. Araji, P. Argiriadi, J. Bou Ayache, S. Chhabra, G. Levin, T. Kim, A. Rosen, S. Chahwan, G. Barraza, M. Capanu, Y. Song, B. Jarnagin, R. Do*; New York, NY/US*

**Purpose:** Few studies conducted in Eastern cohorts suggested intrahepatic small duct (SD) intrahepatic cholangiocarcinoma (ICC), which has better prognosis than large duct (LD), was associated with peripheral location, targetoid enhancement, round/lobulated contour and lack of bile duct encasement. This study aims to investigate the imaging features of ICC on contrast-enhanced CT that may differentiate SD from LD in a Western cohort.

**Material and Methods:** All patients with pathologically proven ICC at a single center from 09/2010 to 08/2020 were included if prospectively subtyped by a single pathologist as LD, SD, or indeterminate. Pretreatment CTs were retrospectively reviewed by 8 radiologists, blinded to subtype, for lesion morphology, enhancement pattern, and other features of the liver and locoregional nodes. Samples classified as indeterminant for SD/LD (n = 61), or had no technically adequate pretreatment CT (n = 250), were excluded from analysis. Univariable analysis (Fisher's exact and Wilcoxon rank sum tests) and interreader agreement (k) were performed.

**Results:** 216 ICCs were included (male [50%], median age 64 [range 23–86], clinical stages III/IV [69%], Caucasian [79%]), with 179 (83%) SD and 37 (17%) LD. SD was more likely than LD to have CEA < 5 ug/L (80% vs 20%), larger tumor size [8.6 (1.2–20.0) vs 7.0 (2.0–15.2) cm, p = 0.008)], mass-forming (98% vs 76%, p < 0.001), lobulated/round shape (68% vs 49%, p = 0.036), sharp margins (65% vs 35%, p = 0.001), and less likely to have biliary dilatation (35% vs 60%, p = 0.006). Interreader agreement (n = 60) was slight to almost perfect agreement (k = 0.315, 95% CI [0.157, 0.5] to 0.946, 95% CI [0.911, 0.968]).

**Conclusion:** Morphologic CT features of larger tumor size, mass-forming, lobulated or round shape, sharp margins, and lack of biliary dilatation were associated with SD- compared to LD-ICC.

### SS 7.9

### Proposal of a densitometric criterion for the evaluation of response in patients with cholangiocarcinoma treated with radioembolization and systemic therapy

T. Cascella[1], M. Bruno[1], G. Carletti[1], C. Angelucci[1], V. Magni[1], S. Cvejic[2], L. Saggiante[1], C. Chiesa[1], M. Vaiani[1]*; [1]Milan/IT, [2]Belgrad/RS*

**Purpose:** The mRECIST criteria present several limitations in the evaluation of response in patients with mass-forming cholangiocarcinoma treated with systemic therapies and radioembolization. We propose a new criterion based on multiphasic CT densitometric analysis of treated lesions, aimed at better stratifying response.

**Material and Methods:** From June 2014 to May 2024, 25 patients underwent radioembolization after systemic therapy. Chemotherapy consisted of at least four cycle of gemcitabine (1000 mg/m$^2$) and cisplatin (25 mg/m$^2$). Radioembolization was performed with yttrium-90 glass microspheres (20/25 patients) or holmium-166 microspheres (5/25). Radiological response was retrospectively assessed using both mRECIST and a densitometric criterion. Our method quantifies tumor vascularization by drawing a circular region of interest in the most representative slice of the lesion during the arterial phase and on the corresponding slice of the native phase. Baseline CT examinations were compared with CT after treatments. A reduction greater than 15% in arterial enhancement was considered a partial response.

**Results:** Some lesions developed calcification, and tumors treated with holmium-166 showed hyperdense images due to the composition of the microspheres. According to densitometric criteria, partial response was observed in 68% patients, while mRECIST indicated a partial response in only 12%. This difference is statistically significant with p = 0.0001 (Fisher's exact test). Eleven out of 25 patients are still alive, four of whom underwent orthotopic liver transplantation. Histological examination demonstrated objective response, even though mRECIST classified these patients as having stable disease.

**Conclusion:** mRECIST is unable to effectively differentiate responders from non-responder patients. Our criterion could help identify successful downstaging, potentially allowing access to curative treatments such as liver transplantation.

### SS 7.10

### Magnetic resonance cholangiography with and without GD-EOB-DTPA for biliary anatomy evaluation in living liver donors

A. Pecchi, F. Camaione, B. Catellani, S. Di Sandro, F. Di Benedetto, P. Torricelli*; Modena/IT*

**Purpose:** We describe the MR protocol of biliary anatomy study in the healthy donor and to evaluate its accuracy in comparison with intraoperative cholangiography.

**Material and Methods:** 16 candidates for liver donation were studied using 1.5 tesla MR without e with iv contrast injection (Gd-EOB-DTPA). We performed 2D-3D T2w MRC sequences and 3D T1w Gradient Echo axial and coronal sequences acquired in the hepato-biliary phase. The biliary tree anatomy was classified according to Choi and Huang. The information obtained from the integrated analysis of the above MR sequences was then compared with intraoperative cholangiography: the standard of reference.

**Results:** In 7/16 cases, we found a normal anatomy (43.75%). In 9/16 cases, we found a biliary variant classified according to Choi-Huang. The comparison with intraoperative cholangiography confirmed the biliary anatomy outlined on MR examination in 14/16 donors studied. In 8/16 cases (50%), MRC predicted two distinct ducts on surgical slice. The MR analysis of biliary anatomy had a sensitivity of 87.5%, a near specificity at 100% and an accuracy of 93.33%.

**Conclusion:** The integrated use of 2D - 3D T2 weighted and 3D T1 weighted EOB MRC has proved successful in defining the correct biliary anatomy of the donor, helps to increase the radiologist's diagnostic confidence in defining the anatomy and in the surgeon's decision on whether to perform a single or double biliary anastomosis based on the distance of the right ducts on the surgical slice.

**10:00 – 10:45** **Emerald Room**

*Scientific Session SS 16*
### Acute abdomen

### SS 16.1
### The added value of diffusion-weighted imaging combined with T1-weighted fat saturation sequences in the detection of gangrenous cholecystitis
I. Maldonado Schoijet, H. Schulz Ibaceta, M. Arias Graf, J. Alvo*; Santiago/CL*

**Purpose:** This study aims to assess the correlation between gallbladder wall necrosis with the presence of high-signal foci on T1-weighted (T1W) magnetic resonance imaging (MRI) and the added value of combining T1W with fat saturation (FatSat) and diffusion-weighted imaging (DWI) sequences in detecting mural necrosis.
**Material and Methods:** A transversal analysis of adult patients diagnosed with acute cholecystitis (AC) at our institution (2012–2023) who underwent preoperative abdominal MRI and subsequent surgical intervention was performed. We reviewed histopathological specimens to establish the presence of gangrenous cholecystitis. Two radiologists independently reviewed T1W FatSat and DWI sequences for necrosis. Cohen's Kappa coefficient assessed diagnostic performance to evaluate the agreement and accuracy of T1W and the combination of T1W with DWI imaging findings for necrosis.
**Results:** Among the 142 patients who met the inclusion criteria, the study demonstrated that high signal foci in T1W have an agreement of 87% for necrosis, improving to 91% when combining T1W and DWI sequences. High signal foci in T1W showed a sensitivity and specificity of 65% and 92%, respectively, for necrosis, with a positive predictive value (PPV) of 60% and a negative predictive value (NPV) of 93.2%. When combining T1W and DWI, the reported sensitivity and specificity were 56.5% and 97%, respectively, with a PPV of 81.3% and NPV of 92.1%.
**Conclusion:** T1W high-signal intensity is a reliable imaging feature for excluding gangrenous cholecystitis. Adding DWI significantly enhances diagnostic accuracy for detecting mural necrosis in AC, improving the PPV and the agreement with necrosis.

### SS 16.2
### CT-derived body composition as a predictor of acute pancreatitis severity: a post-hoc analysis of a multicenter study of 437 patients
R. Borbély[1], D. Molnár[1], B. Teutsch[1], B. Erőss[1], K. Márta[1], V. Vass[1], Á. Szentesi[1], P. Hegyi[1], Á. Vincze[1], E. Szalai[1], P. Hegyi[1], N. Faluhelyi[2]*; [1]Budapest/HU, [2]Pecs/HU*

**Purpose:** Predicting the severity of acute pancreatitis (AP) remains a significant clinical challenge. While abdominal CT scans are primarily used for diagnostic purposes, their potential for body composition analysis is underutilized. This study aimed to assess the predictive accuracy of CT-derived body composition metrics in determining the severity of AP.
**Material and Methods:** In this post-hoc multicenter study, we analyzed 437 AP patients who underwent CT within 24 hours of admission. Using the Total-Segmentator tool in 3D Slicer, we measured visceral adipose tissue (VAT), subcutaneous adipose tissue (SAT), and skeletal muscle area (SMA) at the third lumbar vertebra (L3). These measurements were normalized for height to calculate the visceral adipose tissue index (VATI), subcutaneous adipose tissue index, and Skeletal Muscle Index (SMI). We also assessed average muscle radiodensity (Hounsfield Units) for fatty infiltration. The fat-to-muscle ratio was calculated as fat area/muscle area. We analyzed the predictive value of these metrics for severe AP using receiver operating characteristic curves and computed the area under the curve (AUC). AP severity was determined using the Modified CT Severity Index (mCTSI). Statistical analyses were performed using IBM SPSS Statistics.
**Results:** The fat-to-muscle ratio and VATI were the top predictors of severe AP, yielding AUCs of 0.68 and 0.65, respectively. Other metrics, such as SMA and SMI, did not show significant predictive value (AUCs 0.52 and 0.54).
**Conclusion:** CT-derived body composition metrics, particularly the fat-to-muscle ratio and visceral adipose tissue index, play significant roles in predicting the severity of acute pancreatitis. Incorporating these measurements into routine CT assessments can enhance prognostic accuracy.

### SS 16.3
### Assessment of small bowel ischemia in mechanical small bowel obstruction: diagnostic value of bowel wall iodine concentration using dual-energy CT
G. Simon, P. Calame*; Besancon/FR*

**Purpose:** The primary aim of our study was to determine whether dual-energy computed tomography (DECT), specifically through bowel wall iodine concentration (BWIC) measurement, was superior to monoenergetic reconstructions (MR) for diagnosing and staging bowel ischemia in patients with mechanical small bowel obstruction (SBO).
**Material and Methods:** From November 2021 to December 2023, all patients with mechanical SBO who underwent contrast-enhanced DECT were evaluated for inclusion. Conventional CT features were first reviewed on 70-keV-MR and 40-keV-MR, followed by BWIC measurements in five regions of interest in the walls of both normal and abnormal small bowel loops. Diagnostic performance of a simple CT score was compared to that of BWIC measurement for diagnosis of bowel ischemia.
**Results:** The study included 142 patients (80 men, 46%; mean age 67 ± 17 (SD) years-old); 56 underwent surgery, 22 of whom had confirmed bowel ischemia, and 12 had necrosis requiring bowel resection. The overall area under the curve (AUC) of the BWIC measurement for diagnosing bowel ischemia was 0.98 (95% confidence interval [CI]: 0.97–1.00), equivalent to the AUC of the simple CT score (0.97; 95% CI: 0.92–1.00). However, BWIC cut-off at 1.16 mgI/mL outperformed subjective assessment of DBE at 70-keV-MR and 40-keV-MR (Youden Index of 0.90 [95% CI: 0.77–0.98] vs 0.54 [95% CI: 0.32–0.76] and vs 0.71 [95% CI: 0.49–0.89], respectively, $P < 0.001$ and $P < 0.001$, respectively).
**Conclusion:** BWIC measurement outperformed subjective assessment of DBE for diagnosing bowel ischemia in SBO and could allowed stratification of ischemia. However, BWIC did not surpass a global comprehensive analysis of conventional CT images.

### SS 16.4
### Proposition of a morbidity–mortality scale for patients with acute mesenteric ischemia
H. Garnier[1], S. Vilain[1], S. Thavarajah[1], A. Nuzzo[1], I. Ben Abdallah[2], J. Grégory[1], M. Dioguardi Burgio[1], M. Ronot[1], L. Garzelli[1]*; [1]Clichy/FR, [2]Paris/FR*

**Purpose:** Acute mesenteric ischemia (AMI) is a severe condition with high mortality rates. Current evaluations of patient outcomes often focus on short-term survival, which does not fully capture the long-term prognosis. A morbidity–mortality scale could enhance outcome assessment and serve as a primary endpoint in future clinical trials. We aimed to propose a morbidity–mortality scale for patients with occlusive arterial AMI, providing a more precise evaluation of long-term outcomes and improving clinical trial accuracy.
**Material and Methods:** This retrospective, single-center study included patients admitted for occlusive arterial AMI at an intestinal stroke center. Clinical, biological, and imaging data, as well as treatments and outcomes, were analyzed. A four-level morbidity–mortality scale was developed based on post-AMI status: 1/ alive without digestive resection, 2/ digestive resection without short bowel syndrome (SBS), 3/ SBS without (3a) or with (3b) home parenteral nutrition (HPN), and 4/ death. The distribution of scale distribution at 3, 6 and 12 months was described.
**Results:** 256 patients (median age: 68; 44% female) were included. Score 4 (death) was 23% at 3 months, 26% at 6 months, and 31% at 12 months. Overall, 149 (58%) patients underwent bowel resection. Scores 1 and 2 were stable over the first year, around 30% and 20%, respectively. Score 3a was rarely observed (2%, 4% and 6% at 3, 6, and 12 months). Score 3b decreased from 22% at 3 months to 15% at 12 months.
**Conclusion:** This new morbidity–mortality scale provides a comprehensive evaluation of long-term outcomes in AMI patients and could serve as a novel endpoint in future therapeutic trials.

### *SS 16.5*
### CT diagnosis of abdominal compartment syndrome
K. Sandrasegaran; *Scottsdale, AZ/US*

**Purpose:** Diagnosing abdominal compartment syndrome (ACS) is challenging in critically ill patients who have other causes for organ dysfunction, such as sepsis, respiratory distress syndrome or hypovolemic shock. This study aimed to identify specific CT imaging findings predictive of ACS.
**Material and Methods:** This retrospective case-control study searched institutional databases to find patients with ACS and a matched control cohort of critically ill patients without ACS. Three radiologists, blinded to the patients' diagnosis and clinical course, reviewed CT scans for 19 different imaging signs.
**Results:** 36 patients with ACS and 37 without ACS were enrolled. A total of 56 CT scans in patients with ACS and 51 in controls were reviewed. On univariate analysis, peritoneal–abdominal height ratio (PAR) ($p = 0.002$), inferior vena cava caliber (IVCC) ($p = 0.001$) and contour changes of solid organs (CCSO) ($p = 0.009$) were significant. On logistic regression analysis, only PAR ($p = 0.02$) and IVCC ($p = 0.03$) were significant. The presence of at least one of the following findings—PAR > 0.57, IVCC < 1.5 mm or CCSO—had a sensitivity of 62% and specificity of 87% for predicting ACS. The presence of two of these signs had a specificity of 100% for ACS.
**Conclusion:** Radiologists should consider ACS in critically ill patients undergoing CT when there is an increased PAR, diminutive IVC caliber and/or solid organ contour change. Recognition of these imaging findings may aid in the timely and accurate diagnosis of ACS in clinical practice.

**11:00 – 12:30** **E 103/104**

*Scientific Session SS 8*
### Rectal cancer

### *SS 8.1*
### Impact of web-based multi-reader studies on local diagnostic practices in rectal cancer: an international survey
E.P. Goedegebuure[1], M.J. Lahaye[1], N. Bogveradze[2], N. el Khababi[3], J. van Griethuysen[1], A. Khmelinskii[1], M. Maas[1], R. Beets-Tan[1], D.M. Lambregts[1]; *[1]Amsterdam/NL, [2]Vienna/AT, [3]Utrecht/NL*

**Purpose:** This survey aimed to investigate the impact of web-based validation studies on (re)staging rectal cancer on the local clinical practice of participating radiologists.
**Material and Methods:** From 2021 to 2023, 38 radiologists from 18 countries participated in various multi-reader studies on (re)staging and response prediction in rectal cancer using a web-based imaging and research platform. Study results were shared with the participants at an individual and group level. Following the publication of the study results, participants were invited to complete a survey examining how their involvement impacted their individual and/or local practices at their hospital.
**Results:** Thirty-two radiologists (84%) completed the survey. Among the respondents, 56% stated that their participation had a significant impact on their personal reporting practice and 40% reported that it also significantly impacted local practices at their hospital through dissemination among their peers. Reported effects included greater adoption of structured reporting templates, more frequent use of diffusion-weighted imaging for response evaluation, clinical implementation of the sigmoid take-off as a landmark to differentiate between rectal and sigmoid cancers, and influence on discussions about organ preservation in local multidisciplinary team meetings. The vast majority of respondents rated their overall experience with the web platform as either positive (28%) or highly positive (69%).
**Conclusion:** This survey demonstrates that participation in multi-reader imaging studies using web-based tools can influence local diagnostic practices, highlighting its potential not only as a means to support large-scale research but also to promote broader dissemination and clinical implementation.

### SS 8.2

### Added value and clinical impact of second-opinion subspecialist radiologist interpretation of baseline rectal MRI in patients with rectal cancer

J. Golia Pernicka[1], T. Kim[1], S. Nougaret[2], D. Bates[1], M. El Homsi[1], L.J. Fuqua[1], N. Gangai[1], N. Horvat[3], E. Pappou[1], V. Paroder[1], M. Widmar[1], I. Petkovska[1], M.J. Gollub[1]; *[1]New York, NY/US, [2]Montpellier/FR, [3]Rochester, MN/US*

**Purpose:** To determine disagreements between original interpretations and subspecialist radiologists' reinterpretations of baseline staging rectal MRI exams, and their potential impact on patient care in rectal adenocarcinoma patients.
**Material and Methods:** A retrospective analysis of 461 rectal MRIs performed outside our institution (OSHR) and reviewed by eight subspecialist GI radiologists (SECR) was conducted. OSHR and SECR were compared side-by-side and categorized as follows: (1) minor disagreements (insignificant; unlikely to alter treatment, e.g., T3a vs. T3b) and (2) major disagreements (significant; may alter treatment, e.g., overlooked lateral lymph node or T4b disease). Two colorectal surgeons, blinded to the report origins (OSHR vs. SECR), reviewed cases of major disagreement to evaluate their theoretical impact on patient management and rated their confidence level of the OSHR and SECR on a five-point Likert scale (1 = lowest confidence).
**Results:** Disagreements occurred in 248 cases (53.7%), with 140 (30.4%) classified as major (56% of all disagreements). Key discrepancies included missing OSHR details, such as distance to anal verge (182 OSHR vs. 16 SECR) and T3 subclassification (157 OSHR vs. 7 SECR). Among the 140 major disagreements, SECR would have influenced management in 36 patients (25.7%) for surgeon 1 and 64 patients (45.7%) for surgeon 2. Confidence was significantly higher for SECR ($p < 0.001$), with mean scores of 3.56 vs. 2.69 (surgeon 1) and 3.88 vs. 2.02 (surgeon 2).
**Conclusion:** Second-opinion rectal MRI reviews by subspecialist radiologists may impact patient care and significantly improve the surgeon's confidence for treatment planning.

### SS 8.3

### Accuracy of MRI for rectal tumor localization in relation to the peritoneal reflection

K.A. Almakhalas, S. Alsarhani, K. Tan, G. Chan, T. Yee Liang; *Singapore/SG*

**Purpose:** Accurate determination of rectal tumor location relative to the peritoneal reflection (PR) is essential for treatment planning in rectal cancer. This study assesses the diagnostic accuracy of magnetic resonance imaging (MRI) in identifying tumors as above or below the PR, validated against histopathology.
**Material and Methods:** A retrospective analysis was conducted on 309 patients with rectal adenocarcinoma from two institutions. Preoperative MRI classified tumors as above, below, or straddling the PR. Sensitivity, specificity, accuracy, and agreement with histopathology were calculated using kappa statistics.
**Results:** Of 309 tumors, 62 (20.1%) were located above the PR, 146 (47.2%) below, and 101 (32.7%) straddled the PR. For tumors above the PR, MRI demonstrated a sensitivity of 53% (95% CI: 40.1–66.0%), specificity of 89.5% (95% CI: 85.0–93.0%), with an area under the curve (AUC) of 0.713 (95% CI: 0.66–0.76) and an overall accuracy of 82.2% (95% CI: 77.5–86.3%). For tumors below the PR, MRI sensitivity was 76.7% (95% CI: 69.0–83.3%) and specificity 79.8% (95% CI: 72.8–85.6%), with an AUC of 0.782 (95% CI: 0.73–0.83) and an overall accuracy of 78.3% (95% CI: 73.3–82.8). Straddling tumors showed a specificity of 73.1% (95% CI: 66.5–79.0%), with an AUC of 0.608 (95% CI: 0.55–0.66) and an accuracy of 65.0% (95% CI: 59.4–70.4%). Overall there was moderate agreement between MRI and histopathological findings (kappa = 0.43, 95% CI: 0.31–0.56).
**Conclusion:** MRI provides reliable diagnostic performance for assessing rectal tumor location relative to the peritoneal reflection, particularly for tumors below the PR.

### SS 8.4

### MRI predictors of lymph node involvement in clinical T1/2 rectal cancer

M. Fernandes[1], Y. Mazaheri[1], J.L. Fuqua[1], N. Horvat[2], E. Pappou[1], J. Zheng[1], M. Capanu[1], L.A. Rodriguez[3], M.J. Gollub[1], J.S. Golia Pernicka[1]; *[1]New York, NY/US, [2]Rochester, MN/US, [3]Ann Arbor, MI/US*

**Purpose:** To investigate if quantitative Diffusion-weighted imaging (DWI) and qualitative T2-weighted imaging (T2WI) parameters can predict lymph node involvement in clinical early rectal cancer (ERC).
**Material and Methods:** This retrospective study included consecutive patients from January 1, 2010 to March 31, 2021, with rectal MRI showing T1 or T2 rectal cancer before undergoing total mesorectal excision without neoadjuvant therapy. Visible mesorectal lymph nodes on DWI were segmented by a junior reader, and apparent diffusion coefficient (ADC) values were extracted. Additionally, the junior reader and a senior reader independently recorded qualitative T2WI parameters of the most suspicious lymph node per patient. Histopathology was the reference standard for malignant (pN+) and benign (pN–) lymph node status.
**Results:** Of 69 patients (37 men and 32 women) (median age, 55 years; [IQR: 48–65.5]), 23 (33%) had pN+ status while 46 (67%) had pN– status. The ADC mean (in $mm^2/s$) of the most suspicious lymph node was not different between the two patient groups (junior reader: 1,087 vs. 926, $p = 0.31$; senior reader: 1.178 vs. 1,086, $p = 0.89$). The Dutch criteria, based on the combination of T2WI size and morphologic parameters, showed better diagnostic performance for the senior vs. junior reader: sensitivity, specificity, positive predictive value, and negative predictive value of 56.5% (95% CI: 34.5–76.8%), 91.3% (95% CI: 79.2–97.6%), 76.5% (95% CI: 50.1–93.2%), and 80.8% (95% CI: 67.5–90.4%), vs. 26.1% (95% CI: 12.2–48.4%), 91.3% (95% CI: 79.2–97.6%), 60% (95% CI: 26.2–97.8%), and 71.2% (95% CI: 57.9–82.2%).
**Conclusion:** ADC values were not associated with lymph node involvement. The Dutch lymph nodes consensus criteria worked well, particularly for the senior reader.

### SS 8.5

### MRI accuracy to predict R1 resection in (y)pt4 rectal cancer: missing abnormalities or missed opportunities?

C. Keshvala, D. Broadhurst, C. Munasinghe, O. Meysner, N. West, J. Tiernan, D. Tolan; *Leeds/UK*

**Purpose:** T4 rectal cancer (RC) has higher rates of R1 resection causing local recurrence and poorer prognosis. Accurate radiological staging and high-quality surgery underpin R0 resection. Quality and accuracy of RC MRI were assessed in elective resection with R1 (y)pT4 pathology to attribute the cause for R1 status.
**Material and Methods:** Elective RC resections for (y)pT4 with R1 status were analysed to evaluate initial staging and post-treatment rectal MRI and post-operative pathology between 2011 and 2021 to determine the cause for R1 resection.
**Results:** 577 cases were identified in total. (y)pT4 was present in 97 cases with R0 resection in 63/97 (65%). 34/67 (y)pT4 R1 RC were identified (38% female, 62% male). RC MRI quality was optimal in 28/34 (82.4%) and sub-optimal in 6/34 (suboptimal coronal plane, n = 5; respiratory artefact, n = 1). 27/34 (79.4%) had matched pathology/radiological staging with radiological understaging as T3 disease in 7/34 (20.6%) (localised tumour perforation, n = 5; peritoneal invasion, n = 2). Radiology predicted T4a/b tumour or MRF involvement (1mm or less) in 32/34 (94%) and understaged MRF (n = 1) and peritoneal involvement (n = 1) in the remainder. MRI correctly identified (y)pT4b status in 100% of cases (22/22). Surgical planes were beyond the mesorectum 18/34 (53%), mesorectal in 5/34 (15%), and suboptimal muscularis propria/intramesorectal plane in 11/34 (32%). R1 resection was related to a single site in 29/34 (85%) (Circumferential Resection Margin/MRF, 24/29) and multiple sites in 5/34 (15%).
**Conclusion:** MRI accurately predicts high-risk (y)pT4 R1 RC. Current surgical approaches must improve to increase frequency of R0 resection and could justify dedicated referral pathways for beyond TME rectal surgery.

### SS 8.6

### Prediction of pathologic complete response in mucinous rectal tumors using quantitative diffusion-weighted imaging-MRI

M. El Homsi[1], Y. Mazaheri[1], J. Kuten[1], M.J. Corines[1], N. Horvat[1], A. Baheti[2], S.P. Sheedy[3], S. Lee[4], S. Nougaret[5], R. Ernst[6], C.C. Moreno[7], E. Korngold[8], S. Lobaugh[1], M. Capanu[1], M.J. Gollub[1]; *[1]New York, NY/US, [2]Mumbai/IN, [3]Rochester, MN/US, [4]Orange, CA/US, [5]Montpellier/FR, [6]Texas, TX/US, [7]Georgia, GA/US, [8]Portland, OR/US*

**Purpose:** To explore diffusion-weighted imaging-MRI and the value of apparent diffusion coefficients (ADC) in predicting pathologic complete response (pCR) in mucinous rectal cancer (MRC) after neoadjuvant therapy (nAT).

**Material and Methods:** This institutional review board-approved, consent-waived retrospective pilot study included 150 patients with MRC treated with nAT followed by surgical resection. Three radiologists (with 30 years, 3 years of experience, and a radiology oncology fellow) blinded to pathologic response segmented residual mucinous tumor on diffusion images (b-values 750–1000) using Image J. An experienced Medical Physicist analyzed ADC maps to generate the mean, standard deviation, median, 1st quartile (12.5%), 2nd quartile (25%), 3rd quartile (75%), and 4th quartile of the volume segmented (87.5%). Inter-reader agreement in terms of ADC values was assessed using intra-class correlation (ICC). Wilcoxon rank sum tests were used to examine associations between ADC values and pCR for each reader. The false discovery rate (FDR) correction for multiple testing was used.

**Results:** The included patients had a mean age of 58 ± 14 years and 48/150 (32%) were female. 25 patients (17%) had pCR and 125 (83%) had non-pCR (containing cellular mucin). There was weak to moderate agreement between our readers for all ADC values measurements (ICC between 0.26 and 0.67). There was no statistically significant association between pCR and ADC values for any of the readers (q values between 0.14 and 0.58).

**Conclusion:** We did not observe statistically significant evidence that ADC measurements help with differentiating between pCR compared to non-pCR group in patients with MRC treated with nAT.

### SS 8.7

### MRI radiomics for distinguishing residual adenocarcinoma from acellular mucin in treated rectal cancer

M.J. Corines[1], A. Ibrahim[1], A. Baheti[2], P. Gibbs[1], S.P. Sheedy[3], S. Lee[4], S. Nougaret[5], R. Ernst[6], C.C. Moreno[7], E. Korngold[8], M. Fox[1], J. Miranda[1], S. Carlos Nahas[9], I. Petkovska[1], J. Zheng[1], R. Sosa[1], N. Gangai[1], B. Zhao[1], L.H. Schwartz[1], N. Horvat[1], M.J. Gollub[1]; *[1]New York, NY/US, [2]Mumbai/IN, [3]Rochester, MN/US, [4]Orange, CA/US, [5]Montpellier/FR, [6]Houston, TX/US, [7]Atlanta, GA/US, [8]Portland, OR/US, [9]Sao Paulo/BR*

**Purpose:** To determine if MRI T2-weighted imaging (T2WI) radiomics can accurately distinguish acellular from cellular mucin on rectal MRI after treatment.

**Material and Methods:** This retrospective, multi-institutional study included rectal adenocarcinoma patients having mucin on restaging MRI from March 2012 to January 2020. Radiologists segmented 3-mm-thick T2WI. Data were split into training (n = 122), validation (n = 40), and test (n = 42) sets. Hyper-parameter grid-search was performed to develop the model. Highly correlated features and those with near-zero variance were removed. Recursive features elimination selected five features. Xgboost algorithm was used to build the model. Sensitivity, specificity, positive predictive value (PPV), negative predictive value (NPV), and area under the curve (AUC) were evaluated on the test set. Histopathology was the reference standard. Two radiologists independently reviewed 42 patients using a unique 5-point Likert scale for the probability of cellular mucin. AUC of the radiomics model was compared to that of the human Likert scale using DeLong–Delong test.

**Results:** Of 204 patients (mean age, 56.6 ± 13.3 years; 129 men and 75 women), 39/204 (19%) had acellular mucin, whereas 165/204 (81%) had residual cellular mucin. The radiomics model demonstrated a sensitivity of 90% (95% CI: 74%, 97%), specificity of 83% (95% CI: 55, 95%), PPV of 93% (95% CI: 78%, 98%), NPV of 77% (95% CI: 50%, 92%), and AUC of 0.84 (95% CI: 0.65, 0.99) on the test set, whereas the human readers showed inferior sensitivities of 47% (95% CI: 28%, 66%) and 50% (95% CI: 31%, 69%) and AUCs of 0.58 (95% CI: 0.41, 0.74) and 0.73 (95% CI: 0.57, 0.88).

**Conclusion:** The developed model predicts cellular mucin post-neoadjuvant chemoradiation with higher sensitivity than human readers.

### SS 8.9

### Conveying a clear message: MRI reporting to select rectal cancer patients for organ-preservation— an analysis of the Dutch wait-and-see registry

N. Tilman, P. Custers, D. Lambregts, B.A. Grotenhuis, G. Beets, R. Beets-Tan, M. Maas; *Amsterdam/NL*

**Purpose:** MRI plays an important role in selecting rectal cancer patients for organ-preservation after neoadjuvant therapy. Key requirement of a good-quality MRI report to guide clinical decision-making in the setting of organ-preservation is that radiologists provide a clear description of the degree of response. This study aimed to retrospectively assess the quality of MRI reporting in the Dutch Wait-and-See registry.

**Material and Methods:** The Dutch Wait-and-See registry, a multicentre cohort study, includes rectal cancer patients with complete response after chemoradiotherapy that follow wait-and-see policy. In this registry, diagnostic data were reviewed upon inclusion by a dedicated expert team at the principal investigator site. Two expert radiologists evaluated whether the original reports from the referring centres clearly indicated the degree of response, categorized it into 3 categories: complete, partial/near-complete, or residual tumour, or used alternative scoring systems, such as mrTRG.

**Results:** 287 reports were evaluated. An unclear degree of response, e.g. ‘good response after chemoradiotherapy’, was observed in 19% of the conclusions. In 25% of conclusions, the three-way response categorization was applied. Alternative ways to describe complete response, e.g. ‘yT0N0’ or ‘no residual tumour’, were used in 43% of the conclusions, implicitly directing towards organ-preservation. 2% of conclusions explicitly directed towards organ-preservation, and 6% suggested correlation of results with endoscopy.

**Conclusion:** In 19% of restaging MRI reports in patients following a wait-and-see policy, degree of response was unclear from the conclusion. This may lead to underselection of patients for organ-preservation. Improvements should be made in reporting the degree of response at restaging: e.g. through teaching and better incorporation in (inter)national guidelines.

### SS 8.10

### MRI can accurately stage local regrowth in rectal cancer during watchful waiting prior to surgery

X. Ou, K. Chupetlovska, B. Geubels, D. Lambregts, B. Grotenhuis, G. Beets, R. Beets-Tan, M. Maas; *Amsterdam/NL*

**Purpose:** Local regrowth occurs in approximately 25% of rectal cancer patients during watchful waiting, often requiring salvage surgery. This study evaluates the diagnostic accuracy of preoperative MRI for ycTN-staging of local regrowth, to guide treatment decisions between local excision (LE) and total mesorectal excision (TME).

**Material and Methods:** Thirty-five patients with local regrowth undergoing salvage surgery (LE and/or TME) were included. Preoperative ycTN stages from MRI were independently assessed by an expert and general abdominal radiologist (non-expert) and compared to histopathology. Patients undergoing LE were included if follow-up >2 years showed no nodal recurrence (reference standard for yN0). yT-stage was dichotomized into yT0-2 and yT3-4, and yN-stage into yN0 and yN+ (yN1+yN2). Diagnostic performance and interobserver agreement were analyzed.

**Results:** Among 35 patients, 23 had ypT0-2, 12 had ypT3-4; 28 were ypN0, and 7 were ypN+. For yT-staging, the expert showed sensitivity/specificity of 92%/83% and the non-expert 50%/91%. Positive predictive values (PPVs) were 73% (expert) and 75% (non-expert), while negative predictive values (NPVs) were 95% (expert) and 78% (non-expert). For yN-staging, sensitivity was 57% for both readers and specificity was 100% (expert) and 96% (non-expert). PPVs were 100% (expert) and 80% (non-expert), while NPVs were 90% for both. Interobserver agreement was $\kappa = 0.6$ for ycT staging and $\kappa = 0.9$ for ycN staging.

**Conclusion:** Preoperative MRI shows high accuracy and moderate-good reproducibility to stage rectal cancer regrowth during watchful waiting. Results for yT-staging were better for the expert reader, particularly for yT0-T2. Agreement for yN-staging is high, but understaging is uncommon.

**11:00 – 12:30** **E 105/106**

*Scientific Session SS 9*

### Advances in HPB imaging

### SS 9.1

### Quantitative MRI parameters in patients with Type 1, Type 2 diabetes, and latent autoimmune diabetes in adults (LADA): a correlation prospective study

B. Masci, S. Nardacci, M. Zerunian, A. Zurru, E. Maddaloni, R. Buzzetti, D. De Santis, D. Caruso, A. Laghi; *Rome/IT*

**Purpose:** To identify potential differences in quantitative magnetic resonance imaging (MRI) in patients with type 1 diabetes (T1D), type 2 diabetes (T2D), and LADA.

**Material and Methods:** A total of 30 patients with T1D, T2D, and LADA, aged 50–60, were prospectively enrolled from September 2023 to September 2024. Each patient underwent a 1.5T upper abdomen MRI examination, including axial proton density fat fraction (PDFF) and magnetic resonance elastography (MRE). Two radiologists performed quantitative image analysis. Liver PDFF (L-PDFF), pancreatic PDFF (Pa-PDFF), liver stiffness, and pancreatic volume (Pa-Vol) were calculated. Manual segmentation of PDFF images was performed using a dedicated software (3DSlicer v4.10.2) to determine Pa-Vol. One-way ANOVA test and Spearman's rank coefficient were used to assess differences and correlations between parameters; a p-value $< 0.05$ was considered statistically significant.

**Results:** 8 patients (26%) exhibited L-PDFF values > 5.5%, indicating hepatic steatosis; 16 patients (53%) showed liver stiffness values > 2.4 kPa, compatible with inflammation/low-grade fibrosis. Additionally, 10 patients (33%) of the cohort had reduced Pa-Vol. Significant differences in LPDFF were observed between T2D and T1D patients ($p = 0.015$). No significant differences in Pa-PDFF were found across the groups ($p = 0.304$). In T1D patients, LPDFF significantly correlated with Pa-PDFF ($p < 0.0001$), but this was not observed in T2D or LADA patients. Subgroup analysis showed significant differences in Pa-Vol between T2D (50.9 ± 2.76 $cm^3$), LADA (45 ± 2.2 $cm^3$), and T1D (44.1 ± 4.17 $cm^3$) ($p = 0.0003$); significant differences in Pa-Vol were mainly observed between T2D and LADA ($p < 0.05$).

**Conclusion:** This study highlights significant variations in Pa-Vol and L-PDFF across diabetes types, suggesting MRI as a non-invasive method to evaluate diabetic patients and monitor therapy progression.

### SS 9.2

### Prevalence and prognostic impact of sarcopenia in patients with porto-sinusoidal vascular disorder

K. Lampichler[1], J. Ferrusquía-Acosta[2], A. Darnell[3], B. Procopet[4], S. Gioia[5], X. Luo[6], F. Ravaioli[7], E. Dajti[7], H. Giudicelli[8], C. Gayat[9], L. Elkrief[9], A. Valsan[10], C. González-Alayon[11], F. Schepis[12], D. Saltini[12], L. Turco[7], Ö. Koc[13], M. García-Calonge[14], E. Llop[15], N. Pugliese[16], L. Kernanet[17], P.-E. Rautou[17], T. Reiberger[1], V. Hernández-Gea[3], J. García-Pagán[3], B. Scheiner[1], L. Balcar[1], A. Baiges[3], M. Ronot[17]; *[1]Vienna/AT, [2]Sabadell/ES, [3]Barcelona/ES, [4]Cluj-Napoca/RO, [5]Rome/IT, [6]Sichuan/CN, [7]Bologna/IT, [8]Paris/FR, [9]Tours/FR, [10]Kochi/IN, [11]Tenerife/ES, [12]Modena/IT, [13]Maastricht/NL, [14]Oviedo/ES, [15]Majadahonda/ES, [16]Milan/IT, [17]Clichy/FR*

**Purpose:** Despite being a recognized risk factor in patients with cirrhosis, the role of sarcopenia in patients with porto-sinusoidal vascular disorder (PSVD) remains unclear. This study investigates the prevalence and prognostic impact of sarcopenia in patients with PSVD.

**Material and Methods:**
A retrospective, multicentre, observational study was conducted using data from two European tertiary care centres. Sarcopenia prevalence was assessed in PSVD patients (n = 197; derivation cohort, mean 49 ± 16 years; 57% male) and compared to advanced chronic liver disease (ACLD) patients (n = 720, mean 55 ± 12 years, 61% males) using propensity score matching for sex, ascites, and Child-Turcotte-Pugh score (CTP). Sarcopenia was defined by transversal psoas muscle thickness (TPMT; females: <8 mm/m, males: <12 mm/m) at the level of L3 on cross-sectional imaging. A competing risk regression analysis identified factors associated with first/further hepatic decompensation and liver-related death. Results were validated in an independent PSVD cohort derived from 18 VALDIG centres (n = 409).

**Results:** Sarcopenia prevalence in the PSVD training cohort was comparable to the propensity score matched ACLD cohort (18% vs 16%; $p = 0.592$) and the PSVD validation cohort (19%; $p = 0.958$). The intraclass correlation for TPMT measurements in a subset of patients (n = 126) was excellent (0.919). Multivariable competing risk regression analysis identified age, history of decompensation, CTP, creatinine, and sarcopenia as independently associated with first/further decompensation and liver-related death in the overall PSVD cohort (sarcopenia: aSHR: 1.48 [95% CI, 1.01–2.17], $p = 0.045$), with a more pronounced impact of sarcopenia among previously decompensated patients.

**Conclusion:** Sarcopenia prevalence is comparable in PSVD and ACLD patients matched for severity of liver dysfunction and represents an independent risk factor of poor outcome.

### SS 9.3

### Characterisation and evolution of CT-based imaging findings in patients with porto-sinusoidal vascular disorder

K. Lampichler[1], L. Kernanet[2], R. Sartoris[2], C. Watzenboeck[1], L. Moga[2], P.-E. Rautou[2], M. Ronot[2]; [1]Vienna/AT, [2]Clichy/FR

**Purpose:** The clinical course of porto-sinusoidal vascular disorder (PSVD) is less severe than that of cirrhosis but complications of portal hypertension or splanchnic vein thrombosis can occur. We evaluated changes in imaging features of PSVD over time in a large patient cohort.

**Material and Methods:** Retrospective single-center study includes patients with biopsy-proven PSVD and two contrast-enhanced abdominal CT scans ≥12 months apart. Two readers independently evaluated several imaging features, including spleen size, liver morphology, ascites, splanchnic vein thrombosis, spontaneous porto-systemic shunts (SPSS), and FNH-like lesions.

**Results:** 134 patients were included (mean 48±15.1 years, 54% males). Median follow-up was 5.7 years (IQR 2.05–8.6 years). Most common causes of PSVD included autoimmune/inflammatory diseases (38.8%), infectious diseases (16.4%), and hematological disorders (7.4%). Splanchnic vein thrombosis and/or mural calcifications increased significantly over time (thrombosis: 11.9% vs. 12.7%, mural calcifications: 5.2% vs. 6%, combination: 2.2% vs. 11.2%, p = 0.02). Intrahepatic portal vein abnormalities (23.1% vs. 35.8%, p = 0.03) and SPSS also increased significantly (overall: 77.6% vs. 89.6%, p = 0.01; large SPSS: 25.4% vs. 43.3%, p = 0.003). Abnormal liver morphology was already highly prevalent initially (94% overall; 88.8% segment I hypertrophy, 32.8% segment IV hypertrophy, 8.2% atrophy/hypertrophy complex) and did not change significantly (p = 0.57). Spleen size (mean 15.2 ± 13.7 vs. 15.21 ± 3.7 cm, p = 0.89), ascites (32.1% vs. 35.8%, p = 0.40) and FNH-like lesions (14.6% vs. 23.3%, p = 0.23) did not change significantly.

**Conclusion:** In patients with PSVD, the presence of splanchnic vein thrombosis and intrahepatic portal vein abnormalities increases over time, while liver morphology, spleen size, and ascites remain largely unchanged.

### SS 9.4

### Hepatic atypical hemangiomas: navigating diagnostic uncertainty

S. Tamir[1], R. Kedem Mashraki[1], T. Krutik[2], G. Ben Arie[2]; [1]Petah Tikvah/IL, [2]Beer Sheba/IL

**Purpose:** The term "atypical hemangioma" lacks precise definition, potentially leading to management challenges. We aimed to explore the characteristics of lesions interpreted as atypical hemangiomas, evaluate whether the use of this term is associated with unnecessary workup, and whether it is associated with under-diagnosis of malignant lesions.

**Material and Methods:** This retrospective study reviewed radiology reports from two hospitals between 2013 and 2023 that contained the term `atypical hemangioma'. Two radiologists reviewed the images independently and documented lesion characteristics based on standardized criteria. Ground truth categorization was established using additional imaging, follow-up data, and pathological correlation when available.

**Results:** Of 327 cases, 305 (93.3%) were benign, while 22 (6.7%) were malignant. Significant differences were observed between benign and malignant lesions' characteristics. Malignant lesions were larger, more frequently had ill-defined borders (50%vs.18.1%/54.5%vs.17.9%, malignant vs. benign data of both readers), early heterogeneous enhancement (46.2%vs.9.7%/46.2%vs.9.3%, malignant vs. benign data of both readers), and restricted diffusion on MRI (100%vs.17.6%/75%vs.7.8%, malignant vs. benign data of both readers).

**Conclusion:** This study highlights the ambiguity of the term "atypical hemangioma" which may be associated with unnecessary workup of benign lesions or delayed diagnosis of malignant lesions. We suggest that in the presence of atypical features, the report should mention malignancy potential and recommend appropriate workup strategy. Once a diagnosis of hemangioma is confidently made, we suggest using the term "hemangioma with atypical features". This approach may help optimize patient management, reduce unnecessary procedures and allow timely diagnosis of malignancy.

### SS 9.5

### CT and MRI of hepatic small vessel neoplasm: a new vascular tumor entity

R. Aldhaheri[1], A. Laurent-Bellue[2], A. Beaufrère[3], C. Desterke[1], A. Paisant[4], I. Bricault[5], P. Borde[6], G. Simon[7], C. Prud'Homme[1], V. Vilgrain[3], M. Lewin[1]; [1]Villejuif/FR, [2]Le Kremlin-Bicêtre/FR, [3]Clichy/FR, [4]Angers/FR, [5]Grenoble/FR, [6]Lille/FR, [7]Besancon/FR

**Purpose:** The aim of this study was to describe the radiological characteristics on dynamic CT and MRI of pathologically confirmed Hepatic small vessel neoplasm (HSVN).

**Material and Methods:** In a retrospective multicentric study, patients diagnosed between 2014 and 2024 with pathologically proven HSVN and cross-sectional imaging (CT/MRI) were included. Categorical variables and qualitative imaging features are described. The group of patients with a typical imaging HSVN aspect according to previous literature was compared with those with an atypical aspect. Univariable analysis and binomial multivariable model were used for the typical and atypical HSVN aspect outcome.

**Results:** 27 patients were included (19 men, median age, 56 years [IQR, 19–76]). Tumor median size at diagnosis was 21 mm [IQR, 10–80]. Lesions were single in 74% (20/27) and diffuse in 26% (7/27) of patients. In 63% (17/27) of patients, the typical HSVN aspect showed lobulated lesion (P = 0.002), marked hypersignal on T2 (P = 0.004), high apparent diffusion coefficient (ADC) (tumor mean ADCs, $2 \times 10^{-3} \pm 0.3$ $mm^2$/sec) (P = 0.01), and an arterial thick continuous peripheral enhancement as a "flower petal shape" (P = 0.039) with portal and late filling after contrast injection. Atypical HSVN aspect found in 37% (10/27) of patients was the presence of homogeneous arterial enhancement persisting at portal and delayed phase. At a median follow-up of 18 months, a tumor growth was observed in 44% (8/18) patients with a higher rate of progression in patients with typical compared to atypical HSVN aspect (P = 0.013).

**Conclusion:** The imaging typical aspect of HSVNs combines lobulated peripheral hyper-vascularization with a marked hypersignal on T2-weighted imaging and high ADC, enabling them to be diagnosed.

### SS 9.6

### Improved detection of focal liver lesions with manganese-based contrast agent in patients with severe kidney impairment: evidence from the SPARKLE study

V. Lucidi[1], C. Mosconi[1], M. Renzulli[1], D. Conte[1], N. Hettiarachchige[2], D. Geisel[3], A. Norlin[4], N. Kartalis[5], T. Brismar[5]; [1]Bologna/IT, [2]Copenhagen/DK, [3]Berlin/DE, [4]Malmo/SE, [5]Stockholm/SE

**Purpose:** ACE-MBCA (Ascelia Pharma Manganese-Based Contrast Agent, proposed trade name Orviglance) is an oral manganese-based liver-specific MRI contrast agent developed for visualizing and detecting focal liver lesions (FLLs).

**Material and Methods:** In the SPARKLE study, patients (N = 85) with known or suspected FLLs and severe renal impairment received a liver MRI before and 4 ± 1 h after the administration of ACE-MBCA (800 mg). The secondary efficacy analysis, lesion detection, was evaluated by three independent readers (R1, R2, R3), comparing unenhanced MRI (UMRI) and combined MRI (CMRI) and proportion of patients with new lesions per lesion-size category.

**Results:** The 3 readers consistently found more lesions using CMRI compared to UMRI. The average number of lesions (mean ± SD) for each reader was as follows: R1 - UMRI = 3.5 ± 5.18, CMRI = 4.2 ± 5.19, R2: UMRI = 3.1 ± 5.07, CMRI = 3.2 ± 4.85, R3: UMRI = 4.1 ± 5.58, CMRI = 4.3 ± 5.59. Additionally, the readers detected at least one new lesion with CMRI in 51.8% (R1), 38.8% (R2), and 40.0% (R3) of patients. Finally, all three readers found small lesions (< 1 cm) in more patients with CMRI than with UMRI. Out of 85 patients, the number of patients with small lesions detected was as follows: R1 - UMRI = 16, CMRI = 25, R2 - UMRI = 13, CMRI = 18, R3 - UMRI = 28, CMRI = 36.

**Conclusion:** Contrast enhancement with ACE-MBCA increases the detection of small size lesion providing an important imaging support in the early management and prognosis of patients with severe kidney impairment and liver disease.

### SS 9.7

### Imaging features, differential diagnosis and management of solitary necrotic nodule of the liver: a case series

L. Asmundo, L. Giaccardi, A. Soro, T. Cascella, A. Casale, M. Vaiani*; Milan/IT*

**Purpose:** Solitary necrotic nodule of the liver (SNNL) is a rare benign hepatic lesion frequently mistaken for malignancy, often resulting in unnecessary invasive procedures. This study aimed to assess the imaging features, clinical management, and outcomes of SNNL.
**Material and Methods:** This retrospective case series was conducted on patients diagnosed with SNNL at a tertiary center. Collected data included demographics, imaging studies, features and follow-up outcomes. Imaging studies were reviewed by an experienced radiologist, with histological examination or long-term imaging serving as the reference standard.
**Results:** Thirteen patients (54% female, median age 54 years) were included. MRI was the primary imaging modality, performed 3–5 times over a median follow-up of 25 months. SNNLs commonly appeared as necrotic cores with fibrotic capsules, often resembling biliary dilatation. On T1-weighted imaging, the core was hypointense in 84.6% of cases and isointense in 53.8%. On T2-weighted imaging, the core was hypointense in 61.5% and isointense in 38.5%. Diffusion-weighted imaging showed no restricted diffusion. Follow-up imaging demonstrated a reduction in nodule size in 92.3% of cases, with increasing calcification noted on CT (38.5% to 69.2%).
**Conclusion:** SNNL presents diagnostic challenges due to its similarity to aggressive hepatic lesions such as cholangiocarcinoma. However, characteristic MRI and CT findings can aid accurate diagnosis, avoiding unnecessary biopsies or surgeries. Long-term imaging follow-up supports the benign nature of SNNL, reinforcing the importance of non-invasive management strategies.

### SS 9.8

### The Liver Artificial Intelligence (LAI) Consortium: a benchmark dataset and optimised machine learning methods for MRI-based diagnosis of solid-appearing liver lesions

F. Hartmann[1], R. Niemantsverdriet[1], M. Veenstra[1], M. Doukas[1], R. Dwarkasing[1], V. Vilgrain[2], M. Ronot[2], B. Taouli[3], R. Miclea[4], G. Brancatelli[5], M. Bali[6], J. Prince[7], R. de Haas[8], V. de Meijer[8], O. van Delden[9], J. Erdmann[9], N. Kutaiba[10], M. Bobowicz[11], L. Faggioni[12], K. Lekadir[13], K. Fowler[14], F. Illerstam[15], S. Klein[1], M. Starmans[1], M. Thomeer[1], -.-. LAI Consortium[1]*;*
*[1]Rotterdam/NL, [2]Clichy/FR, [3]New York, NY/US, [4]Maastricht/NL, [5]Palermo/IT, [6]Brussels/BE, [7]Utrecht/NL, [8]Groningen/NL, [9]Amsterdam/NL, [10]Melbourne/AU, [11]Gdańsk/PL, [12]Pisa/IT, [13]Barcelona/ES, [14]San Diego, CA/US, [15]Stockholm/SE*

**Purpose:** The diagnosis of solid-appearing liver lesions on MRI is critical for determining treatment and prognosis. The complexity and variety of phenotypes and imaging across patients often make this challenging, subjective, and time-consuming. To fast-forward the use of artificial intelligence (AI) for liver imaging, the Liver Artificial Intelligence (LAI) consortium is constructing a large-scale benchmark dataset, developing optimized machine learning models, and rigorously validating the models in realistic clinical settings. All consortium members are available at https://lai-consortium.org/.
**Material and Methods:** Twelve hospitals across three continents are retrospectively collecting routine MRI scans of pathologically confirmed solid-appearing liver lesions. The dataset will contain both common and rare types of malignant (primary/metastatic) and benign lesions, with multiple MRI sequences. The project has no restrictions on age, sex, or underlying liver disease and aims to collect data from more than 3,000 patients, making it the largest publicly available MRI dataset of solid-appearing liver lesions.
**Results:** To date, the LAI dataset includes 486 patients, exceeding 2,500 MRI scans. These include 156 patients with hepatocellular carcinoma, 65 with intrahepatic cholangiocarcinoma, 139 with hepatocellular adenoma, and 126 with focal nodular hyperplasia. Each patient has minimally one T2-weighted MRI scan, with many also having dynamic contrast-enhanced T1-weighted scans covering pre-contrast, arterial, portal venous, delayed, or hepatobiliary phases.
**Conclusion:** The LAI dataset will serve as a key resource for the development and evaluation of AI algorithms designed to automate the diagnosis of solid-appearing liver lesions using MRI. This dataset and the resulting algorithms have the potential to aid radiologists in their decision-making and assist in patient referrals to specialized centres.

### SS 9.9

### Impact of a new generation dual-energy CT on the quality of liver imaging, reduction of iodine contrast media consumption and radiation dose: a monocentric experience

I. Capitoni, G. Bagnacci, A. Perrella, G. Daccordi, F. Lazzeretti, N. Di Meglio, M. Mazzei*; Siena/IT*

**Purpose:** The aim of this study is to demonstrate the impact of a new generation of dual-energy CT (DECT) with Smart mAs (SmAs) modulation and Deep Learning Iterative Reconstruction (DLIR) on reducing iodine contrast media (CM) consumption and radiation dose (RD), while improving Liver Image Quality (LIQ).
**Material and Methods:** Consecutive multi-phase whole-body scans were acquired from September 2022 to December 2023 with old generation DECT and from January to November 2024 with NGCT. Iodine Contrast administration data [total iodine load (TIL) and iodine-to-weight ratio] and radiation dose delivery data [dose-length product and computed tomography dose index (CTDI)] were reported. LIQ was assessed using contrast-to-noise ratio (CNR) and signal-to-noise ratio (SNR) acquired via region of interest placed on liver and paravertebral muscles, comparing polychromatic scans from the old DECT with multiple reconstructions from the new DECT. The images were given anonymously to expert readers for a subjective assessment.
**Results:** Comparing 292 examinations from both DECTs, we obtained a TIL reduction of 18.01%. CTDI reduction was observed in all phases: 18% in basal, 54% in arterial, 31% in portal and 51% in late phase. Subjective images' assessment showed improved diagnostic value. For objective assessment, we found an improvement in CNR (35.71% and 50.08%, respectively) and SNR (32.04% and 39.98%, respectively) for both 120 kVp and 40 KeV reconstructions.
**Conclusion:** The integration of DLIR and SmAs has shown a great impact on CM consumption and RD delivery compared to old generation exams with an increase in exam quality.

### SS 9.10

### Intraindividual comparison of half-dose gadopiclenol and standard dose of gadobenate dimeglumine for abdominal MRI

A. Del Gaudio[1], D. Kruse[1], K. Kalisz[1], L. Lofino[1], D. De Santis[2], R. Gupta[1], F. Ria[1], D. Marin[3]*;*
*[1]Durham, NC, NC/US, [2]Rome/IT, [3]Durham, NC/US*

**Purpose:** To intraindividually compare image quality and lesion conspicuity of abdominal MRI using gadopiclenol at 0.05 mmol/kg and gadobenate dimeglumine (Gd-BOPTA) at 0.1 mmol/kg.
**Material and Methods:** Between September 2023 and March 2024, patients who underwent two clinically indicated contrast-enhanced abdominal MRIs within 12 months using gadopiclenol and Gd-BOPTA on the same scanner were retrospectively enrolled. Signal intensities of abdominal organs, vessels, and lesions (liver, pancreas, kidneys) were manually measured across unenhanced, late arterial, venous, and equilibrium phases. Signal-to-noise ratio (SNR), contrast-to-noise ratio (CNR), and magnitude of contrast enhancement (ΔE) were calculated. Percentage enhancement (%E) was assessed for lesions, and subjective image quality was rated using a 5-point Likert scale. Lesion characteristics such as border delineation and internal enhancement were also evaluated. Paired t-tests and Wilcoxon tests were used for statistical analysis.
**Results:** One hundred subjects (64 years ± 14; 55 men) were selected and a total of 21 abdominal lesions were included. Compared to Gd-BOPTA, gadopiclenol yielded significantly higher CNR and SNR for pancreas, porta, and kidney in the late arterial phase ($p \leq .040$). No significant differences in CNR and SNR were observed between gadopiclenol and Gd-BOPTA across all organs in the portal venous and equilibrium phases. Gadopiclenol showed significantly higher pancreatic ΔE in all contrast-enhanced phases ($p \leq .049$) compared to Gd-BOPTA. The %E of abdominal lesions was comparable between gadopiclenol and Gd-BOPTA for all contrast-enhanced phases ($p \geq .100$). No significant differences were observed between gadopiclenol and Gd-BOPTA in readers' perception of image quality and lesions' characteristics.
**Conclusion:** Gadopiclenol at 0.05 mmol/kg provides comparable image quality to Gd-BOPTA at 0.1 mmol/kg, with improved pancreatic enhancement.

**14:30 – 16:00** **E 103/104**

*Scientific Session SS 10*

### Chronic liver disease: fibrosis, cirrhosis, portal hypertension

### SS 10.1

### Functional liver imaging score: a biomarker for acute-on-chronic liver failure

L. Beer, S. Pötter-Lang, N. Bastati-Huber, L. Balcar, A. Kristic, M. Mandorfer, A. Ba-Ssalamah*; Vienna/AT*

**Purpose:** Functional Liver Imaging Score (FLIS) derived from gadoxetic acid-enhanced MRI predicts hepatic decompensation and liver-related mortality in patients with advanced chronic liver disease (ACLD). The aim of this study was to investigate whether semiquantitative FLIS and quantitative imaging parameters, namely, the relative liver enhancement (RLE), relative enhancement ratio of the biliary system (REB), and liver-to-portal vein contrast ratio (LPC), enable prediction of acute-on-chronic liver failure (ACLF) in patients with acute decompensation (AD) of cirrhosis.
**Material and Methods:** We included 322 ACLD patients with GA-MRI-derived, semi-quantitative FLIS, in whom the RLE, REB, and LPC were also computed by two independent, experienced radiologists. Patients were stratified into stable ACLD (including compensated or nonacutely decompensated) and acutely decompensated (AD; i.e. non-elective hospitalized for cirrhosis complications) patients. The predictive values of semi-quantitative FLIS and quantitative GA-MRI parameters for ACLF development and liver-related mortality were investigated by Cox regression analyses.
**Results:** The FLIS was lower in AD (vs. stable ACLD) patients. Furthermore, low FLIS was an independent risk factor for ACLF development/liver-related death in AD patients (adjusted hazard ratio [aHR]: 2.26; 95% CI: 1.08–4.71; P = 0.03), but also in clinically stable ACLD patients (aHR: 2.35; 95% CI: 1.21–4.55; P = 0.01). Conversely, while RLE, REB, and LPC distinguished between AD and clinically stable ACLD patients (P < 0.001), they failed to predict ACLF/liver-related death.
**Conclusion:** The FLIS is a simple but versatile prognostic imaging biomarker in ACLD patients, as it can predict their risk for ACLF and liver-related death.

### SS 10.2

### Diagnostic accuracy of magnetic resonance and shear wave elastography in advanced liver fibrosis detection for metabolic dysfunction-associated steatotic liver disease patients

G. Mattana, A. Blandino, R. Cannella, P. Alba, A. Tulone, S. Petta, T. Bartolotta*; Palermo/IT*

**Purpose:** To compare the diagnostic performance of magnetic resonance elastography (MRE) and shear wave elastography (2D-SWE) for detecting advanced liver fibrosis (F ≥ 3) in patients with metabolic dysfunction-associated steatotic liver disease (MASLD).
**Material and Methods:** Fifty-nine adult patients (median age 60.0 years; range 55.0–70.0) with MALSD, including 40 males and 19 females, were recruited. All participants underwent MRI on a 3T scanner using a fast field echo-MRE sequence and 2D-SWE on the same day. Histopathological and clinical data were used for the reference standard to grade the presence of fibrosis and cirrhosis. Diagnostic performance was assessed using the area under the receiver operating characteristic curve (AUC), with optimal cutoffs determined by the Youden index and it was compared by the DeLong test.
**Results:** Among 59 patients enrolled, 10 (16.9%) were classified as F0-F2 and 49 (83.1%) as F3-F4. Valid MRE measurements were available for 53 patients (F0-F2: n = 8; F3-F4: n = 45) and in all patients with 2D-SWE. Significant differences were found between F0-F2 and F3-F4 groups in both MRE (p < 0.001) and 2D-SWE (p < 0.001). MRE achieved an AUC of 0.878 (95% CI: 0.759–0.952) with a sensitivity of 80% and specificity of 100% using the cutoff of >3.02 kPa. 2D-SWE showed a slightly higher AUC of 0.898 (95% CI: 0.791–0.962) with a sensitivity of 73.5% and specificity of 100% using the cutoff of >6.3 kPa, with no significant difference compared to MRE (p = 0.932).
**Conclusion:** Both MRE and 2D-SWE are reliable, non-invasive methods for diagnosing advanced fibrosis in MASLD, offering valuable alternatives to liver biopsy.

### SS 10.3

### Automated artificial intelligence-enabled CT biomarkers for population-based opportunistic detection of patients at risk for unsuspected advanced hepatic fibrosis and cirrhosis

M.G. Lubner[1], D. Tran[1], J. Garrett[1], R.M. Summers[3], P.J. Pickhardt[1]*; [1]Madison, WI/US, [3]Bethesda, MD/US*

**Purpose:** To determine the utility of opportunistic automated artificial intelligence (AI)-enabled CT biomarkers in assessing risk for hepatic fibrosis and cirrhosis.
**Material and Methods:** AI-enabled CT tools that automatically assess the liver segmental volume ratio (LSVR; ratio of Couinaud segments I-III/IV-VIII) and splenic volume were applied to a heterogeneous cohort of 30940 adults (mean age 51.5 years; 14459 men, 16481 women) who underwent abdominal CT scans for any reason and also had an available retrospective FIB-4 score (consisting of patient age, serum AST, ALT, platelets) within 1 year of CT. Previously established high-risk thresholds of 0.347 for LSVR and 315 ml for splenic volume were applied, and the resulting cohorts were analyzed according to FIB-4 levels.
**Results:** Among the 3052 patients (9.9%) who had both elevated LSVR and splenic volume at CT, the mean FIB-4 score was 3.30, whereas among the 14812 patients (47.9%) below both the LSVR and splenic volume thresholds, the mean FIB-4 score was 1.12. For the 5870 patients (19.0%) with elevated LSVR but not splenic volume, mean FIB-4 score was 1.25. For the 7206 patients (23.3%) with elevated splenic volume but not LSVR, mean FIB-4 score was 1.74. Among patients below the LSVR and splenic volume thresholds, 4.8% had a FIB-4 score > 2.67 versus 41.0% among patients above both CT-based thresholds.
**Conclusion:** AI-enabled automated hepatosplenic CT biomarkers can opportunistically identify patients at risk for underlying advanced hepatic fibrosis and cirrhosis, regardless of the clinical indication for imaging.

### SS 10.4

### Assessing the L distance and caudate right lobe ratios in enhanced CT for fibrosis staging

S.N. Emir, G. Karamustafao, F. Kulali*; Istanbul/TR*

**Purpose:** This study aimed to assess the effectiveness of two distance ratio-based methods, the L distance ratio and the caudate right lobe ratio, in staging fibrosis among hepatitis B patients, with a focus on distinguishing between varying fibrosis levels.
**Material and Methods:** We conducted a retrospective analysis of hepatitis B patients who underwent liver biopsy and enhanced abdominal CT within a three-month interval from 2017 to 2023. Patients were divided into three fibrosis groups based on histopathology, and both ratios were measured during the portal phase of CT. Statistical analyses, including the Mann–Whitney U test and logistic regression, were used to assess the diagnostic performance, and receiver operating curve analysis determined optimal cutoff values.
**Results:** The L distance ratio and caudate right lobe ratio were significantly higher in advanced fibrosis (Group 3) compared to early fibrosis (Group 1), with cutoff values set at 0.79 for the L distance and 0.89 for the caudate right lobe ratio. The L distance ratio exhibited a stronger diagnostic performance, achieving an area under the curve of 0.81, compared to 0.65 for the caudate right lobe ratio.
**Conclusion:** The L distance ratio provides a reliable, non-invasive approach for staging fibrosis in hepatitis B patients, potentially serving as an effective alternative to invasive biopsy. Incorporating these CT-based ratios into routine imaging could streamline fibrosis assessment and reduce the need for biopsy in select patient groups. Further studies with larger cohorts are encouraged to validate these findings and enhance diagnostic accuracy.

### SS 10.6

### Novel imaging techniques for better characterization of chronic liver diseases

D. Catucci, S.U. von Däniken, V.C. Obmann, A. Berzigotti, L. Ebner, J.T. Heverhagen, A. Christe, P. Vermathen, A.T. Huber; *Bern/CH*

**Purpose:** This study analyzed the performance of intravoxel incoherent motion (IVIM) imaging parameters (tissue-diffusivity D, perfusion-fraction PF and pseudo-diffusion-coefficient D*) and liver stiffness (LS) measured by magnetic resonance elastography (MRE) to screen for chronic liver disease (CLD) and clinically significant portal hypertension (CSPH) on liver MRI.
**Material and Methods:** This prospective study included 103 patients without CLD (noCLD-group) and 82 patients with biopsy-proven CLD who underwent liver MRI including MRE and IVIM imaging between 03/2016 and 11/2023. Patients with CLD were subdivided based on their liver fibrosis degree: early CLD (F0-F1; eCLD-group, n = 21), intermediate CLD (F2; iCLD-group, n = 19), advanced CLD (F3-F4; aCLD-group, n = 20) and aCLD with CSPH according to the BAVENO VII consensus (aCLDPH-group; n = 22). IVIM imaging parameters (D, PF and D*) of the liver as well as LS were measured in all patients. Statistical analysis included the Kruskal-Wallis test for group comparison and receiver operating characteristic curve analysis with multiple logistic regression analysis for group differentiation.
**Results:** All parameters differed significantly between all groups ($p < 0.001$). The best parameter for detecting CLD and CSPH was LS with a cutoff value of >3.2 kPa (sensitivity 67%/specificity 96%), and >3.8 kPa (sensitivity 95%/ specificity 88%), respectively, both $p < 0.001$. For CLD-detection, a combination of D < 99 x $10^{-5}$ $mm^2$/sec and LS > 3.2 kPa increased the area under the curve from 0.89 with LS to 0.95. For CSPH-detection, a combination of D*< 434 x $10^{-5}$ $mm^2$/sec and LS > 3.8 kPa increased the specificity (90%) with a slightly lower sensitivity (91%, $p < 0.001$).
**Conclusion:** IVIM imaging parameters as well as LS measured by MRE allow noninvasive screening for CLD and CSPH.

### SS 10.7

### Assessment of portal hypertension severity and risk stratification in cirrhotic patients using radiomic features from routine CT scans

M.L. Watzenboeck[1], C. Sin[1], E. Iofinova[1], L. Balcar[1], G. Semmler[1], B. Scheiner[1], K. Lampichler[1], M. Mandorfer[1], L. Moga[2], P.-E. Rautou[2], M. Ronot[3], J. Menche[1], T. Reiberger[1], M. Scharitzer[1]; *[1]Vienna/AT, [2]Clichy/FR, [3]Paris/FR*

**Purpose:** The non-invasive evaluation of portal hypertension severity represents an unmet clinical need. Here, we developed a radiomics-based model using routine CT images to estimate hepatic venous pressure gradient (i.e., radio-HVPG), identify clinically significant portal hypertension (CSPH, i.e., HVPG ≥10mmHg) and predict a composite endpoint of liver-related events (LRE: decompensation and liver-related death) in cirrhosis patients.
**Material and Methods:** CT scans of 357 cirrhosis patients and 120 liver-healthy controls were utilized to develop a radiomics signature that was validated in both internal (n = 85) and external (n = 50) cohorts. The process involved volumetric liver and spleen segmentation, feature selection, and tuning hyperparameters through Bayesian optimization. The correlation between radio-HVPG and invasive HVPG was evaluated by Pearson correlation coefficient. Receiver operating characteristic analysis was used to evaluate the model's ability to diagnose CSPH. The predictive capabilities of radio-HVPG and invasive HVPG for liver-related events (LRE) were compared using Cox regression models.
**Results:** Radio-HVPG, calculated utilizing a random forest model based on 74 CT features, correlated with invasive HVPG (Pearson r = 0.63) and accurately identified CSPH (AUC = 0.89), which was confirmed for the external validation cohorts (Pearson r = 0.60; CSPH AUC = 0.78). Radio-HVPG performed similarly well in patients with compensated cirrhosis (Pearson r = 0.66; CSPH AUC = 0.87). Additionally, radio-HVPG predicted LRE when adjusting for MELD and albumin (adjusted hazard ratio, aHR: 1.14 [95% CI: 1.04–1.25], p = 0.005), performing comparably to invasive HVPG (aHR: 1.05 [95% CI: 1.01–1.1]; p = 0.021).
**Conclusion:** Radio-HVPG can be used to assess portal hypertension severity, identify CSPH, and predict LRE in patients with cirrhosis.

### SS 10.8

### Interest of Artificial Intelligence foundation models in radiology: use case for clinically significant portal hypertension in cirrhotic patients

H. Philippe[1], C. Dancette[1], P. Manceron[1], P. Hérent[1], J. Grégory[2], M. Ronot[2]; *[1]Paris/FR, [2]Clichy/FR*

**Purpose:** Foundation models (FM) represent a new generation of machine learning models that are claimed to offer improved performance. The aim of this study was to assess this new technology in a practical medical context: determining whether a patient has clinically significant portal hypertension (CSPH).
**Material and Methods:** This retrospective study included patients between January, 2010 and December, 2016 with histologically proven cirrhosis and who underwent a hepatic venous pressure gradient (HVPG) measurement and a contrast-enhanced CT within 60 days during liver transplantation or surgery workup. CSPH was defined as HVPG ≥ 10 mmHg. The FM was trained using the DINOv2 method on 20 million medical images. The performance for predicting CSPH was evaluated with area under the receiver operating characteristic curve (AUC). Performance was compared to that of BioMedCLIP (another image-based FM trained on 15 million medical images, and XGBoost (standard classification model with clinical data only (age, sex, GGT, AST, ALT, platelets count)).
**Results:** One hundred and eighty-four patients (142 male, mean (SD) 58 years (+/−10)) were included. Most patients had chronic hepatitis viral infection (hepatitis C (80/184 (43%)), B (29/184 (16%)). Mean (SD) Child-Pugh score was 6.4 (+/−1.8), and model for end-stage liver disease score was 11.2 (+/−4.7). CSPH was present in 99/184 patients (54%) with a mean (SD) HVPG of 12 (+/−7) mmHg. Our FM had an AUC of 0.752 for the diagnosis of CSPH and outperformed the BioMedCLIP FM (AUC 0.662) and XGBoost (AUC 0.701).
**Conclusion:** The FM performance for the diagnosis of CSPH was good and outperformed other classification models.

### SS 10.9

### "Liver extracellular volume fraction" a reliable marker in predicting high risk varices—prospective study in a tertiary care center

B. Kalimuthu, V. Kasi, A. Gunaseelan; *Coimbatore/IN*

**Purpose:** Variceal bleeding is a life-threatening complication of Cirrhosis. Endoscopy remains the gold-standard diagnostic method for confirming this; however, repeating it at frequent intervals is uncomfortable and challenging. The liver extracellular volume fraction (fECV) score obtained from Dual-Energy CT (DECT) could be a non-invasive investigation to predict liver fibrosis and high-risk varices (HRV). The purpose of our study is to determine the predictive accuracy of liver fECV measured using DECT, combined with serum albumin and platelets, in detecting HRV in patients with liver cirrhosis.
**Material and Methods:** This prospective observational study was conducted at KMCH, Coimbatore, over one year. It included 170 cirrhotic patients who underwent contrast-enhanced DECT of the abdomen and endoscopy as part of their clinical assessment. The fECV was quantified from iodine-only maps derived from the equilibrium phase DECT images.
**Results:** 81% of the patients with HRV show significantly higher fECV values (>33.96). This percentage further increased to 91% by including serum albumin and platelet count, with a statistical significance ($P < 0.001$).
**Conclusion:** Liver fECV scores, when combined with low serum albumin and platelet counts, strongly correlate with high-risk varices; therefore, it can be considered as an independent, reliable, and noninvasive predictor of high-risk varices. Thus, we can limit the endoscopy only for patients at greater risk of high-risk varices.

### SS 10.10

### Radiologic insights into sarcopenia: prognostic synergy in cirrhosis and chronic pancreatitis

N.B. Riswadkar[1], R. Ramachandran[2], U. Gupta[2]; *[1]Pune/IN, [2]Chennai, Tamil Nadu/IN*

**Purpose:** The role of sarcopenia, assessed by the psoas muscle index (PMI), in evaluating the prognosis of cirrhotic and chronic pancreatitis (CP) patients. To explore the correlation between PMI and traditional prognostic variables, like Child-Pugh, MELD-Na score, incidence of complications, clinical risk factors, including alcohol use, pancreatic enzyme replacement therapy (PERT), and diabetes, in CP patients. To establish diagnostic cut-off values for psoas muscle parameters to identify sarcopenia effectively.
**Material and Methods:** This is a single-centre ambispective observational study. PMI, measured at the L3 vertebral level on CT, provides an objective marker of sarcopenia. Study investigates the value of sarcopenia as a complementary tool to these established scores, focusing on its impact on complications, hospital stay, and disease severity.
**Results:** In a two-year observational study of 197 cirrhotic patients, PMI was measured using CT scans by isolating muscle tissue with a HU range of -30 to +110 and normalizing the area by height squared. Sarcopenia detected in 60%. Lower PMI values correlated with more severe cirrhosis—Child-Pugh category C and MELD-Na > 28. Patients with sarcopenia experienced higher rates of complications like ascites and longer hospital stays. PMI values consistently decreased with rising MELD-Na scores. In 261 CP patients, sarcopenia (46%) with higher prevalence in severe CP. The optimal corrected psoas muscle CSA cut-offs for sarcopenia were 2.79 $cm^2/m^2$ in males and 1.95 $cm^2/m^2$ in females. Significant associations include alcohol aetiology (69.3%), PERT usage (58.2%), and diabetes (62.8%).
**Conclusion:** Sarcopenia is a valuable adjunct to traditional prognostic tools in cirrhosis and CP advocating its inclusion in routine evaluation protocols for patients.

09:00 – 10:30 E 103/104

*Scientific Session SS 11*
### Interventional Radiology

### SS 11.1
### Transarterial bleomycin-lipiodol embolization (b/le) for giant hepatic hemangioma
E. Mikos, M. Zbroja-Putowska, K. Drelich, K. Pyra, A. Drelich-Zbroja*; Lublin/PL*

**Purpose:** Hepatic hemangiomas are among the most common benign liver tumors, with an incidence ranging from 4.0% to 20.0%. Large hepatic hemangiomas can cause symptoms, such as pain and bleeding. There is currently no gold standard in their treatment. One of the methods is transarterial bleomycin-lipiodol embolization (B/LE). The aim of this study is to evaluate the efficacy of treating hepatic hemangiomas with B/LE.
**Material and Methods:** We retrospectively reviewed 44 patients treated with B/LE between February 2021 and June 2022 in the Department of Interventional Radiology and Neuroradiology, Medical University of Lublin. Before the procedure, all patients were examined by magnetic resonance imaging (MRI) or computed tomography. Vascularized lesions were embolized by administration intraarterially of a mixture of bleomycin and lipiodol, closing the vascularization of the lesion, which was confirmed by control angiography. All patients were followed up clinically and by MRI for an average of 6 months.
**Results:** The size of the examined hemangiomas before the procedure ranged from 7 to 15 cm. After the first procedure, 28 out of 44 patients had an essential reduction in the size of the hemangioma, while 16 patients had the procedure repeated. Eventually, the size of the hemangiomas decreased significantly ($p \leq 0.05$). The average reduction in volume was 72.3%. The technical success described as the reduction of blood supply to the hemangioma was achieved in all the cases.
**Conclusion:** Transarterial B/LE is an effective procedure resulting in reduced size of hemangiomas and symptoms alleviation.

### SS 11.2
### Standalone medical therapy versus early endovascular intervention as the first step in the management of patients with primary Budd–Chiari syndrome—a randomized controlled trial
S. Biswas[1], S. Swaroop[1], S. Mehta[1], S. Gupta[2], S. Gamanagatti[1], S. .[1]*; [1]New Delhi/IN, [2]Delhi/IN*

**Purpose:** A randomized controlled trial comparing the clinical outcomes of performing an early endovascular intervention versus standalone medical therapy as the first step of management of Budd–Chiari syndrome (BCS).
**Material and Methods:** Symptomatic patients with primary BCS having either hepatic vein (HV) obstruction [Type II disease] or combined obstruction of HV and inferior vena cava (IVC) [Type III disease] were enrolled. Patients were randomized to receive SMT for 12 weeks or early endovascular intervention. Primary outcome measure was complete clinical response at 12 weeks defined as resolution of ascites, normalization of serum bilirubin, prevention of first episode of variceal bleeding or its recurrence and absence of spontaneous bacterial peritonitis.
**Results:** Of 44 patients randomized, 23 received SMT and 21 received early endovascular intervention. Baseline characteristics were comparable. In the early intervention group, 12 (52.2%) patients had Type II BCS (6 patients underwent transjugular intrahepatic portosystemic shunt (TIPS) and 6 underwent HV angioplasty and stenting), while 9 (47.8%) patients had Type III BCS (1 underwent IVC angioplasty alone, while 8 underwent TIPS with IVC angioplasty). A significantly higher proportion of patients undergoing early intervention (19 [90.5%, 95% CI: 69.6–98.8%]) achieved complete response at 12 weeks, compared to SMT (4 [17.4%, 95% CI: 4.9–38.7%]; $p < 0.01$). Early endovascular intervention was associated with significant improvement hepatic congestion (mean reduction in LSM of -16.9 [−24.9 to -8.8]) kPa as compared to the SMT group (-2.0 [−11.8 to 7.8]; $p = 0.02$) over 12 weeks.
**Conclusion:** Early endovascular intervention is associated with higher rates of symptomatic response and resolution of hepatic congestion compared to standalone medical therapy in primary BCS patients.

### SS 11.3
### Radiological abdominal complications following transjugular intrahepatic portosystemic shunt: a 5-year single-centre retrospective study
A. Igual Rouilleault, H. Font, A. Baiges, E. Belmonte, A. Soler Perromat, A. Darnell, J. García-Pagán, Á. García-Criado*; Barcelona/ES*

**Purpose:** This study aims to assess intra-abdominal complications following transjugular intrahepatic portosystemic shunt (TIPS), including those associated with the procedure and the prosthesis itself.
**Material and Methods:** All patients from our center who underwent TIPS between January 2019 and December 2023 were retrospectively recruited (n = 226). Clinical and demographic data were collected from medical records with a minimum follow-up of 10 months, and complications were recorded based on imaging studies (ultrasound, CT, and MRI). Descriptive statistics were applied to assess the incidence, timing of onset, and evolution of complications.
**Results:** Out of all participants, 19% developed intra-abdominal post-TIPS complications, either related to the procedure (vascular, biliary, or organ injuries, as well as locoregional inflammatory processes) or directly associated with the prosthesis (stenosis/occlusion, dysfunction, migration/misposition, and tipsitis/endotipsitis). A total of 61 complications were identified among those patients. The most common complications were secondary to shunt-creation (intrahepatic portal thrombosis and right suprahepatic branch thrombosis), accounting for 43% of all complications with a mean onset of 42 days post-placement (early complications). These were followed by prosthesis thrombosis, which represented 18% of complications, with a mean onset of 213 days (delayed complications). Moreover, locoregional inflammatory processes, such as acute pancreatitis, were identified as a newly reported early complication, accounting for 3% of cases, and appearing with a mean time of 5 days since TIPS placement.
**Conclusion:** The technical complexity of the TIPS procedure carries inherent risks. Radiologists must be aware of potential intra-abdominal complications following the intervention to ensure proper identification and improve the patient's prognosis.

### SS 11.4
### Quantitative evaluation of transjugular intrahepatic portosystemic shunt patency with dual-energy CT
M. Oberparleiter[1], S. Malakzadeh[2], H.-C. Breit[1], V. Obmann[3], D. Harder[1], D.T. Boll[1], C. Zech[1], M. Obmann[1]*; [1]Basel/CH, [2]Sion/CH, [3]Bern/CH*

**Purpose:** To evaluate the ability of dual-energy CT-derived quantitative iodine measurements to assess transjugular intrahepatic portosystemic shunt (TIPS) patency.
**Material and Methods:** All consecutive patients with an implanted TIPS (n = 57) who underwent dual-energy CT of the abdomen in the portal venous phase from 11/2014 to 07/2024 were included in this retrospective study. Only the most recent CT scan of each patient was included. Quantitative iodine concentrations were measured in the inferior vena cava, the portal vein, and the TIPS at the location of suspected occlusion, with 3 circular regions of interest each. Diagnostic accuracies of the absolute iodine concentration and the ratios of iodine concentrations of TIPS/portal vein and TIPS/inferior vena cava were evaluated with ROC analyses. Youden-Index was used to determine the optimal thresholds for TIPS occlusion.
**Results:** Fourteen patients showed TIPS occlusion, while 43 patients had a patent TIPS. The mean iodine concentration of the inferior vena cava, portal vein, open and occluded TIPS was 3.99, 4.65, 4.49, and 0.67 mg/ml, respectively. ROC-AUC for diagnosing TIPS occlusion was 0.998, 1.000, and 0.995, for absolute iodine concentration, iodine ratio TIPS/portal vein, and iodine ratio TIPS/inferior vena cava, respectively. Optimal thresholds were 1.40 mg/ml, and ratios of 0.262 and 0.263, respectively. Sensitivities and specificities for these thresholds were 95% and 100%, 100% and 100%, and 98% and 100%, respectively.
**Conclusion:** Dual-energy CT-derived iodine measurements allow for a high-accuracy quantitative assessment of TIPS patency and should be considered in difficult-to-diagnose cases.

### SS 11.5

### A holistic approach of systematic tumor and non-tumor biopsy during percutaneous radiofrequency ablation for hepatocellular carcinoma: diagnostic, prognostic and therapeutic impact

L. Blaise[1], M. Ziol[1], C. Campani[2], N. Ganne-Carrie[1], P. Nahon[1], G. N'Kontchou[1], J. Zucman-Rossi[3], L. Del Pozo[1], N. Barget[1], C. Boros[1], E. Desjonquieres[1], A. Demory[1], V. Grando[1], L.-C. Pescatori[1], O. Seror[1], J.-C. Nault[1], O. Sutter[1]; *[1]Bobigny/FR, [2]Florence/IT, [3]Paris/FR*

**Purpose:** To assess the feasibility, safety, and diagnostic/prognostic value of image-guided biopsies systematically collected during radiofrequency ablation (RFA) of Hepatocellular Carcinoma (HCC).

**Material and Methods:** We included patients with a first diagnosis of HCC who underwent tumor and non-tumor biopsies during percutaneous RFA between 2015 and 2021. We analyzed the safety, the rate of diagnostic tumor biopsies, and correlated histological results with prior diagnoses and oncological outcomes. Non-tumor liver biopsy results were compared with the clinical diagnosis of cirrhosis.

**Results:** 248 patients with 302 tumors treated by RFA were included. HCC was unifocal in 78% of cases, bifocal in 22%, with a median size of 24mm. Bleeding occurred in 6 cases (1.9%), requiring intervention in 2, without any related deaths. Biopsies were diagnostic in 66%, with positivity linked to nodule size ($P < 0.0001$), location ($P = 0.04$), and ultrasound visibility ($P = 0.004$). A discrepancy between non-invasive and histological diagnoses was observed in 5% of cases. Among 302 biopsies, 34% were non-diagnostic, 61% HCC, 3% cholangiocarcinoma/hepatocholangiocarcinoma, and 2% dysplastic nodules. Survival was significantly shorter in patients with cholangiocarcinoma/hepatocholangiocarcinoma ($P < 0.001$). Macrotrabecular-massive HCC was associated with higher recurrence rate ($P = 0.04$). The presence of >30% of tumor cells in formalin-fixed paraffin-embedded samples was associated with the expression of cancer genes at transcriptomic of the corresponding frozen samples, assuring their usefulness for molecular analysis. For non-tumor biopsies, cirrhosis was histologically confirmed in 82% of cases, with a 15% discrepancy between clinical and histological diagnoses.

**Conclusion:** Systematic tumor and non-tumor biopsies during RFA for a first diagnosis of HCC are feasible, safe, and provide diagnostic, therapeutic, and prognostic value.

### SS 11.6

### Efficacy, safety and long-term oncological outcomes of irreversible electroporation for hepatocellular carcinoma BCLC 0, A and B: a bi-institutional retrospective study

O. Sutter[1], M. Russo[1], J. Lebigot[2], A. Paisant[2], L. Besnier[2], F. Oberti[2], P. Nahon[1], N. Ganne-Carrie[1], L.-C. Pescatori[1], A. Petit[1], T. Molango[1], J.-C. Nault[1], C. Aubé[2], O. Seror[1]; *[1]Bobigny/FR, [2]Angers/FR*

**Purpose:** To assess the long-term outcomes of irreversible electroporation (IRE) for the treatment of unresectable Hepatocellular Carcinoma (HCC).

**Material and Methods:** Patients treated with IRE for HCC(s) ≤ 5cm, without vascular invasion or extrahepatic metastasis, across 2 French tertiary centres between 2012 and 2024 were included. Primary outcome was local tumor progression (LTP) following IRE alone. Adverse events, all-type recurrences, and survival rates were secondary outcomes.

**Results:** 246 patients with 324 HCCs (mean size: 24mm, range: 7–50mm) were included. Most patients were cirrhotic (86%, including 14% Child-Pugh B). Tumor stages before IRE were BCLC 0 ($n = 47$), A ($n = 171$) or B ($n = 28$), with 124 treatment-naïve patients. Reasons for choosing IRE included peribiliary central tumors (65%), contact with extrahepatic organs (12%) and/or impaired liver function (21%) or comorbidities (5%). Complete ablation was achieved in 96.6% of tumors after one ($n = 285$), two ($n = 15$), or three ($n = 4$) IRE procedures, or complementary thermal ablation ($n = 9$). The per-nodule LTP rate after complete ablation by IRE was 19.4%, 36.1% and 43.4% at 1, 3 and 5 years, respectively. Three-year overall-LTP rates, combining initial IRE failures and cases of local recurrences after successful treatment, were 27.5% for tumors ≤2cm, 48.4% for tumors 2–3 cm and 55.9% for tumors > 3cm. Median overall survival in treatment-naïve patients after first-line IRE was 36.8 months. Median progression-free survival in the whole population was 8.5 months. Sixteen patients (6.5%) experienced major adverse events, including three Grade-V fatal complications (1.2%).

**Conclusion:** IRE is an effective and safe treatment option for HCCs not amenable to other ablation techniques. Better local control is achieved for tumors ≤ 2 cm.

### SS 11.7

### MRI-based risk stratification for viable Hepatocellular Carcinomas after transarterial chemoembolization

W. Wang, Y.-C. Wang; *Nanjing/CN*

**Purpose:** To develop a risk stratification model of viable hepatocellular carcinomas (HCCs) following transarterial chemoembolization (TACE).

**Material and Methods:** This is a multi-center, retrospective study. HCC patients who underwent TACE as their initial treatment between February 2015 and October 2022 were included as the training set. A dataset from a multicenter clinical trial (NCT03113955) was served as the test set ($n = 102$). Another cohort ($n = 120$) was applied to correlate imaging with pathology. The outcome was tumor viability (viable vs nonviable) at six months after TACE. All participants in both the training and test cohorts underwent contrast-enhanced MRI at baseline, as well as at one and six months after TACE. Logistic regression analyses were conducted to identify clinical and imaging markers for high-risk viable tumors.

**Results:** A total of 203 viable tumors were included in the training set. The predictive model incorporated five imaging features: mild-moderate T2 hyperintensity, T2 peritumoral hyperintensity, diffusion restriction, irregular shape, and heterogeneity. The model achieved area under the curves of 0.85 (95% confidence interval [CI] 0.79, 0.90) for the training cohort and 0.88 (95% CI 0.81, 0.95) for the external test cohort. In the test cohort, there were significant differences in progression-free survival ($P = 0.004$) and 2-year overall survival ($P = 0.028$) between risk groups. When correlating with pathology, the high-risk group presented with higher incidence of microvascular invasion ($P = 0.003$) and liver capsule involvement ($P = 0.007$).

**Conclusion:** The risk model based on imaging features from post-TACE viable HCCs exhibits robust predictive power for tumor viability and long-term survival outcomes.

### SS 11.8

### Combined multiple regional anesthesia for microwave ablation of liver tumors: initial experience

L. Wang, M. Lu; *Chengdu/CN*

**Purpose:** To assess the feasibility and safety of combined multiple regional anesthesia (CMRA) as a potential strategy to decrease pain and reliance on intravenous analgesics during and after ultrasound-guided microwave ablation (US-guided-MWA) of liver tumors.

**Material and Methods:** A cohort of 75 patients with a total of 99 liver tumors who underwent US-guided-MWA of liver tumors were enrolled. These patients were randomly allocated into three groups: A, B, and C. Prior to the ablation procedure, Group A patients received a combination of hepatic hilar block (HHB), Transversus abdominis plane block (TAPB), and local anesthesia (LA). Patients in Group B were administered HHB in conjunction with LA, while those in Group C received TAPB and LA. Evaluative parameters included the Numerical Rating Scale (NRS) scores, consumption of morphine, incidence of complications, and factors influencing perioperative pain.

**Results:** All patients successfully underwent US-guided-MWA. The peak NRS scores for pain during ablation across the three groups were $2.36 \pm 1.19$, $3.28 \pm 1.59$, and $4.24 \pm 1.42$, respectively ($P < 0.01$), while the count of patients requiring morphine was 4/25, 8/25, and 13/25, respectively ($P < 0.01$). Postoperative NRS scores for the three groups at 4, 8, 12, 24, and 36-hour intervals demonstrated a pattern of initial increase followed by a decrease, with the order at each interval being Group A < Group C < Group B. Factors associated with increased pain included larger tumor size, greater number of tumors, and longer procedure and ablation time ($P < 0.05$). No major complications were recorded.

**Conclusion:** CMRA offers an effective and safe modality to manage pain during and after US-guided-MWA of liver tumors.

### SS 11.9

### Perihilar nerve block for percutaneous liver tumor ablation

J.L. Wei, C. Cho, C. Chu, B. Yuen, M. Law, H. Chan; *Hong Kong/HK*

**Purpose:** Perihilar nerve block (PNB) has been recently described as a method of pain relief during percutaneous liver tumor ablation. We aim to evaluate the efficacy and safety of PNB.
**Material and Methods:** In this retrospective cohort study, all patients who underwent image-guided percutaneous liver ablation with and without PNB from November 2022 to July 2024 in our institution were reviewed. Outcomes including technical success, complications, pain scores, intravenous analgesia and sedation requirements were compared between the nerve block and control groups.
**Results:** 30 patients were included in the nerve block group and 33 patients were included in the control group. The nerve block group experienced less pain during ablation compared to the control group, both overall (mean pain score 3.9 vs 5.5, respectively, $p = 0.018$) and within the non-subcapsular tumor subgroup (mean pain score 3.5 vs 5.3, respectively, $p = 0.018$). The nerve block group required lower dose of fentanyl compared to the control group (mean dose 17.5 µg vs 35.2 µg, $p = 0.001$).
**Conclusion:** PNB is a safe and effective technique for pain relief during percutaneous liver tumor ablation, which can lower the requirement for intravenous analgesics. PNB may be less effective in treating subcapsular liver tumors, but further studies will be needed to evaluate the relationship between PNB efficacy and tumor location.

**09:00 – 10:30** **E 105/106**

*Scientific Session SS 12*

### Advances in luminal imaging

### SS 12.1

### Preventing deaths and harm from misplaced nasogastric tubes: long-term results of radiographer-led pathway

S. Razak, G. Roe, H. Lambie, C. Donnellan, A. Cracknell, D. Tolan, J. Cairns; *Leeds/UK*

**Purpose:** To evaluate whether radiographer identification and action for misplaced nasogastric (NG) feeding tubes prevent patient harm and deaths in a multisite tertiary centre.
**Material and Methods:** Healthcare systems cause death and harm from feeding into misplaced nasogastric tubes. Service transformation was triggered by 4 adverse events in a 762-day period in 5 hospital sites covering 2,500 beds related to NG X-ray (NGXR) interpretation and miscommunication. Radiographers were trained to provide immediate NGXR interpretation and take action to remove or arrange repositioning for misplaced NG. Continuous surveillance of NG adverse events and radiographer NGXR interpretation accuracy were evaluated for 4,953 days after implementation.
**Results:** Trained radiographer NGXR evaluation and action on misplaced tubes prevented any episodes of harm after pathway implementation (0 events in 4953 days vs 4 events in 762 days). Pathway change reduced NGXR requesting (preintervention 75% vs 9.3% post) with increased first-line pH testing of gastric aspirate (preintervention pH 12% vs 84%). Accuracy of NGXR position compared with radiologist review was 99%.
**Conclusion:** Radiographer training and empowerment to immediately evaluate and act on NGXR findings produced sustained prevention of patient harm, reduced NGXR requesting, and improved pathway compliance. This should serve as a basis for a national patient safety programme. We propose an achievable NGXR accuracy standard over 95% in a large trained radiographer workforce.

### SS 12.2

### SBOM-AI trial: set-up and validation of artificial intelligence-based automatic total small bowel length measurement using CT and MRI in obese patients candidates to metabolic surgery

M. Zerunian[1], S. Nardacci[1], M. Valeri[2], N. Petrucciani[1], I. Toniolo[2], A. Berardo[2], L. Bonaldi[2], D. De Santis[1], D. Caruso[1], C. Fontanella[2], G. Silecchia[1], A. Laghi[1]; *[1]Rome/IT, [2]Padova/IT*

**Purpose:** Total small bowel length (TSBL) is crucial for successful metabolic/bariatric surgery. Non-invasive TSBL measurement will impact surgical strategy to avoid short-bowel syndrome after surgery. Cross-sectional imaging (CSI) might play an important role by measuring TSBL non-invasively. We aimed to set up a reliable artificial intelligence (AI)-based automatic method with preoperative CSI to measure the TSBL in candidates for bariatric/metabolic surgery.
**Material and Methods:** Multicentre prospective TRIAL included patients eligible for bariatric surgery (BMI > 35 kg/m$^2$ and one obesity-related comorbidity, BMI > 40 kg/m$^2$) who underwent the same-day MRI and CT-enterography before bariatric surgery. TSBL assessed right before the surgery at the operation table and TSBL < 250 cm considered the cut-off as risk for developing short-bowel syndrome. TSBL was obtained on MRI and CT by manual segmentation (Slicer3D). A CNN with U-NET was developed followed by expansive path and RELU and softmax activation to reduce the feature map. Adam optimization algorithm with a constant learning rate was used for the learning process. Training stopped after 100 epochs. DICE coefficient was calculated to quantify the accuracy of the prediction compared to annotated-by-radiologist images.
**Results:** Fifty patients enrolled (27 F, 26–54 years old, mean BMI 41.47). Patients underwent surgery showed a TSBL mean measured intraoperatively of 652.85 ± 87.37 cm. CSI showed good concordance with TSBL mean of 589.08 ± 82.95 cm ($k = 0.70$). DICE coefficient of the training set showed a DICE score ranging between 35% and 45%, confirmed in the validation set. All methods correctly categorized the patients according to the cut-off considered as risk factors for short-bowel syndrome.
**Conclusion:** Automatic AI-based segmentation of small bowel on non-invasive CSI might be a useful tool to assess obese patients to personalize the treatment and reduce complications.

Scientific Sessions

Authors' Index

### SS 12.3

### Quantification of small bowel motility using a rapid 3D imaging protocol

N. Sakai, R. Baker, J. Holmes, C. Dennis, N. Loefflad, E. Menozzi, H.E. Fitzke, G. Bhatnagar, A. Menys, J. Macnaughtan, A. Schapira, D. Atkinson, S. Mallett, J. Steeden, S.A. Taylor*; London/UK*

**Purpose:** MRI quantification of small bowel (SB) motility has potential as a novel diagnostic tool in Parkinson's disease. 2D bowel motility acquisitions do not encompass the whole bowel and are time consuming, often poorly tolerated by frailer patients. We developed a 3D motility sequence to address these limitations. We also tested segmenting anatomical volumes rather than individual SB loops to increase efficiency.

**Material and Methods:** Thirteen participants (7 female; median age 65) underwent MRI before and after 800 mL of 2% mannitol over 45 minutes. 3D motility images were acquired using an accelerated stack-of-spirals balanced steady-state free precession sequence (20 frames, temporal resolution 1s) and processed using a commercially available non-rigid registration algorithm (GIQuant, Motilent, UK). Two observers independently segmented SB loops in the right lower quadrant (RLQ) and the two anatomical volumes (RLQ and whole lower abdomen [WLA]). We compared pre- and post-drink motility scores. Bland–Altman plots were used to assess agreement between (i) motility from WLA and RLQ segmented regions compared to segmented RLQ SB and (ii) inter-observer agreement.

**Results:** Median (interquartile range) pre- and post-drink motility scores were 132.0 (61.4) and 182.6 (126.6) a.u., respectively.

Bland–Altman plots showed a mean bias (limits of agreement) of 14.3 for SB vs WLA (-38.8 and 67.4) and 16.3 for SB vs RLQ (-31.3 and 63.8) a.u. Mean bias for reader agreement was -0.62 (-27.0 and 25.7) a.u.

**Conclusion:** Rapid 3D quantification of SB motility using MRI is feasible. Segmenting anatomical volumes is repeatable and gives comparable motility data to manually segmenting SB loops, but is more time efficient.

### SS 12.4

### An abbreviated MRI protocol for quantification of small bowel motility in patients with Parkinson's disease

N.S. Sakai[1], C. Dennis[1], T. Parry[1], G. Bhatnagar[2], A. Menys[1], D. Atkinson[1], S.A. Taylor[1]*; [1]London/UK, [2]High Wycombe/UK*

**Purpose:** Altered gastrointestinal motility is an early feature of Parkinson's disease (PD), preceding diagnosis by >10 years. We developed an abbreviated magnetic resonance enterography (MRE) protocol, with reduced oral contrast volume and targeted motility sequences, for quantifying small bowel (SB) motility in PD patients and compared this to healthy volunteers (HVs).

**Material and Methods:** Participants underwent MRE before and after 800ml of 2% mannitol over 45 minutes. MR motility sequences (matrix 256 x 256, pixel spacing 1.5 x 1.5mm, 20 frames, temporal resolution 1s) were optimized to capture the maximum volume of SB in 4–6 slices. Images were processed using a commercially available non-rigid registration algorithm (GIQuant, Motilent, UK) to quantify SB motility. Median (range) motility (arbitrary units, a.u.) of the volume of SB in the right lower quadrant was calculated.

**Results:** 5 PD patients (median age 62 years, 3 males) and 5 HVs (median age 55 years, 3 males) completed the 800 ml oral contrast. Median scan durations in minutes (range) for pre-drink, post-drink, and combined scans were 18 (12–29), 14 (13–23), and 31 (27–46), respectively. Median baseline SB motility was 125 (73,176) a.u. in PD patients compared to 115 (76,273) a.u. in HVs; percentage change following oral contrast was +17% (range 13,31) in PD patients and +10% (range 3,22) in HVs.

**Conclusion:** The abbreviated MRE protocol for SB motility quantification proved feasible and detected motility changes after oral contrast. Baseline SB motility and post-contrast percentage change were higher in PD patients compared to HVs. Future studies will apply this protocol to assess motility variations at different stages of PD and investigate correlations with clinical symptoms.

### SS 12.6

### Cine MRI assessment of colonic motility in patients with active ulcerative colitis and associated symptoms of constipation

C. Dennis[1], W. Weston[1], I. Naim[1], B. Adebusoye[1], C. Miller[2], N. Zarate-Lopez[1], J. Pakpoor[1], H.E. Fitzke[1], A. Menys[1], J. Holmes[1], D. Atkinson[1], J. Markus[1], W. Blad[1], S. Mallett[1], S. Bloom[1], S.A. Taylor[1]*; [1]London/UK, [2]Buckinghamshire/UK*

**Purpose:** Some patients with ulcerative colitis (UC) paradoxically experience troublesome associated constipation (UCAC). We evaluated regional colonic motility in UC patients with and without constipation using two MRI quantification methods.

**Material and Methods:** Patients with active UC and UCAC (n = 11, mean age 39 years), active UC without constipation (n = 9, mean age 30 years), and healthy controls (n = 10, mean age 33 years) were prospectively recruited. All underwent 3T MR enterography 90 minutes after ingesting 2% mannitol solution including a 10-minute balanced steady-state gradient echo motility sequence positioned though the colon. Regions of interest were placed in colonic segments and two motility metrics (GIQuant and STMM, Motilent) derived. Median and interquartile range (IQR) were calculated and a Kruskal Wallis test applied to assess for differences between cohort measurement distributions.

**Results:** Median and IQR GIQuant scores in the left colon were 457 (393, 495), 368 (329, 418), and 385 (364, 424) for controls, UC without UCAC, and UCAC patients, respectively, with Kruskal Wallis suggesting a significant difference in measurement distribution between groups (p = 0.03). In the right colon, respective values were 427 (396, 471), 383 (355, 452), and 398 (380, 405). For individual colonic segments, only the sigmoid showed a difference in measurement distribution across the 3 groups: 440 (416, 521), 343 (306, 360), and 413 (349, 444) for controls, non-UCAC, and UCAC patients, respectively, p = 0.003. STMM measurements were similar between groups.

**Conclusion:** MRI motility metrics may be different across healthy controls, and UC patients with and without UCAC suggesting larger studies are justified, particularly if aberrant sigmoid motility acts as a "colonic break".

### SS 12.7

### Relationship between MRI-quantified Ileo-anal J-pouch motility, pouch inflammation and patient bowel frequency and symptom load

C. Dennis, W. Weston, W. Blad, I. Naim, A. Menys, S.A. Taylor*; London/UK*

**Purpose:** Patients with ileo-anal pouches often develop intrusive increased bowel frequency. GLP-1 agonists reduce frequency possibly inhibiting motility. Using motility MRI (mMRI), we investigated the relationship between pouch motility, endoscopic inflammation and symptoms.

**Material and Methods:** 30 ileo-anal pouch patients (mean age 44 years, 9 female) and 10 controls (mean age 44, 5 male: 5 with non-colonic Crohn's disease, 5 with ulcerative colitis) underwent MR enterography including a breath-hold balanced steady-state gradient echo motility sequence processed using GIQuant (Motilent). A radiologist placed regions of interest around the wall of the pouch and rectum (for controls), deriving a motility metric (expressed in arbitrary units (AU). Pouchoscopy was dichotomized into inflammation vs. no inflammation. Daily patient bowel frequency was dichotomized into =>10 and <10, and symptoms into "highly 'symptomatic' vs 'coping'. Motility was compared using Mann Whitney statistic.

**Results:** Five patients were excluded due to suboptimal pouch visualisation. Mean pouch motility was higher than rectal motility 157 AU (25 to 391) vs. 59 AU (23 to 104), p = 0.002. Patients with endoscopic inflammation (n = 11) had lower motility than those without (119 AU vs 185 AU, P = 0.05). Motility was higher in those with =>10 bowel movements (n = 13) compared to <10 (205 AU vs. 116 AU, P = 0.007. Pouch motility was correlated to bowel movement frequency (Rho = 0.46, p = 0.01), but not significantly different between highly symptomatic patients (n = 9) vs. those coping (183 AU vs. 132 AU) P = 0.1.

**Conclusion:** mMRI-quantified pouch motility is associated with bowel frequency and inflammation providing mechanistic insights for new therapeutic agents.

Scientific Sessions

Authors' Index

### SS 12.8
### MR defecography findings in patients with obstructive defecation syndrome
E. Ölçücüoglu, H. Tekdemir, M. Gökhan; *Ankara/TR*

**Purpose:** Obstructive defecation syndrome(ODS), which is a cause of primary constipation resulting from pelvic floor dysfunction, encompasses a large group, including rectocele, enterocoele, rectal vertical desensus and dyssynergic defecation. Here, we wanted to present the MR defecography findings of patients who were thought to have functional defakatuar disorders as a result of patient history, physical examination and in some cases manometric examination.
**Material and Methods:** A total of 471 patients who underwent MR defecography examination between February 2019 and June 2023 were retrospectively analyzed. All patients were examined with a 1.5 Tesla MR system (GE Medical Systems, Milwaukee, WI, USA) by using a body coil surrounding the pelvis. Patients were evaluated by a radiologist with 5 years of experience in pelvic floor imaging and 14 years of experience in radiology.
**Results:** Of the patients with MR defecography, 343 were female and 128 were male. The mean age of the patients was 49.2 years. Vertical desansus was present in 258 patients. The grade of vertical descansus was grade 1 in 73 patients, grade 2 in 145 patients, and grade 3 in 40 patients. 153 patients had rectocele. Grade 2 rectocele was present in 143 patients and grade 3 rectocele was present in 10 patients. Dyssynergic defecation was present in 303 defecographies.
**Conclusion:** Dynamic magnetic resonance defecography is an excellent non-invasive imaging method for the assessment of anatomical and functional pelvic floor dysfunction such as organ prolapse, ODS and fecal incontinence.

### SS 12.9
### Comparison of different MRI sequences for the visualization of abdominal hernia meshes
V. Englmaier, S. Langhart, A. Tognella, H. Alkadhi, D. Stocker, S. Stocker; *Zurich/CH*

**Purpose:** To compare the visibility of hernia meshes between different MRI sequences.
**Material and Methods:** This single-center retrospective institutional review board-approved study (May 2018–July 2023) included patients with hernia repairs and mesh placement who underwent standardized MRI (T2 HASTE, T1 in-phase [IP], T1 gradient-recalled echo [GRE], T2*). Two readers scored mesh visibility (0 = not visible, 1 = partly visible, 2 = fully visible) and subjectively assigned the best sequence for mesh visibility in each case. Repair location and mesh type were recorded.
**Results:** Ninety-five patients with 104 meshes were included (mean age 50.5 years, 48% female). 59% (61/104) of were repairs of the supra- and/or infraumbilical abdominal wall, 12% (12/104) of the umbilical region, 28% (29/104) of the inguinofemoral region and 2% (2/104) for parastomal hernias. “MRI visible” meshes were present in 65% (68/104). Mean visibility scores were 1.3 ± 0.6 for T2*, 1.0 ± 0.4 for T2, 1.0 ± 0.5 for IP and 1.0 ± 0.5 for T1 GRE $p < 0.001$). Overall, readers subjectively assessed T2* as the sequence with best visibility in 60% (R1) and 63% (R2) followed by T2 in 22% (R1) and 24% (R2).For “MRI visible” meshes, T2* was scored as best for mesh visibility in 87% (R1) and 90% (R2). For non-“MRI visible” meshes, T2 was scored as best for mesh visibility in 62% (R1) and 74% (R2).
**Conclusion:** Overall, T2* sequence showed significant superior visibility scores. Furthermore, there was a clear reader preference of T2* for the assessment of “MRI visible” and T2 for non-“MRI visible” meshes, respectively.

**10:00 – 10:45** **Emerald Room**

*Scientific Session SS 17*
### AI for prediction and more

### SS 17.1
### CT biological age (CTBA) using explainable artificial intelligence-driven abdominal CT biomarkers for “Age Deviation” and survival prediction in a large adult population
M.H. Lee[1], J. Garrett[1], M. Golden[1], D. Liu[1], R.M. Summers[2], P.J. Pickhardt[1]; *[1]Madison, WI/US, [2]Bethesda, MD/US*

**Purpose:** To evaluate CT biological age (CTBA) derived from explainable artificial intelligence-driven abdominal CT biomarkers for age deviation and survival prediction.
**Material and Methods:** In this retrospective cohort study, a CTBA model was constructed using abdominal CT biomarkers quantifying muscle, fat, aortic calcification, and bone mineral density (BMD) from a large adult cohort undergoing abdominal CT. Multivariable Cox proportional hazards modeling and index of prediction accuracy were used for weighted CT biomarker selection to determine individual 10-year survival probability and CTBA. “Age deviation” (=CTBA minus chronological age [CA]) was calculated and compared for 40–59-year-old (CA) individuals who died within 10 years versus those who survived 10 years or more from CT. A separate post-mortem CT (PMCT) cohort of recently deceased adults was used for external validation.
**Results:** 123,281 adults (mean age, 54 years [SD 17]; 47% women) with abdominal CT between January 2001 and January 2021 and PMCT in 4342 external decedents were included. Biomarkers of greatest importance to the CTBA model were muscle attenuation, aortic calcification, visceral fat attenuation, and BMD. Patients who died <10 years had positive age deviation (accelerated aging)—mean, 11 years (death < 1 year) and 6 years (death < 10 years) ($p < .001$). Patients who survived >10 years had negative age deviation (decelerated aging); mean, −11 years ($p < .001$). External decedents who died of acute causes had less accelerated aging than decedents who died of chronic disease—mean, 6 years vs. 16 years ($p < .001$).
**Conclusion:** CTBA is a composite predictive biomarker for survival using explainable CT biomarkers of aging.

### SS 17.2
### Explainable artificial intelligence-driven abdominal CT biomarkers are predictive of future cardiovascular events and improve cardiovascular risk prediction in adults without known cardiovascular disease
M. Lee[1], Y. Ha[1], B.E. Rush[1], J.W. Garrett[1], D. Liu[1], P. Nagpal[1], R.M. Summers[2], P.J. Pickhardt[1]; *[1]Madison, WI/US, [2]Bethesda, MD/US*

**Purpose:** To evaluate explainable artificial intelligence-driven abdominal CT cardiometabolic biomarkers for predicting future cardiovascular events (CVEs) in adult patients without known cardiovascular disease (CVD).
**Material and Methods:** In this retrospective cohort study, automated deep learning tools quantifying abdominal muscle (area and attenuation]), fat (visceral [VAT] area and attenuation), visceral-to-subcutaneous fat ratio (VSR), abdominal aortic calcification, and bone mineral density (BMD) were applied to abdominal CT scans in adults without CVD at the time of scanning. Subsequent cardiovascular events (myocardial infarction, stroke, heart failure) and clinical variables associated with CVD were determined from the electronic health record. Pooled Cohort Equations (PCEs) were calculated. Logistic regression, Kaplan–Meier survival curves, and Cox-proportional hazard models were performed for 10-year events.
**Results:** 53,855 adults (mean age, 56 years old; 44% male) without CVD had an abdominal CT between January 2000 and December 2020. 19,053 patients had a CVE on follow-up (median, 26 months). Muscle attenuation, VAT attenuation, VSR, aortic calcification, and BMD had the greatest survival curve separation. Higher CVE-free probability was observed with higher muscle attenuation and BMD. Lower CVE-free probability was observed with higher VAT area, VSR, and aortic calcification. The strongest predictors of 10-year CVE risk were muscle attenuation (AUC 0.67) and abdominal aortic calcification (AUC 0.66). Models combining CT biomarkers (AUC 0.73) outperformed PCEs (AUC 0.71). CT biomarkers improve PCEs performance and the model combining biomarkers and PCEs had the strongest performance (AUC 0.75).
**Conclusion:** Quantitative abdominal CT-based cardiometabolic biomarkers predict future CVEs and improve the predictive ability of existing clinical risk estimation tools for predicting future CVEs in adults without known CVD.

### SS 17.3

### Multitemporal MRI-based radiomics nomogram for the prediction of pathological complete response to total neoadjuvant therapy in patients with locally advanced rectal cancer

T. Hu, T. Tong, Y. Sun, J. Gong, C. Cai; *Shanghai/CN*

**Purpose:** We aimed to establish a multitemporal MRI-based radiomics model for the prediction of pathological complete response (pCR) for locally advanced rectal cancer (LARC) patients who received total neoadjuvant therapy.

**Material and Methods:** A total of 241 LARC patients were retrospectively enrolled and randomly divided into a training cohort (TC) and an internal validation cohort (IVC), 81 patients were prospectively enrolled as the prospective validation cohort (PVC). Patients underwent three times MRI at baseline status (t1), after standard neoadjuvant chemoradiotherapy (t2), and after consolidation chemotherapy (t3). Radiomics features were extracted from MRI images at the three time points, and delta features were calculated. We constructed four radiomics models. Model 1 was constructed based on the features of t1, t2, and delta1. Model 2 was constructed based on the features of t1, t2, and t3. Model 3 was constructed based on delta features. Model 4 was constructed based on all features. Finally, a combined model was built by combining significant clinical-MRI features and the radiomics score generated from the best radiomics model.

**Results:** Among the four models, model 4 achieved the best predictive efficacy with area under the curves (AUCs) of 0.836 (95% CI: 0.761–0.911), 0.769 (95% CI: 0.649–0.889) and 0.783 (95% CI: 0.653–0.912) in the TC, IVC and PVC. Finally, a combined nomogram for predicting pCR was constructed with significant clinical-MRI features and radiomics score 4, with AUCs of 0.841 (95% CI: 0.765–0.916), 0.765 (95% CI: 0.637–0.893) and 0.804 (95% CI: 0.685–0.923) in TC, IVC and PVC, respectively. The DCA curve confirmed that the combined model had clinical utility.

**Conclusion:** The combined nomogram achieved good predictive efficacy in predicting pCR for LARC patients.

### SS 17.4

### Radiomics studies for liver diseases: a systematic review of segmentation strategies

M. Mazzola[1], A. Blandino[2], F. Matteini[1], R. Cannella[1], G. Brancatelli[1], F. Vernuccio[1]; *[1]Palermo/IT, [2]Termini Imerese/IT*

**Purpose:** A reproducible segmentation is a prerequisite for obtaining reliable radiomics models. The ESR and ESORTC societies recommend multiple independent observer segmentations in studies with manual segmentation, multiple (>2) independent trained observers if automated segmentation is used as reference standard, and that items with ≥ 75% consensus on multiple segmentations are considered. The aim of this study was to investigate the segmentation strategies adopted in the available literature on radiomics applied to liver diseases.

**Material and Methods:** A systematic search was conducted on PubMed/Medline and Scopus databases to identify original studies assessing radiomics for liver diseases on US, CT and MRI, published until March 18, 2024. Of the 290 combined results, 126 were excluded (i.e. non-original, animal/phantom, PET/CT studies, non-liver related segmentation). Segmentation strategies of the 164 included studies were analyzed.

**Results:** Segmentation was either manual, semi-automatic, or automatic in 112 (68.3%), 12 (7.3%), and 7 (4.3%) studies, respectively, while multiple strategies were used in 25 (15.2%). Segmentation involved non-radiologists in 34 (20.7%) studies, and was undefined in 28 (17.1%) studies. Out of the 136 studies that included manual segmentation, in 25 (18.4%), segmentation was performed by one reader, and in 16 (11.8%), the number of readers was unclear. In 4/20 studies with automated/semiautomated segmentation, it was unclear if images were reviewed by humans. Inter-rater and intra-rater variability were calculated only in 88 (53.7%) and in 22 (13.4%) studies, respectively.

**Conclusion:** Segmentation strategies in radiomics studies on liver imaging are variable and oftentimes do not comply with current recommendations, with negative impact for clinical implementation of radiomics models.

### SS 17.5

### Attitudes towards artificial intelligence in healthcare and diagnostics among hospital patients in Greece

N.V. Pentara[1], D. Kavvadas[1], A. Papachristodoulou[1], V. Rafailidis[1], F. Busch[2], K. Bressem[2], P. Prassopoulos[1]; *[1]Thessaloniki/GR, [2]Munich/DE*

**Purpose:** Artificial intelligence (AI) is taking on a central role in the evolution of technology and consequently is increasingly being incorporated into various sectors, including healthcare. Its effective adoption in healthcare relies primarily on physicians and patients perception and attitudes. The Greek part of an international, multicenter, cross-sectional study on patient opinion towards AI in Healthcare is presented.

**Material and Methods:** A total of 187 patients were surveyed between February and November 2023 in the Radiology department. Inclusion criteria were patients aged 18 years or older who attended our department during the study period and voluntarily agreed to participate in the survey. A descriptive statistical analysis was performed, using chi-square tests for the correlation between categorical variables and t-tests to compare means.

**Results:** Men appear to have a more positive attitude towards AI than women ($p = 0.003$). However, men show a higher concern that AI will replace doctors compared to women ($p < 0.001$). Owning a smartphone appears to be associated with higher levels of trust in AI to provide reliable information ($p < 0.001$). Similarly, concern about increased healthcare cost due to AI varies by owning a smart device ($p = 0.003$), as most patients without tablets expressed concern. Furthermore, the use of AI seems to have a greater impact on people with a higher educational level.

**Conclusion:** This study provides a comprehensive perspective on patient attitudes towards AI. Findings indicate a need for customized AI implementation strategies that consider patients' health status and background, as well as the importance of explainable AI and physician oversight.

**11:00 – 12:30** **E 103/104**

*Scientific Session SS 13*
### Colorectal cancer: prediction, prognosis and response

### *SS 13.1*
### Body composition as a predictor of progressive disease in colon cancer
S. Nardacci, M. Polici, M. Zerunian, D. De Santis, B. Masci, A. Del Gaudio, F. Pucciarelli, D. Caruso, A. Laghi; *Rome/IT*

**Purpose:** To investigate body composition as a predictive biomarker of progression in patients with non-metastatic colon cancer.
**Material and Methods:** Patients with colon cancer (stage II-III) treated with up-front surgery, with availability of baseline CT, clinical, and histological data, were retrospectively enrolled. Patients with stage IV disease or those with CT unavailability were excluded. Body composition parameters were derived from baseline abdominal CT using artificial intelligence-based automatic segmentation software (DeepCatch). Up to 76 parameters regarding skeletal muscle mass, body fat distribution, bone density, liver density and fat-fraction were extracted from both whole segmentation volume and multi-slices region (last rib-iliac crest). Body composition features were compared between the two groups with ANOVA test. Predictive model and survival analysis were performed with ROC curves, Cox regression and the Kaplan–Meier method. $P < 0.05$ was considered significant.
**Results:** Three hundred patients (M:F = 171:129) were included in the study, 47/300 classified as progressive. Kaplan–Meier curves, built by stratifying the population according to the cut-offs provided by the ROC curves, showed that bone density at L1 ($p = 0.001$) and liver/spleen volume ratio ($p = 0.002$) were significant in the multi-slice analysis, while total bone volume ($p < 0.001$), total internal organs volume ($p = 0.001$), bone density at L1 ($p = 0.002$), and mean HU of liver and spleen (both $p < 0.001$) were significant in whole-volume analysis. Cox regression analysis revealed that liver/spleen volume ratio ($p = 0.001$, OR: 0.4), bone density at L1 ($p < 0.001$, OR: 1.4) and mean spleen HU ($p < 0.001$, OR: 0.6) were independent predictors of disease progression.
**Conclusion:** Body composition parameters, particularly bone density, may represent prognostic biomarkers of disease progression in patients with colon cancer.

### *SS 13.2*
### CT-based radiogenomic analysis to predict high-risk colon cancer (ATTRACT): a multicentric trial
M. Polici, M. Zerunian, D. Valanzuolo, D. Pugliese, D. De Santis, P. Mercantini, E. Pilozzi, A. Vecchione, M. Tarallo, E. Fiori, D. Caruso, A. Laghi; *Rome/IT*

**Purpose:** Clinical staging on CT knows several biases and radiogenomics approach could be proposed. The study aimed to test the performance of a radiogenomics approach in identifying high-risk colon cancer.
**Material and Methods:** ATTRACT is a multicentric trial, registered in ClinicalTrials.gov (NCT06108310). 300 non-metastatic colon cancer patients were retrospectively enrolled and divided into two groups, High-Risk and No-Risk, according to the pathological staging. Radiological evaluations were performed by two abdominal radiologists. For 118 patients, we achieved genomics. The baseline CT scans were used to evaluate the radiological assessment and to perform a 3D cancer segmentation. One expert radiologist used open-source software to perform the volumetric cancer segmentations on baseline CT scans in the portal phase (3DSlicer v4.10.2). Implementing the classical LASSO with a machine-learning library method was used to select the optimal features to build Model 1 (clinical-radiological plus radiomic feature, 300 patients) and Model 2 (Model 1 plus genomics, 118 patients). The performance of clinical-radiological interpretation was assessed regarding the area under the curve (AUC), sensitivity, specificity, and accuracy. The average performance of Model 1 and Model 2 was also calculated.
**Results:** In total, 262/300 were classified as High-Risk and 38/300 as No-Risk. Clinical-radiological interpretation by the two radiologists achieved an AUC of .58–.82 (95% CI: .52–.63 and .76–.85, $P < .001$, respectively), sensitivity: 67.9–93.8%, specificity: 47.4–68.4%, and accuracy: 65.3–90.7%, respectively. Model 1 yielded AUC: .74 (95% CI: .61–.88, $P < .005$), sensitivity: 86%, specificity: 48%, and accuracy: 81%. Model 2 reached AUC: .84, (95% CI: .68–.99, $P < .005$), sensitivity: 88%, specificity: 63%, and accuracy: 84%.
**Conclusion:** The radiogenomics model outperformed radiological interpretation in identifying high-risk colon cancer.

### *SS 13.3*
### Radiomics-based prediction of microsatellite instability in colorectal cancer: a non-invasive approach to treatment stratification
D. Mallardi, A. Galluzzo, G. Danti, G. Rossi, D. Lavacchi, V. Miele; *Florence/IT*

**Purpose:** Colorectal cancer (CRC) is a leading cause of death in the U.S., with treatment influenced by tumor staging and molecular features. One important prognostic and therapeutic indicator is microsatellite instability (MSI), which is caused by defective DNA mismatch repair (dMMR). According to NCCN guidelines, MSI-H/dMMR tumors show greater sensitivity to immunotherapy but a decreased response to 5-FU-based treatment. With growing use of neoadjuvant chemotherapy, there is interest in developing preoperative predictive models for MSI using non-invasive techniques. In order to promote personalized therapeutic approaches, this study focuses on developing a radiomics-based model that predicts MSI status using preoperative contrast-enhanced CT.
**Material and Methods:** Adult patients diagnosed with CRC who underwent staging CT scans prior to treatment, with previously determined MSI status, were retrospectively evaluated. The portal venous phase of contrast-enhanced CT was analyzed. Radiomic and statistical analysis was used to develop a model for predicting the MSI-H phenotype in CRC.
**Results:** Analysis was conducted on 46 adult CRC patients who had undergone staging CT scans with known MSI status. 20 statistically significant radiomic features from the portal-venous phase of CT images able to differentiate MSI from MSS patients were selected. LASSO regression was applied, selecting 4 features for model construction. The model's performance demonstrated an area under the ROC curve (AUC) of 0.844.
**Conclusion:** Results demonstrate the potential of the radiomics model as a non-invasive, cost-effective approach to MSI evaluation, supporting CRC therapy choices. By differentiating between patients who would benefit from immunotherapy or conventional chemotherapy, this approach could facilitate the transition from postoperative to preoperative treatment.

### *SS 13.4*
### Deep learning model based on multi-phase contrast-enhanced CT to predict pole pathogenic mutation in microsatellite stable colorectal cancer
Z. Guan, X. Zhang, Y. Sun; *Beijing/CN*

**Purpose:** Although POLE mutations are uncommon (2–4%), detecting POLE pathogenic mutations sensitive to immunotherapy is vital for microsatellite stable colorectal cancer (CRC). This study aimed to develop and validate a deep learning model for early and precise identification of POLE pathogenic mutations.
**Material and Methods:** We gathered CRC patients with confirmed POLE gene status from various centers. 25 patients with POLE pathogenic mutations and 94 patients with POLE wild-type were randomly assigned to training and testing groups in a 2:1 ratio. Regions of interest were manually outlined, covering the entire tumor and peritumoral adipose tissue. RGB images for four phases were fed into a VGG-GRU network, and the likelihood of each slice belonging to the pathogenic mutated group was generated. The patient's probability of belonging to the pathogenic mutated type was determined by averaging the probabilities of all slices from the patient. Model performance was evaluated using the area under the receiver operating characteristic curve (AUC), sensitivity, specificity, positive predictive value (PPV), and negative predictive value (NPV).
**Results:** In the testing group, for the slice-based prediction, the AUC, sensitivity, specificity, PPV, and NPV were 0.929, 86.8%, 84.3%, 61.9%, and 95.6%, respectively; for the patient-based prediction, the AUC, sensitivity, specificity, PPV, and NPV were 0.957, 75.0%, 90.6%, 66.7%, and 93.5%, respectively; for the combined model prediction, the AUC, sensitivity, specificity, PPV, and NPV were 0.969, 75.0%, 96.9%, 85.7%, and 93.9%, respectively.
**Conclusion:** The proposed deep learning model exhibited robust predictive ability for POLE pathogenic mutations and could aid in selecting potential immune-sensitive MSS/pMMR CRC patients.

### SS 13.5

### Impact of regional lymph node distribution patterns on microsatellite instability-high colon cancer

Z. Guan, Y. Sun; *Beijing/CN*

**Purpose:** Early identification lymph node metastasis is crucial for microsatellite instability-high (MSI-H) colon cancer, but with poor CT accuracy. To explore whether the distribution patterns of lymph nodes detected by CT are helpful in evaluating lymph node status.
**Material and Methods:** This two-center retrospective study included MSI-H colon cancer patients who underwent pretreatment CT and radical surgery (development set: December 2017 to December 2022; validation set: January 2016 to January 2024). Lymph node characteristics (clinical lymph node stage [cN], distribution patterns including vascular distribution, partial aggregation and partial fusion) associated with pathological lymph node metastasis (pN+) were selected and a novel clinical lymph node stage (ncN) was proposed in the development set. Diagnostic performance was verified in the validation set. Interobserver agreement was assessed by Fleiss Kappa. Clinical value of ncN was assessed by univariable logistic analysis in an independent set receiving neoadjuvant immunotherapy (August 2017 to February 2024).
**Results:** The study included 368 patients, 230 patients from development set, 86 patients from validation set, and 52 patients from treatment set. Only partial aggregation (odds ratio, 78.93; P < .001) and partial fusion (odds ratio, 21.51; P < .001) were significantly associated with pN+. ncN achieved superior diagnostic performance compared to cN in the validation set (sensitivity, 77% vs 83%, P = .5; specificity, 97% vs 26%, P < .001). Interobserver agreement for ncN and cN was moderate and poor (Kappa = 0.67, 0.48). ncN was associated with complete response after neoadjuvant immunotherapy (odds ratio, 0.05; P < .001).
**Conclusion:** ncN showed excellent diagnostic performance and could predict complete response after neoadjuvant immunotherapy in MSI-H colon cancer.

### SS 13.6

### CT-detected “Jellyfish Sign”: a new imaging biomarker for stage II/III microsatellite stability colon cancer prognosis

Z. Guan, Y. Sun, X. Zhang; *Beijing/CN*

**Purpose:** High heterogeneity in the prognosis of stage II/III microsatellite stability (MSS) colon cancer has increased interest in CT signs for predicting outcomes. This study aimed to analyze the association between pericolic fat density on CT and prognosis in stage II/III MSS colon cancer.
**Material and Methods:**
Stage II/III MSS colon cancer patients undergoing preoperative CT and radical surgery at Center A formed the discovery cohort. Patients from Center B were included for validation. The uneven increase in pericolic fat density, termed the “Jellyfish sign”, was defined as a linear wrapping structure around the intestinal wall due to uneven fat density increase. Survival analysis and time-dependent receiver operating characteristic (ROC) curves were used to assess the prognostic value of the “Jellyfish sign”.
**Results:** The discovery cohort had 236 patients, and the validation cohort included 50. The “Jellyfish sign” was observed in 89 (37.7%) and 13 (26.0%) patients, respectively. Perfect interobserver agreement was achieved ($\kappa$ = 0.803). The “Jellyfish sign” independently predicted both death (HR: 2.270, 95% CI: 1.188–4.338) and distant metastasis (HR: 4.751, 95% CI: 2.372–9.515) in the discovery cohort. The area under the ROC curve for predicting 3-year death and distant metastasis was 0.823 and 0.851 in the discovery cohort, and 0.942 and 0.754 in the validation cohort. For patients receiving adjuvant therapy, those with positive “Jellyfish sign” have a higher risk for death and metastasis (both P < 0.001).
**Conclusion:** The “Jellyfish sign”, an uneven increase in pericolic fat density on CT, is an adverse prognostic factor in stage II/III MSS colon cancer.

### SS 13.7

### Identifying prognostic biomarkers on baseline CT to support the use of neoadjuvant therapies in locally advanced colon cancer: a FOxTROT trial analysis

J.R. Platt[1], F. Elliott[1], P. Quirke[1], N. West[1], M. Seymour[1], L. Magill[2], K. Handley[2], D. Morton[2], J. Seligmann[1], D. Tolan[1]; *[1]Leeds/UK, [2]Birmingham/UK*

**Purpose:** Neoadjuvant therapies (NAT) are effective in locally advanced colon cancer (LACC) and require radiological patient stratification. However, the prognostic value of radiological features is unclear. We explored baseline CT data from the international FOxTROT trial for prognostic biomarkers to support the identification of high-risk patients for NAT.
**Material and Methods:** The following radiological variables reported by radiologists during the trial were included: tumour side (right/left), T stage (T3/4), depth of extramural tumour extension (mm), maximum single-wall tumour thickness (mm), N stage (N0/1/2), lymph nodes ≥ 10 mm (yes/no), and extramural venous invasion (EMVI; yes/no). Univariable and multivariable analyses for time to recurrence (time from randomisation to disease recurrence) were performed using Cox proportional hazards models. Analyses were repeated according to mismatch repair (MMR) status.
**Results:** 1,052 participants were included. On univariable analysis, recurrence risk was significantly higher with T4 staging (P = 0.001), greater tumour extension (P < 0.001), greater tumour thickness (P = 0.001), a lymph node ≥ 10 mm (P = 0.01) and EMVI (P = 0.02). Adjusted for T stage, greater tumour extension and thickness remained prognostic (P = 0.001 and P = 0.01, respectively). Tumour extension was associated with recurrence risk in proficient, but not deficient, MMR tumours (P < 0.001 vs. P = 0.39, respectively). Conversely, T4 and N2 staging were prognostic in deficient, but not proficient, MMR tumours (P = 0.03 and P = 0.04 vs. P = 0.10 and P = 0.42, respectively).
**Conclusion:** In LACC, several baseline CT features are predictive of recurrence but differ according to MMR status. Further biomarker discovery and validation are required to identify features with greatest utility in selecting high-risk patients for NAT in clinical practice.

### SS 13.8

### Characterising morphological changes on CT after neoadjuvant chemotherapy in locally advanced colon cancer: initial exploratory analysis within the FOxTROT trial

J.R. Platt, R. Frood, A. Scarsbrook, N. West, J. Seligmann, D. Tolan; *Leeds/UK*

**Purpose:** Neoadjuvant chemotherapy (NAC) represents a new treatment option for locally advanced colon cancer (LACC). The radiological response to NAC has not been well described and its relationship with recurrence risk remains unclear. This preliminary analysis characterises CT-based morphological changes in primary tumours following six weeks of NAC within the FOxTROT multicentre trial.
**Material and Methods:** Baseline and post-NAC CT scans from a sample of trial participants were evaluated for the following primary tumour features: depth of extension (mm), maximum single wall thickness (mm), length (mm), and volume ($cm^3$; manually segmented). These features were compared between baseline and post-NAC scans using Wilcoxon signed-rank tests. Percentage changes were calculated and compared between tumours with and without pathological regression using Mann–Whitney U tests.
**Results:** Paired scans from 30 participants were included. Pathological regression was present in 21 tumours and absent in 9. Median depth of extension, maximum thickness and volume were significantly lower following NAC (8 mm vs. 4 mm, 21 mm vs. 16 mm and 34.2 $cm^3$ vs. 18.6 $cm^3$, respectively; P < 0.01 for all), with median percentage reductions of 56.4%, 27.9% and 45.5%, respectively. Median tumour length did not change significantly following NAC (50 mm vs. 43.5 mm; P = 0.78). Percentage reductions were not significantly different between those with or without pathological regression (P > 0.1 for all).
**Conclusion:** NAC induces significant reductions in tumour extension, thickness, and volume in LACC, highlighting potential utility for assessing treatment response and disease activity. Further research is needed to clarify the prognostic implications of radiological response and applications in response-adapted treatment strategies.

### SS 13.9

### Clinical and imaging features may help differentiate desmoid tumors from peritoneal disease in patients with resected colon cancer

M. El Homsi[1], L.J. Fuqua[1], M. El Amine[1], H. Khasawneh[2], D. Hamdan[1], S. Lobaugh[1], M. Capanu[1], L.A. Rodriguez[1], J. Golia Pernicka[1]; [1]New York, NY/US, [2]Dallas, TX/US

**Purpose:** To assess for clinical and imaging features that can aid the radiologist in distinguishing desmoid tumors from peritoneal disease in patients with resected colon cancer.

**Material and Methods:** Retrospective study included 30 patients with resected colon cancer with subsequent pathology-proven desmoid tumor or peritoneal disease. Three radiologists blinded to the histopathological information reviewed CT images and recorded for each patient: tumor location, size, multiplicity, shape, contour, margin, density, homogeneous/heterogeneous, and the presence of any other sites of metastatic disease. Demographic information was recorded: age, sex, race, the presence of familial adenomatosis polyposis, CEA level within 4 weeks of imaging, and time to development of disease from surgery. Clinical and imaging findings were compared using Fisher's exact test using the values from the most experienced reader.

**Results:** 30 patients were included, 18 (60%) with desmoid tumors and 12 (40%) with peritoneal disease. Median age at the time of surgery was 51 [20–79] years with 18/30 (60%) being men. From the imaging characteristics, a few features were statistically significantly associated with tumor status in univariable analysis: lesion margin (lobulated/smooth versus spiculated) ($p = 0.024$), multiplicity of lesions ($p = 0.002$), and the presence of other metastatic disease ($p = 0.006$). Additionally, CEA (<5 ng/mL versus > 5 ng/mL) was statistically significantly associated with tumor status in UVA ($p < 0.001$).

**Conclusion:** Some imaging characteristics and CEA elevation can potentially help differentiate desmoid tumor from peritoneal disease in resected colon cancer patients aiding as a guide for clinical prognosis and next steps in treatment or surveillance.

### SS 13.10

### Artificial intelligence-based fully automatic body composition and survival analyses among non-metastatic colorectal cancer patients

B.K. Budai[1], T. Nonnenmacher[1], V. Damerell[1], V.M. Wagner[1], R. Hosch[2], F. Nensa[2], S. Hardikar[3], T. Lin[3], C. Kahlert[1], J.C. Figueiredo[4], C.I. Li[5], D. Shibata[6], D.A. Byrd[7], A.T. Toriola[8], O. von Stackelberg[1], C.M. Ulrich[3], H.-U. Kauczor[1], B. Gigic[1]; [1]Heidelberg/DE, [2]Essen/DE, [3]Salt Lake City, UT/US, [4]Los Angeles, CA/US, [5]Seattle, WA/US, [6]Memphis, TN/US, [7]Tampa, FL/US, [8]St. Louis, MO/US

**Purpose:** Our study aimed to assess the utility of artificial intelligence (AI)-based whole abdomen body composition analysis (BCA) in patients with colorectal cancer (CRC) and to investigate the associations of BCA with 2-year recurrence-free survival (RFS) and overall survival (OS).

**Material and Methods:** This study used preoperative abdominopelvic CT scans from 327 non-metastatic patients from the Heidelberg site of the international ColoCare Study. The "Body and Organ Analysis" tool was applied for BCA. The extracted volumetric measures (ml) including muscle, subcutaneous adipose tissue, visceral adipose tissue, and intermuscular adipose tissue (IMAT) were converted to estimated mass (kg) and indices (kg/$m^2$). The reproducibility between unenhanced and post-contrast CTs was assessed by intraclass correlation coefficients (ICC). Logistic, Cox hazards regression models, and log-rank tests were conducted for RFS and OS analyses. The models were tested by likelihood-ratio test and receiver operating characteristic curve analysis.

**Results:** All BCA measures yielded excellent reproducibility (ICC > 0.95). Logistic regression identified muscle and IMAT indices as independent predictors for OS, which also significantly increased the fitting and prediction performance of the clinical model with AUC = 0.86 (vs. 0.80), accuracy = 0.80 (0.65), and sensitivity = 0.80 (0.61). Cox regression for OS also identified the muscle index (HR: 0.22) and IMAT index (HR: 17.57) as independent predictors. Patients with IMAT index > 0.66 showed significantly worse OS compared to those with lower values ($p < 0.0001$). None of the BCA measures were independent predictors for RFS.

**Conclusion:** The muscle and IMAT indices, calculated from AI-based BCA of abdominopelvic CTs, exhibit a significant association with OS and provide additional prognostic value to clinical parameters among non-metastatic CRC patients.

**11:00 – 12:30** **E 105/106**

*Scientific Session SS 14*

### Oncology

### SS 14.1

### Tumor morphology on CT imaging is more influenced by the host tissue environment than by tumor type: implications for primary tumor prediction

S. Rostami, C. Guerendel, Z. Bodalal, R. Beets-Tan; Amsterdam/NL

**Purpose:** In tumor biology, tumor type and host tissue environment are recognized as key influencers of tumor morphology. However, this relationship has yet to be studied at the macroscopic level, especially in the context of artificial intelligence (AI)-driven medical imaging, predicting primary tumor types. This study explores the impact of these two biological factors on lesion-level tumor morphology, using advanced AI methods.

**Material and Methods:** Baseline contrast-enhanced CT scans of 1659 cancer patients (11,168 lesions of mainly metastatic colorectal and lung carcinomas) were manually segmented, and radiomic features encoding for the tumors' morphology were extracted. Unsupervised clustering approaches were used to determine morphological clusters, followed by qualitative and quantitative assessment (e.g., adjusted Rand index (ARI)) of agreement between the ground truth and identified clusters. External validation was performed using public datasets (>2000 CT scans).

**Results:** Optimized clustering on all lesions identified six morphological clusters. These showed poor association with tumor type. However, the host tissue environment was more strongly associated with the clusters, especially for metastatic lesions (ARI = 0.659). In particular, metastases in bone (91.756% in cluster 6), liver (48.272% and 38.942% in clusters 2 and 4, respectively), and lung (21.389% and 76.243% in clusters 1 and 5, respectively) were found to be situated more frequently in specific clusters, independent of their original primary tumor.

**Conclusion:** Tumor morphology seems more impacted by the local tissue environment than its original tumor type. Future AI models attempting to predict tumor type or identify the primary tumor, especially in cancers of unknown primary, should be aware that local (micro/macro)environmental factors might significantly confound predictions.

### SS 14.2

### The effect of adipose tissue radiodensity assessed on CT scan on survival outcomes in cancer patients: a systematic review

A. Bernardi[1], G. Besutti[1], K. Codeluppi[2], F. Venturelli[2], P. Giorgi Rossi[2], P. Torricelli[1], A. Pecchi[1], G. Ligabue[1], P. Pattacini[2]; [1]Modena/IT, [2]Reggio Emilia/IT

**Purpose:** We aim to determine the impact of adipose tissue radiodensity on survival outcomes in cancer patients.

**Material and Methods:** For this systematic review, a literature search was performed in Medline, Embase and Scopus up to January 2024. We included relevant existing systematic reviews, cross-sectional and cohort studies examining the role of adipose tissue radiodensity determined on baseline CT or PET/CT scan as a prognostic factor on overall and disease-free survivals (OS, DFS) in cancer patients. Title/abstract and full text screening, as well as data extraction, were performed by two reviewers. Risk of bias was performed by using ROBINS-E Tool. The narrative synthesis of results included effect on OS and DFS, overall and for different cancer sites.

**Results:** Of the 1254 studies screened based on title/abstract, 232 articles were assessed on full text, leading to the inclusion of 45 studies. Of these, 41 were retrospective and the most considered cancer site was colon-rectum (n = 10). Density assessment was heterogeneous in methods, mostly conducted on CT (n = 34), at a single slice level (n = 39, n = 21 at L3 vertebrae), and variably considering total, subcutaneous, visceral, and/or intermuscular adipose tissue. Increasing adipose tissue density was associated with worse OS and/or DFS in 34 studies. In two studies, increasing density had a protective effect, and in the remaining studies, no significant effect was found. Risk of bias was moderate-to-high for most studies.

**Conclusion:** Increasing adipose tissue radiodensity seems to be associated with worse prognosis in cancer patients. Considering the heterogeneity of methods and results and the moderate-to-high risk of bias, further evidence is needed.

### SS 14.3

### Is it possible to differentiate pancreatic and gastrointestinal sources of hepatic neuroendocrine tumor metastasis based on radiologic features?

F. Salahshour, H. Koohi, A. Teymouri, N. Sadighi, M. Safaie, F. Azmoudeh-Ardalan, A. Moradi, N. Ayoobi, A. Kasraian, M. Mehrabi Nejad*; Tehran/IR*

**Purpose:** To determine the primary source of neuroendocrine (NET) liver metastasis based on the imaging characteristics of metastatic lesions in gastrointestinal and pancreatic neuroendocrine tumors.

**Material and Methods:** Between 2014 and 2024, patients with hepatic NET metastasis with a definite gastrointestinal (GNET) or pancreatic (PNET) origin, who had multiphasic abdominal CT, were retrospectively included. We compared the size and number and imaging features of metastatic lesions including the enhancement patterns in each phase, the presence of cystic components, blood-fluid level, calcifications, lymphadenopathy, the tumor-to-liver and tumor-to-aorta attenuation ratio in all post-contrast phases between GNETs and PNETs hepatic metastasis.

**Results:** One hundred ninety-six metastatic lesions from 38 patients were included, of which 105 were pancreatic and 91 were gastrointestinal in origin. There was a significant correlation between the enhancement pattern and the primary tumor origin in three phases ($p < 0.001$). Most of the PNET metastases were arterial hyper-enhancing and got isodense on the portal and delayed phases, while GNETs were iso- or hyper-enhancing on the arterial phase, and hypodense on the portal or delayed phase images. Cystic components (34.29% vs. 9%, $p < 0.001$), blood-fluid levels (11% vs. 1%, $p = 0.001$), lymphadenopathy (62% vs. 23.5%, $p = 0.02$), and calcification (23.8% vs.0%, $p = 0.05$) were observed more frequently in GNET than in PNET metastasis. Apart from the tumor-to-liver ratio in arterial-phase CT ($p = 0.34$), all other calculated ratios were significantly different between the two groups ($p < 0.001$).

**Conclusion:** Multiphasic CT parameters can distinguish between pancreatic and gastrointestinal NET liver metastasis.

### SS 14.4

### Prognostic value of total liver tumor volume and sum of diameters during patient follow-up: an artificial intelligence-driven analysis of 604 patients with gastroenteropancreatic neuroendocrine tumors

K. Chupetlovska, M. Mladenov, P. Mugisha, G. Grassi, J. Engel, R. Beets-Tan, M. Tesselaar, M. Maas, S. Trebeschi*; Amsterdam/NL*

**Purpose:** RECIST 1.1 is widely applied for evaluating treatment response in gastroenteropancreatic neuroendocrine tumors (GEP-NETs), but it often results in inaccurate assessments due to their slow growth, heterogeneity in treatment responses, and inconsistencies in target lesion selection and measurement. Artificial intelligence (AI)-enabled segmentation has the potential to improve the accuracy, consistency, and efficiency of treatment response evaluations.

**Material and Methods:** GEP-NET patients were retrospectively included from an ongoing prospective single-center dataset. Using an AI segmentation model, a radiologist delineated all liver metastases across follow-up contrast-enhanced CT scans. Total liver tumor volume (TLTV) and the sum of diameters (SoD) of the two largest lesions (per RECIST 1.1) were extracted. A time-varying Cox model was employed to evaluate the prognostic value of TLTV changes over the patient follow-up, accounting for systemic treatments, interventions, age, sex, and cancer history. The correlation between TLTV and SoD was analyzed.

**Results:** A total of 604 patients (mean age: 61.4 years; 289 males) were included. Across 5,129 scans, patients averaged 5.8 follow-up scans over 3.8 years. The mean TLTV per scan was 262 cc, with approximately nine lesions per scan. Both TLTV and SoD were significant predictors of survival (HR = 1.21 and 1.07, respectively; $p < 0.01$). Although SoD and TLTV were strongly correlated ($r^2 > 0.90$), significant heteroscedasticity ($p < 0.001$) revealed that SoD is not a reliable estimator of TLTV, with the correlation dropping to $r^2 < 0.60$ after correction.

**Conclusion:** The strong correlation between tumor volume and survival supports the use of volumetric analysis for outcome prediction, suggesting that AI-assisted imaging may outperform diameter-based methods such as RECIST 1.1 in clinical practice, ultimately improving GEP-NET management.

### SS 14.5

### The added value of dual-energy CT in the detection of small bowel neuroendocrine tumors

A. Perrella, G. Bagnacci, N. Di Meglio, V. Di Martino, N. La Gioia, P. Bufano, F. Cammilletti, M. Mazzei*; Siena/IT*

**Purpose:** To evaluate whether dual-energy CT-enterography (DECT-E) improves the detection of primary small-bowel neuroendocrine tumors (SBNETs) compared with conventional CT-enterography (CTE).

**Material and Methods:** 38 patients (14 female) were selected between September 2020 and January 2022 from radiological and pathological databases: 19 had surgically confirmed SBNETs and 19 were in the control group. Two sets of images were loaded for each patient: CTE set, which included a polychromatic energy image at 120kVp, and DECT-E set, which included a virtual monoenergetic image at 40KeV and material density iodine maps. CT-images were anonymized, randomized and administered to nine radiologists with different experience levels. After a sufficient time interval to minimize recall bias, the radiologists independently reviewed the CTE and DECT-E images blinded to the histopathological results. The answers were collected through a survey-platform. Sensitivity, specificity, positive-predictive value (PPV), negative-predictive value (NPV), and accuracy were calculated for both set of images.

**Results:** For radiologists with less experience, sensitivity improved from 28% with conventional CTE to 88% with DECT-E, and accuracy increased from 58% to 85%. For radiologists with intermediate experience, sensitivity increased from 58% to 96%, and accuracy improved from 77% to 93%. Expert radiologists achieved 100% sensitivity, specificity, PPV, NPV, and accuracy with DECT-E, compared to 82% sensitivity and 91% accuracy with conventional CTE. DECT-E provided better diagnostic confidence ($p < 0.001$) with similar subjective image quality ($p = 0.274$).

**Conclusion:** DECT-E could significantly improve the accuracy of tumor detection in SB-NETs, particularly for less experienced radiologists. However, further investigations are needed.

### SS 14.6

### How do we predict microsatellite instability status in locally advanced gastric cancer? A preoperative CT-based proposal

M. Polici, M. Zerunian, G. Arrivi, F. Mazzuca, E. Pilozzi, P. Mercantini, E. Muttillo, L. Capece Minutolo del Sasso, D. De Santis, E. Iannicelli, D. Caruso, A. Laghi*; Rome/IT*

**Purpose:** Microsatellite instability (MSI) status represents a prognostic biomarker in locally advanced gastric cancer (LAGC). MSI testing relies on pathological examination. Thus, our study aimed to build a predictive model to predict MSI status based on preoperative CT scan findings.

**Material and Methods:** We enrolled a retrospective population of 80 patients with LAGC treated with surgery associated with adjuvant or perioperative chemotherapy from August 2017 to January 2024. The population was divided into microsatellite stable (MSS) and MSI groups. All patients had baseline CT scans acquired with enhanced, late arterial, and portal venous phases. Two expert-blinded abdominal radiologists performed both qualitative (cTN, cEMVI status, Dmax, cancer thickness, peritumoral fatty infiltration) and quantitative (CT-HU values and delta contrast enhancement) evaluations. All radiological features were compared between two groups, tested with ROC curve analysis. All features were used to build different predictive models (qualitative, quantitative, and combined models) with multivariate logistic regression.

**Results:** The final population had 23/80 and 57/40 patients classified as MSI and MSS, respectively. In the prediction of MSI status, the CT-HU values in arterial and portal venous phases resulted to be significant ($p < 0.01$) with AUC = 0.73 and 0.86, respectively. In the univariate analysis, the same features resulted in being independently correlated with MSS/MSI status with OR ≠ 1 ($P < 0.05$). The combined model (qualitative + quantitative) achieved an AUC = 0.89(P = 0.0003), the quantitative model achieved an AUC = 0.87(P = 0.01), and the qualitative model was not significant.

**Conclusion:** Evaluation of CT scan features could represent a noninvasive predictive imaging method for clinicians to classify LAGC in MSS or MSI and to select only patients suitable for chemotherapy.

### SS 14.7

### Developing new criteria for assessing treatment response in gastric cancer patients (modified gastric cancer RECIST—MgcRECIST): a groundbreaking and ambitious proposal

C. Sica[1], I. Monteleone[1], V. Di Martino[1], G. Bagnacci[1], A. Perrella[1], N. Di Meglio[1], R. Lenti[1], S.A. Piccioni[1], D. Marrelli[1], G. Mura[2], M. Mazzei[1]; *[1]Siena/IT, [2]Arezzo/IT*

**Purpose:** Many gastric cancer (GC) patients in Western countries are diagnosed with metastatic disease, with a median overall survival of less than twelve months. Multimodality and locoregional treatment for the management of metastatic disease has made “conversion surgery” feasible for patients previously considered inoperable. This has made imaging a central aspect of disease restaging, albeit with a known limitation in the use of RECIST 1.1.
**Material and Methods:** A prospective Italian registry (“METAGASTRO”) focusing on patients with metastatic GC and collecting both clinical and radiological data is currently underway. Our subanalysis included 187 patients from 6 centers affiliated to the Italian Research Group for Gastric Cancer who had received at least one cycle of chemotherapy and had both staging and restaging CT scans reviewed by experienced radiologists to obtain prognostic stratification according to RECIST 1.1 and mgcRECIST.
**Results:** Patients without peritoneal involvement or with less than two liver metastases or isolated pathological para-aortic lymph nodes (LN) showed significantly better survival (p = 0.001). Patients with at least one 15 mm LN whose short axis diameter (SAD) does not change at restaging have a worse prognosis. Variation in peritoneal cancer index (PCI) (within a 6-point interval) correlates with overall Survival (OS). The association of LN with SAD =/> 15 mm and 2 other different metastatic sites identifies the worst group of metastatic patients. RECIST 1.1 did not provide prognostic information (p = 0.233).
**Conclusion:** New criteria, including progressive disease if there was an increase in radiological PCI (>/= 6) and if almost one LN with SAD =/> 15 mm persisted/appeared at restaging, resulted in effective OS stratification (p < 0.001).

### SS 14.8

### Defining radiological peritoneal cancer index regions and automating region segmentation using artificial intelligence

L.J. Ewals[1], M.W. Tops-Welten[1], Y. Tong[1], C.H. Claessens[1], J.M. Piek[1], F. van der Sommen[1], M.J. Lahaye[2], I.H. De Hingh[1], M.D. Luyer[1], J. Nederend[1]; *[1]Eindhoven/NL, [2]Amsterdam/NL*

**Purpose:**
With emerging treatment options for peritoneal metastases (PM), accurate noninvasive methods are crucial to assess the presence and extensiveness of PM. This study aims to facilitate standardized and quantified assessment of PM on imaging by defining radiological Peritoneal Cancer Index (PCI) regions and automating region segmentation using Artificial Intelligence (AI).
**Material and Methods:** A Delphi study was conducted with international PM experts to establish consensus on radiological PCI region definitions. A 3D prototype of the PCI regions was developed by making practical adaptations to the surgical PCI regions. Experts evaluated the proposed structures and boundaries on a 5-point Likert scale via an online questionnaire. Consensus was defined as >75% agreement and <15% disagreement. As a proof of concept, regions 1–3 were segmented in 60 CT scans (50 for training, 10 for validation), and an AI model was trained and validated for automatic segmentation of these regions on CT images.
**Results:** In the first Delphi round, 45 experts participated, achieving consensus on 45 of 52 statements. A second round with 40 experts resulted in consensus for three additional structures and boundaries. While full consensus was not reached for the remaining definitions, a majority (>68%) agreed with the proposed definitions. The AI model segmented regions 1–3 with an average Dice score of 0.92, with minimal deviations from manual annotations.
**Conclusion:** The Delphi study defined radiological PCI regions. Initial AI results demonstrate accurate PCI region segmentation; therefore, the next step will be training of AI models for all regions.

### SS 14.9

### Staging peritoneal carcinomatosis in colorectal cancer: factors affecting diagnostic performance

J.R. Willemse, D.M. Lambregts, K. Chupetlovska, N.F. Kok, A.G. Aalbers, P. Snaebjornsson, R. Beets-Tan, M.J. Lahaye; *Amsterdam/NL*

**Purpose:** Whole-body MRI (WB-MRI) is increasingly used in the preoperative workup of colorectal cancer patients with peritoneal metastases who are scheduled to undergo cytoreductive surgery with hyperthermic intraperitoneal chemotherapy (CRS-HIPEC). This study evaluates the impact of MR image quality and patient intrinsic factors, such as body mass index (BMI) on the performance of WB-MRI in assessing the peritoneal cancer index (PCI).
**Material and Methods:** We retrospectively assessed the WB-MRIs of 41 patients for whom the MRI-based PCI (mrPCI) and final PCI after surgery (surgPCI) were already available from previous studies. Image quality, including the presence/type of artefacts, was scored on T2W, DWI and contrast-T1W using Likert scores. BMI and the presence/severity of ascites were also documented. These factors were correlated with the diagnostic performance of MRI, defined as the absolute difference between the mrPCI and surgPCI, using Pearson and Spearman correlation coefficients.
**Results:** Image quality was good-excellent in 36 (88%) cases. Artefacts with moderate to severe impact on diagnostic performance were found in only five (12%) patients. Diagnostic performance was significantly correlated with overall image quality (0.355, p = 0.023) and with the presence/severity of ascites (–0.355, p = 0.023). There was also a significant correlation between image quality and BMI (–0.699, p < 0.001), though BMI itself did not show a significant correlation with diagnostic performance.
**Conclusion:**
- Image quality affects diagnostic performance and is negatively affected by a higher BMI and the presence of ascites.
- Nevertheless, good-quality WB-MR images were achieved in the vast majority of colorectal cancer patients making WB-MRI a robust technique for peritoneal staging.

### SS 14.10

### Predicting locoregional treatment failure in anal cancer using multivariable clinical-imaging models

C. Ceuppens, L. Min, B. van Triest, W. Vogel, P. Snæbjörnsson, M. Maas, R. Beets-Tan, B.A. Grotenhuis, D. Lambregts; *Amsterdam/NL*

**Purpose:** To explore the value of a multivariable clinical-imaging model combining fluorodeoxyglucose positron emission tomography/computed tomography (FDG-PET/CT) with clinicopathological staging and risk factors to predict locoregional treatment failure in anal cancer patients prior to curative-intent chemoradiotherapy.
**Material and Methods:** Retrospective study of 99 patients with anal squamous cell carcinoma who underwent clinical staging including FDG-PET/CT followed by chemoradiotherapy and clinical follow-up of ≥1 year to assess locoregional treatment success/failure. Treatment failure was defined as residual pelvic disease or recurrence within one year following treatment. Several semi-quantitative parameters were derived from the primary tumour on FDG-PET/CT (MTV42%, SUVmax, SUVpeak, SUVskewness, SUVentropy, SUVuniformity, TLG). These were combined with age, sex, cTN-stage (defined on MRI and PET/CT), high-risk human papillomavirus (hr-HPV) status, and whether patients underwent sequential radiotherapy boost in a multivariable logistic regression analysis to predict locoregional treatment failure (using least absolute shrinkage and selection operator). Leave-one-out cross validation (LOOCV) was used to estimate the performance of resulting prediction models in an “unseen” dataset.
**Results:** Nineteen patients (19%) experienced locoregional failure. A clinical model incorporating male sex (Odds Ratio [OR] 9.41), cN+ stage (OR 1.99), and hr-HPV+ status (OR 0.17) showed the best predictive performance (AUC 0.81 after LOOCV). Adding PET/CT variables, including MTV42% (OR 1.22), did not improve performance (AUC 0.79). A PET/CT-only model performed poorly (AUC 0.36).
**Conclusion:** A clinical model combining male sex, node-positive stage, and hr-HPV-negative status resulted in the best performance to predict the risk for locoregional treatment failure prior to chemoradiotherapy. Semi-quantitative PET/CT parameters offered no additional prognostic value.

European Society
ESGAR
Gastrointestinal and Abdominal Radiology

### A

Aalbers A.G.: SS 14.9
Adebusoye B.: SS 4.1, SS 12.7
Agostini A.: SS 5.3, SS 5.5
Aguilera V.: SS 6.7
Ahmed N.: **SS 4.4**, SS 4.6
Alba P.: SS 10.2
Alberich A.: SS 6.6, SS 6.7
Aldhaheri R.: SS 9.5
Alemanno L.: SS 1.8
Alexiou E.: SS 1.4, SS 1.5
Alfaro-Cervello C.: SS 6.7
Ali M.: **SS 2.7**, SS 2.8
Alipanahi B.: SS 2.8
Alkadhi H.: SS 12.9
Almakhalas K.A.: **SS 8.3**
Almutawakel S.: **SS 5.9**
Alsarhani S.: SS 8.3
Alves N.: **SS 5.1**
Alvo J.: SS 16.1
Ammirabile A.: **SS 2.5**
An C.: SS 3.10
Anand E.: **SS 4.8, SS 4.9**
Angelucci C.: SS 2.9, SS 7.9
Antoniou A.: SS 4.8, SS 4.9
Araji A.: SS 7.8
Argiriadi P.: SS 7.8
Arias Graf M.: SS 16.1
Arrivi G.: SS 14.6
Ascari F.: SS 10.5
Asmundo L.: SS 9.7
Assis D.: SS 7.3
Atkinson D.: SS 12.3, SS 12.4, SS 12.7
Aubé C.: SS 11.6
Aydin E.: SS 6.5
Ayoubi Yazdi N.: SS 3.5, SS 14.3
Azmoudeh-Ardalan F.: SS 14.3

### B

Bae J. S.: **SS 1.3**
Bae S. H.: **SS 7.1**
Bagnacci G.: SS 9.9, SS 14.5, SS 14.7
Baheti A.: SS 8.6, SS 8.7
Baiges A.: SS 9.2, SS 11.3
Bailey A.: SS 7.6
Baillie S.: SS 4.7
Baker R.: SS 12.4
Balcar L.: SS 9.2, SS 10.1, SS 10.7
Bali M.: SS 9.8
Ballard D.: SS 4.8, SS 4.9
Ballester M.: SS 6.7
Bara A.: SS 1.4
Barber J.: SS 4.7
Barbier L.: SS 3.3
Bardhi E.: SS 15.2
Barget N.: SS 11.5
Barraza G.: SS 7.8
Bartolotta T.: SS 6.3, SS 10.2
Ba-Ssalamah A.: SS 10.1
Bastati-Huber N.: SS 10.1
Bates D.: SS 8.2
Bäuerle T.: SS 3.9
Beaufrère A.: SS 9.5
Beer L.: **SS 10.1**
Beets G.: SS 8.9, SS 8.10
Beets-Tan R.: SS 8.1, SS 8.9, SS 8.10, SS 14.1, SS 14.4, SS 14.9, SS 14.10
Belmonte E.: SS 11.3
Ben Abdallah I.: SS 16.4
Ben Arie G.: SS 9.4
Benedetti Cacciaguerra A.: SS 5.3, SS 5.5
Benke M.: SS 15.3
Benlloch S.: SS 6.7
Berardo A.: SS 12.2
Beretta C.: SS 1.8
Bergquist A.: SS 7.3, SS 7.4
Bernardi A.: **SS 14.2**
Berzigotti A.: SS 10.6
Besnier L.: SS 11.6
Besutti G.: SS 1.8, SS 10.5, SS 14.2
Bhagwanani A.: SS 4.4
Bhatnagar G.: SS 4.4, SS 4.6, SS 4.7, SS 4.10, SS 12.3, SS 12.4
Biswas S.: **SS 11.2**
Björk I.: SS 7.3
Blad W.: SS 12.6, SS 12.7
Blaise L.: SS 11.5
Blandino A.: **SS 6.3**, SS 10.2, **SS 17.4**
Bloom S.: SS 12.7
Bobowicz M.: SS 9.8
Bodalal Z.: SS 14.1
Bogveradze N.: SS 8.1
Boll D.T.: SS 11.4
Bonaldi L.: SS 12.2
Bond M.: SS 2.7, SS 2.8
Bonkur S.R.: SS 7.5
Boone D.: SS 4.4
Borbély R.: SS 16.2
Borde P.: SS 9.5
Borgheresi A.: SS 5.3, SS 5.5
Boros C.: SS 11.5
Bou Ayache J.: SS 7.8
Bouattour M.: SS 3.3
Boudjema K.: SS 3.3
Boyraz Hayat B.: SS 11.10
Bozzola C.: SS 1.8
Brancatelli G.: SS 3.1, SS 9.8, SS 17.4
Breit H.-C.: SS 11.4
Bressem K.: SS 17.5
Bricault I.: SS 9.5
Brismar T.: SS 2.10, SS 9.6
Broadhurst D.: SS 8.5
Bruno M.: SS 7.9
Budai B.: SS 5.2, **SS 13.10**, SS 15.3
Bufano P.: SS 14.5
Buffart T.: SS 2.7
Bungay H.: SS 7.6
Busch F.: SS 17.5
Buyuktoka R.E.: SS 6.5
Buzzetti R.: SS 9.1
Byrd D.A.: SS 13.10

### C

Cadi M.: **SS 2.4**
Cai C.: SS 17.3
Cairns J.: SS 12.1
Calame P.: SS 16.3
Camaione F.: SS 7.10
Cammilletti F.: SS 14.5
Campani C.: SS 11.5
Campbell M.: SS 7.7
Cannella R.: SS 3.1, SS 6.3, SS 10.2, SS 17.4
Capanu M.: SS 7.8, SS 8.4, SS 8.6, SS 13.9
Capece Minutolo del Sasso L.: SS 6.9, SS 14.6
Capitoni I.: **SS 9.9**
Cardobi N.: SS 15.2, SS 15.4
Carey E.J.: SS 7.5
Carletti G.: SS 7.9
Carlos Nahas S.: SS 8.7
Carter L.: SS 4.10
Caruso D.: SS 6.9, SS 9.1, SS 12.2, SS 13.1, SS 13.2, SS 14.6
Casale A.: SS 9.7
Cascella T.: SS 2.9, **SS 7.9**, SS 9.7
Castagnoli F.: SS 3.2
Castera L.: SS 6.4
Catellani B.: SS 7.10
Catucci D.: **SS 10.6**
Cauchy F.: SS 3.3
Cazelles A.: SS 2.4
Ceuppens C.: **SS 14.10**
Chahwan S.: SS 7.8
Chan G.: SS 8.3
Chan H.: SS 11.9
Chapelle T.: SS 2.7
Che F.: **SS 1.9, SS 3.4**
Chen H.: SS 1.2
Chhabra S.: SS 7.8
Chiesa C.: SS 7.9
Cho C.: SS 11.9
Chopinet S.: SS 3.3

Chowdhury K.: SS 4.4, SS 4.6
Christe A.: SS 10.6
Chu C.: SS 11.9
Chupetlovska K.: SS 8.10, **SS 14.4**, SS 14.9
Cigdem Karatayli E.: SS 4.2
Claessens C.H.: SS 14.8
Clarke C.: SS 4.7
Codeluppi K.: SS 14.2
Colecchia A.: SS 10.5
Comas M.: SS 4.3
Conte D.: SS 9.6
Corines M.J.: SS 8.6, SS 8.7
Correas J.-M.: SS 6.4
Cracknell A.: SS 12.1
Crespo A.: SS 6.7
Crnovrsanin N.: SS 2.8
Culver E.: SS 7.6
Cuoghi Costantini R.: SS 10.5
Curran S.: **SS 7.7**
Custers P.: SS 8.9
Cvejic S.: SS 7.9
Czerniak S.: SS 7.3

### D

Daccordi G.: SS 9.9
Dajti E.: SS 9.2
Damerell V.: SS 13.10
Dancette C.: SS 10.8
Danti G.: SS 13.3
Darnell A.: SS 9.2, SS 11.3
de Haas R.: SS 9.8
De Hingh I.H.: SS 14.8
De Hollanda A.: SS 6.2
de Jong K.: SS 2.7
De la Revilla Negro J.: SS 4.7
De Maria N.: SS 10.5
de Meijer V.: SS 9.8
De Robertis R.: SS 15.2, SS 15.4
De Santis D.: SS 6.9, SS 9.1, SS 9.10, SS 12.2, SS 13.1, SS 13.2, SS 14.6
de Wilt J.: SS 2.7
Deepak P.: SS 4.8, SS 4.9
Dehaene H.: SS 1.1
Del Gaudio A.: **SS 9.10**, SS 13.1
Del Pozo L.: SS 11.5
Delpla A.: SS 5.6
Demory A.: SS 11.5
Deng Y.: SS 7.6
Dennis C.: SS 12.3, SS 12.4, **SS 12.6, SS 12.7**
Desjonquieres E.: SS 11.5
Desterke C.: SS 9.5
Devi J.: SS 4.8, SS 4.9
Di Benedetto F.: SS 1.8, SS 7.10, SS 10.5
Di Martino V.: SS 14.5, SS 14.7
Di Meglio N.: SS 9.9, SS 14.5, SS 14.7
Di Sandro S.: SS 7.10
Ding N.: SS 4.4
Dioguardi Burgio M.: **SS 3.3**, SS 5.8, **SS 6.4**, SS 16.4
Do R.: SS 7.8
Donnellan C.: SS 12.1
D'Onofrio M.: SS 15.2, SS 15.4
Doukas M.: SS 9.8
Doyle S.: SS 1.1
Dracopoli N.: SS 2.8
Drelich K.: SS 11.1
Drelich-Zbroja A.: SS 11.1
Duan R.: SS 2.2
Dudás I.: SS 5.2, **SS 15.3**
Dwarkasing R.: SS 9.8

### E

Ebner L.: SS 10.6
El Amine M.: SS 13.9
El Homsi M.: SS 7.8, SS 8.2, **SS 8.6**, **SS 13.9**
el Khababi N.: SS 8.1
Elhence P.: SS 5.10
Elkrief L.: SS 9.2
Elliott F.: SS 13.7
Emir S.N.: **SS 10.4**
Emrich T.: SS 3.9
Engel J.: SS 14.4
Engelbrecht M.: SS 2.7
Englmaier V.: SS 6.8, SS 12.9
Erdmann J.: SS 9.8
Ernst R.: SS 8.6, SS 8.7
Erőss B.: SS 16.2
Escudero Garcia D.: SS 6.7
Evrimler S.: SS 4.2
Ewals L.J.: **SS 14.8**

### F

Faggioni L.: SS 9.8
Falcone T.: **SS 3.2**
Faluhelyi N.: **SS 16.2**
Fang Z.: SS 1.2
Fernandes M.D.: **SS 8.4**
Fernàndez-Clotet A.: SS 4.3
Fernandez-Paton M.: SS 6.7
Ferrandez A.: SS 6.7
Ferrusquía-Acosta J.: SS 9.2
Figueiredo J.C.: SS 13.10
Fijneman R.J.: SS 2.8
Fiori E.: SS 13.2
Fitzke H.E.: SS 4.7, SS 12.4, SS 12.7
Floros T.: SS 1.4, **SS 1.5**
Foerster F.: SS 3.9
Folseraas T.: SS 7.3
Font H.: SS 11.3
Fontanella C.: SS 12.2
Fowler K.: SS 9.8
Fox M.: SS 8.7
Francone M.: SS 2.5
Francoz C.: SS 3.3
Franklin J.M.: SS 4.4
Frittoli B.: SS 3.2
Frood R.: SS 13.8
Fuqua L.J.: SS 8.2, 8.4, SS 13.9

### G

Galluzzo A.: SS 13.3
Gamanagatti S.: SS 11.2
Gangai N.: SS 8.2, SS 8.7
Ganne-Carrie N.: SS 11.5, SS 11.6
García-Calonge M.: SS 9.2
García-Criado M.-Á.: SS 6.2, SS 11.3
García-Pagán J.: SS 9.2, SS 11.3
Garnier H.: SS 16.4
Garrett J.W.: SS 10.3, SS 17.1, SS 17.2
Garteiser P.: SS 6.4
Garzelli L.: SS 16.4
Gayat C.: SS 9.2
Geisel D.: SS 2.10, SS 9.6
Gerhards M.: SS 2.7
Geubels B.: SS 8.10
Ghaffar R.: **SS 5.7**
Ghodekar P.: SS 4.7
Giaccardi L.: SS 9.7
Giacomelli G.: **SS 5.3**, SS 5.5
Gibbs P.: SS 8.7
Gigic B.: SS 13.10
Gioia S.: SS 9.2
Giorgi Rossi P.: SS 14.2
Giovagnoni A.: SS 5.3, SS 5.5
Giovannetti E.: SS 2.8
Giudicelli H.: SS 9.2
Gjataj E.: SS 10.5
Gleeson D.: SS 7.7
Goedegebuure E.P.: **SS 8.1**
Gökhan M.: SS 12.8
Golden M.V.: **SS 2.1**, SS 17.1
Golia Pernicka J.S.: SS 8.2, SS 8.4, SS 13.9
Gollub M.J.: SS 8.2, SS 8.4, SS 8.6, SS 8.7
Gong J.: SS 17.3
González-Alayon C.: SS 9.2
Goodwin M.: SS 7.2
Goodwin T.: SS 7.2
Gourtsoyianni S.: SS 1.4, SS 1.5
Gouta C.: SS 1.4
Graafen D.: SS 3.9
Grando V.: SS 11.5
Grassi G.: SS 14.4

Grazioli L.: SS 3.2
Greer C.: SS 4.10
Grégory J.: SS 5.8, SS 10.8, SS 16.4
Grigoriadis A.: **SS 7.3**, SS 7.4
Grotenhuis B.A.: SS 8.9, SS 8.10, SS 14.10
Grunhagen D.: SS 2.7
Grunz J.-P.: SS 3.9
Guan Z.: **SS 2.3, SS 13.4, SS 13.5, SS 13.6**
Guerendel C.: SS 14.1
Gunaseelan A.: SS 10.9
Guner G.: SS 15.5
Gupta R.: SS 9.10
Gupta S.: SS 11.2
Gupta U.: SS 10.10

### H

Ha Y.: SS 17.2
Hahn F.: SS 3.9
Halligan S.: SS 4.4, SS 4.6
Hamadouche M.: SS 6.4
Hamdan D.: SS 13.9
Hameed M.: **SS 4.1**, SS 4.4, **SS 4.6**, SS 4.7
Hamma S.: SS 7.3, **SS 7.4**
Handley K.: SS 13.7
Harder D.: SS 11.4
Hardikar S.: SS 13.10
Harrow P.: SS 4.7
Hart A.: SS 4.8, SS 4.9
Hartmann F.: **SS 9.8**
Hegyi P.: SS 16.2
Heim L.: SS 3.9
Helbren E.: SS 4.4
Hérent P.: SS 10.8
Hermans J.J.: SS 2.7, SS 5.1
Hernández-Gea V.: SS 9.2
Hettiarachchie N.: SS 2.10, SS 9.6
Heverhagen J.T.: SS 10.6
Higginson A.: SS 4.1
Hobeika C.: SS 3.3, SS 6.4
Holmes J.: SS 4.6, SS 12.4, SS 12.7
Hong S.: SS 7.1
Hoppe A.: SS 6.8
Horsthuis K.: SS 7.3
Horvat N.: SS 8.2, SS 8.4, SS 8.6, SS 8.7
Hosch R.: SS 13.10
Hu T.: SS 8.8, SS 10.6, **SS 17.3**
Huber A.T.: SS 10.6
Huiskens J.: SS 2.7, SS 2.8
Huisman H.: SS 5.1
Huitink I.: SS 2.8
Hwang J.: SS 7.1
Hwang S.: SS 5.4
Hyland R.: SS 4.4

### I

Iannicelli E.: SS 14.6
Ibrahim A.: **SS 8.7**
Ieva F.: SS 2.5
Igual Rouilleault A.: **SS 11.3**
Illerstam F.: SS 9.8
Iofinova E.: SS 10.7
Iskandar M.: **SS 1.1**

### J

Jackson E.: SS 7.6
Jang H.: **SS 6.1**
Jang J.: SS 6.1
Jarnagin B.: SS 7.8
Jarre G.: SS 1.1
Jayasekara C.R.: SS 7.5
Jennings V.: SS 4.7
Jeon S.: SS 1.3, SS 15.1
Jia X.: **SS 3.8**
Jiang H.: SS 3.8, **SS 3.10**
Jimenez-Pastor A.: SS 6.6, SS 6.7
Jiménez-Serrano S.: SS 7.3
Jonas J.: SS 6.8
Joshi D.: SS 7.3
Joshi S.: SS 4.8, SS 4.9

### K

Kaasen Jørgensen K.: SS 7.3
Kahlert C.: SS 13.10
Kalimuthu B.: **SS 10.9**
Kalisz K.: SS 9.10
Kaposi P.: SS 5.2, SS 15.3
Karamustafao G.: SS 10.4
Karoui M.: SS 2.4
Kartalis N.: **SS 2.10**, SS 9.6
Kasi V.: SS 10.9
Kasraian A.: SS 3.5, SS 14.3
Kauczor H.-U.: SS 13.10
Kavvadas D.: SS 17.5
Kazemier G.: SS 2.7, SS 2.8
Kedem Mashraki R.: SS 9.4
Kemmerich G.: SS 7.3
Kemna R.: SS 2.7, **SS 2.8**
Kemp W.: SS 7.2
Kernanet L.: SS 9.2, SS 9.3
Keshvala C.: **SS 8.5**
Khasawneh H.: SS 13.9
Khera P.S.: SS 5.10
Khmelinskii A.: SS 8.1
Khodabakhshi D.: SS 4.7
Kilic Y.: SS 4.4
Kim D.H.: SS 2.1
Kim H.: SS 2.1, SS 15.1
Kim J.: SS 1.3, **SS 5.4**, SS 15.1
Kim T.: SS 7.8, **SS 8.2**
Klaase J.: SS 2.7
Klein S.: SS 9.8
Kloeckner R.: SS 3.9
Koc Ö.: SS 9.2
Koç U.: **SS 11.10**
Koh D.-M.: SS 3.2
Kok N.: SS 2.7, SS 2.8, 14.9
Kolesova O.: SS 6.10
Koohi H.: SS 14.3
Korngold E.: SS 8.6, SS 8.7
Kristic A.: SS 10.1
Kruse D.: SS 9.10
Krutik T.: SS 9.4
Kulali F.: SS 10.4, **SS 15.5**
Kutaiba N.: **SS 7.2**, SS 9.8
Kuten J.: SS 8.6
Kuzhiyanjal A.: SS 4.7

### L

La Gioia N.: SS 14.5
Labiad C.: SS 2.4
Laghi A.: SS 6.9, SS 9.1, SS 12.2, SS 13.1, SS 13.2, SS 14.6
Lahaye M.J.: SS 8.1, SS 14.8, SS 14.9
Lambert B.: SS 1.1
Lambie H.: SS 4.4, SS 4.7, SS 12.1
Lambregts D.: SS 8.1, SS 8.9, SS 8.10, SS 14.9 SS 14.10
Lampichler K.: **SS 9.2, SS 9.3**, SS 10.7
Langhart S.: SS 12.9
Lanza E.: SS 2.5
Laurent A.: SS 3.3, SS 9.5
Laurent-Bellue A.: SS 9.5
Lavacchi D.: SS 13.3
Law M.: SS 11.9
Law W.: **SS 7.8**
Lazzeretti F.: SS 9.9
Leal A.: SS 2.8
Lebigot J.: SS 11.6
Leclercq W.: SS 2.7
Lecot F.: SS 2.4
Lee E.: SS 7.1
Lee J.: SS 1.3
Lee M.: **SS 17.1, SS 17.2**
Lee S.: SS 8.6, SS 8.7
Lekadir K.: SS 9.8
Lenti R.: SS 14.7
Levin G.: SS 7.8
Lewin M.: **SS 9.5**
Li C.I.: SS 13.10
Licha A.: **SS 5.6**
Liem M.: SS 2.7
Ligabue G.: SS 14.2

Lim R.P.: SS 7.2
Limbada M.: SS 7.3
Limdi J.: SS 4.7
Lin T.: SS 13.10
Lindsay L.: SS 4.10
Litjens G.: SS 5.1
Liu D.: SS 17.1, SS 17.2
Liu X.: **SS 3.7**
Llop E.: SS 9.2
Lobaugh S.: SS 7.8, SS 8.6, SS 13.9
Loefflad N.: SS 12.4
Lofino L.: SS 9.10
Londoño M.: SS 7.3
Lovász B.: SS 15.3
Lu M.: SS 11.8
Lubner M.G.: **SS 10.3**
Lucidi V.: SS 2.10, **SS 9.6**
Lumbard K.: SS 2.8
Lung P.: SS 4.8, SS 4.9
Luo X.: SS 9.2
Luyer M.D.: SS 14.8

### M

Maas M.: SS 8.1, SS 8.9, SS 8.10, SS 14.4, SS 14.10
Macnaughtan J.: SS 12.4
Maddaloni E.: SS 9.1
Magill L.: SS 13.7
Magistri P.: SS 1.8, SS 10.5
Magni V.: SS 2.9, SS 7.9
Mähringer-Kunz A.: **SS 3.9**
Majeed A.: SS 7.2
Malakzadeh S.: SS 5.8, SS 11.4
Maldonado Schoijet I.: **SS 16.1**
Mallardi D.: **SS 13.3**
Mallett S.: SS 4.1, SS 12.4, SS 12.7
Manceau G.: SS 2.4
Manceron P.: SS 10.8
Mandorfer M.: SS 10.1, SS 10.7
Marchegiani M.: SS 5.3, **SS 5.5**
Mariani A.: SS 2.4
Marin D.: SS 9.10
Mariotti F.: SS 5.5
Markus J.: SS 12.7
Marquardt J.: SS 3.9
Marrelli D.: SS 14.7
Márta K.: SS 16.2
Marti-Aguado D.: SS 6.6, SS 6.7
Marti-Bonmati L.: **SS 6.6, SS 6.7**
Marticorena Garcia S.R.: SS 5.9
Mascarin B.: **SS 15.2**, SS 15.4
Masci B.: **SS 6.9**, **SS 9.1**, SS 13.1
Maspero M.: SS 2.9
Mattana G.: SS 6.3, **SS 10.2**
Matteini F.: SS 17.4
Matteucci G.: SS 2.5
Maurovich-Horvat P.: SS 5.2, SS 15.3
Mazaheri Y.: SS 8.4, SS 8.6
Mazzaferro V.: SS 2.9
Mazzei M.: SS 9.9, SS 14.5, SS 14.7
Mazzola M.: SS 17.4
Mazzuca F.: SS 14.6
Mehrabi Nejad M.: **SS 3.5**, SS 14.3
Mehta S.: SS 11.2
Meijer G.: SS 2.8
Meiqari L.: SS 2.8
Menche J.: SS 10.7
Meng J.: **SS 4.5, SS 12.5**
Meng X.: **SS 2.2**
Menozzi E.: SS 12.4
Menys A.: SS 4.4, **SS 4.7**, **SS 4.10**, SS 12.3, SS 12.4, SS 12.6, SS 12.7
Mercantini P.: SS 13.2, SS 14.6
Meysner O.: SS 8.5
Miclea R.: SS 9.8
Middelburg T.: SS 7.3
Miele V.: SS 13.3
Mikos E.: **SS 11.1**
Milazzo M.: **SS 3.1**
Miller C.: SS 12.7
Min L.: SS 14.10
Miranda J.: SS 8.7
Mittler J.: SS 3.9
Mladenov M.: SS 14.4
Mocchegiani F.: SS 5.5
Moga L.: SS 9.3, SS 10.7
Mohkam K.: SS 3.3
Mohsin M.: SS 4.4, SS 4.6
Molango T.: SS 11.6
Molenaar I.: SS 2.7
Molnár D.: SS 16.2
Mongort C.: SS 6.7
Monteleone I.: SS 14.7
Monton C.: SS 6.7
Moos S.: SS 2.7, SS 2.8
Moradi A.: SS 3.5, SS 14.3
Moran G.: SS 4.7, SS 4.10
Morawska I.: **SS 2.6**
Moreno C.C.: SS 8.6, SS 8.7
Moreno M.: SS 6.2
Morton D.: SS 13.7
Mosconi C.: SS 9.6
Mugisha P.: SS 14.4
Müller L.: SS 3.9
Munasinghe C.: SS 8.5
Munyal R.: SS 4.7
Mura G.: SS 14.7
Muttillo E.: SS 14.6

### N

Nagpal P.: SS 17.2
Nahon P.: SS 11.5, SS 11.6
Naim I.: SS 4.7, SS 4.10, SS 12.6, SS 12.7
Nardacci S.: SS 9.1, SS 12.2, **SS 13.1**
Nault J.-C.: SS 11.5, SS 11.6
Navarrete-Perez I.: SS 6.7
Nayagam J.: SS 7.3
Nederend J.: SS 14.8
Nensa F.: SS 13.10
Nickel D.: SS 7.1
Niemantsverdriet R.: SS 9.8
Ninika A.: SS 1.5
N'Kontchou G.: SS 11.5
Nocetti L.: SS 1.8
Nonnenmacher T.: SS 13.10
Norlin A.: SS 2.10, SS 9.6
Nota I.: SS 2.7, SS 2.8
Nougaret S.: SS 8.2, SS 8.6, SS 8.7
Nuzzo A.: SS 16.4

### O

Oberparleiter M.: **SS 11.4**
Oberti F.: SS 11.6
Obmann M.: SS 11.4
Obmann V.: SS 10.6, SS 11.4
Ocholi A.: SS 4.7
Ölçücüoglu E.: **SS 12.8**
Olivas I.: SS 7.3
Olodo-Atitebi S.: SS 7.6
Onyeador N.: SS 4.7
Ordás I.: SS 4.3
Ou X.: **SS 8.10**
Ouyang S.: SS 1.2
Özdemir M.: SS 11.10

### P

Paek M.: SS 7.1
Paisant A.: SS 9.5, SS 11.6
Pakpoor J.: SS 12.7
Pané A.: SS 6.2
Pansini M.: **SS 7.6**
Papachristodoulou A.: SS 17.5
Pappou E.: SS 8.2, SS 8.4
Paradis V.: SS 3.3
Park J.: SS 5.4
Paroder V.: SS 8.2
Parry T.: SS 12.3
Pasquazzo F.: SS 15.4
Patel A.: SS 4.4
Patel K.: SS 4.7
Patel N.: SS 4.7
Patijn G.: SS 2.7

Pattacini P.: SS 14.2
Paulino Di Muzio B.: SS 7.2
Pavlides M.: SS 7.6
Pecchi A.: **SS 1.8**, **SS 7.10**, **SS 10.5**, SS 14.2
Pelzer U.: SS 5.9
Pentara N.V.: **SS 17.5**
Pereira B.: SS 6.6
Perez Girbes A.: SS 6.7
Perez-Rojas J.: SS 6.7
Perrella A.: SS 9.9, SS **14.5**, SS 14.7
Pescatori L.-C.: SS 11.5, SS 11.6
Petit A.: SS 11.6
Petkovska I.: SS 8.2, SS 8.7
Petrucciani N.: SS 12.2
Petta S.: SS 6.3, SS 10.2
Philippe H.: **SS 10.8**
Piccioni S.A.: SS 14.7
Pickhardt P.J.: SS 2.1, SS 10.3, SS 17.1, SS 17.2
Piek J.M.: SS 14.8
Pietrasz D.: SS 3.3
Pilozzi E.: SS 13.2, SS 14.6
Pinto dos Santos D.: SS 3.9
Platt J.R.: **SS 13.7, SS 13.8**
Plumb A.: SS 4.4, SS 4.6
Polici M.: SS 6.9, SS 13.1, **SS 13.2**, **SS 14.6**
Polidori T.: SS 6.9
Ponsioen C.: SS 7.3
Porrello G.: SS 3.3
Pötter-Lang S.: SS 10.1
Pouvelle A.: SS 5.6
Prassopoulos P.: SS 17.5
Prince J.: SS 9.8
Procopet B.: SS 9.2
Prud'Homme C.: SS 9.5
Psoma F.: SS 1.5
Pucciarelli F.: SS 13.1
Pugliese D.: SS 13.2
Pugliese N.: SS 9.2
Puig J.: SS 4.3
Punt C.: SS 2.7, SS 2.8
Putrins D.: **SS 6.10**
Pyra K.: SS 11.1

### Q

Quirke P.: SS 13.7

### R

Radzina M.: SS 6.10
Rafailidis V.: SS 17.5
Rafart Martinez G.: SS 7.3
Ragaini E.: SS 2.5
Rahman S.: SS 4.4, SS 4.6
Ralli G.: SS 7.6
Ramachandran R.: SS 10.10
Rautou P.-E.: SS 6.4, SS 9.2, SS 9.3, SS 10.7
Ravaioli F.: SS 9.2
Razak S.: **SS 12.1**
Rea B.: SS 7.7
Reiberger T.: SS 9.2, SS 10.7
Reimerink J.: SS 7.3
Renzulli M.: SS 9.6
Ria F.: SS 9.10
Rijken A.: SS 2.7
Rimola J.: SS 4.3, SS 6.2
Rinaldi L.: SS 2.8
Riswadkar N.B.: **SS 10.10**
Roberts S.: SS 7.2
Roca P.: SS 1.1
Rodriguez L.A.: SS 8.4, SS 13.9
Roe G.: SS 12.1
Ronot M.: SS 3.3, **SS 5.8**, SS 6.4, SS 9.2, SS 9.3, SS 9.8, SS 10.7, SS 10.8, **SS 16.4**
Roor J.: SS 2.7
Rosen A.: SS 7.8
Rossi G.: SS 13.3
Rostami S.: **SS 14.1**
Ruers T.: SS 2.7
Rush B.E.: SS 17.2
Russo M.: SS 11.6
Ryu R.: SS 5.4

### S

Sabounchi A.: SS 5.8
Sack I.: SS 5.9
Sadighi N.: SS 14.3
Safaei M.: SS 3.5, SS 14.3
Saggiante L.: SS 7.9
Sahin S.: SS 6.5
Saidman J.M.: **SS 4.3**
Sakai N.S.: **SS 4.4, SS 12.3, 12.4**
Salahshour F.: SS 3.5, **SS 14.3**
Saltini D.: SS 9.2
Sanchez-Martin A.: SS 6.7
Sandrasegaran K.: **SS 7.5, SS 16.5**
Santangeli C.: SS 6.9
Sartoris R.: SS 5.8, SS 9.3
Sauvanet A.: SS 5.8
Scarsbrook A.: SS 13.8
Scatton O.: SS 3.3
Schapira A.: SS 12.4
Scharitzer M.: SS 10.7
Scharpf R.: SS 2.8
Scheiner B.: SS 9.2, SS 10.7
Schepis F.: SS 9.2
Schulz Ibaceta H.: SS 16.1
Schuurmans M.: SS 5.1
Schwartz L.H.: SS 8.7
Seager M.: SS 7.3
Seifouri S.: SS 3.5
Seligmann J.: SS 13.7, SS 13.8
Selvaraj E.: SS 7.6
Semmler G.: SS 10.7
Seror O.: SS 11.5, SS 11.6
Sethi-Arora K.: SS 4.7
Seymour M.: SS 13.7
Sgantzou I.: **SS 1.4**, SS 1.5
Shah A.S.: SS 7.5
Shalimar S.: SS 11.2
Sheedy S.P.: SS 8.6, SS 8.7
Shepherd T.: SS 4.7
Shibata D.: SS 13.10
Sica C.: **SS 14.7**
Siddiuqui A.: SS 4.7
Sidhu H.: SS 4.4
Sigel C.: SS 7.8
Silecchia G.: SS 12.2
Silva A.: SS 7.5
Silva A.N.: SS 7.5
Simon G.: SS 9.5, **SS 16.3**
Sin C.: SS 7.3, SS 10.7
Singh C.: SS 7.3
Sirlin C.: SS 6.7
Skidmore Z.: SS 2.8
Snaebjornsson P.: SS 14.9, SS 14.10
Soler-Perromat A.: **SS 6.2**, 11.3
Somogyiné Nagy Z.: SS 5.2, SS 15.3
Song B.: SS 1.6, SS 1.7, SS 1.9, SS 3.4, SS 3.6, SS 3.8, SS 3.10
Song Y.: SS 7.8
Soni S.C.: SS 5.10
Soro A.: SS 9.7
Sosa R.: SS 8.7
Sposito C.: SS 2.9
Spoto F.: SS 15.2, **SS 15.4**
Starmans M.: SS 9.8
Steeden J.: SS 12.4
Stocker D.: **SS 6.8**, SS 12.9
Stocker S.: SS 6.8, **SS 12.9**
Stocker-Ghafoor S.: SS 6.8
Stoehr F.: SS 3.9
Stoker J.: SS 2.7, SS 2.8, SS 4.8, SS 4.9
Summers R.M.: SS 10.3, SS 17.1, SS 17.2
Sun Y.: SS 2.3, SS 13.4, SS 13.5, SS 13.6, SS 17.3
Sureka B.: **SS 5.10**
Sutter O.: **SS 11.5, SS 11.6**
Swaroop S.: SS 11.2
Swijnenburg R.-J.: SS 2.7, SS 2.8
Szalai E.: SS 16.2
Szentesi A.: SS 16.2
Szijártó A.: SS 5.2, SS 15.3
Szücs Á.: SS 5.2, SS 15.3

### T

Taher M.: SS 3.5
Tamir S.: **SS 9.4**
Tan K.: SS 8.3
Tan N.: SS 7.2
Taouli B.: SS 9.8
Taprania P.: SS 5.10
Tarallo M.: SS 13.2
Taylor S.A.: SS 4.1, SS 4.4, SS 4.6, SS 4.7, SS 12.3, SS 12.4, SS 12.6, SS 12.7
Tekdemir H.: SS 12.8
Ten-Esteve A.: SS 6.7
Tesselaar M.: SS 14.4
Teutsch B.: SS 16.2
Teymouri A.: SS 14.3
Thavarajah S.: SS 16.4
Thomeer M.: SS 9.8
Thompson C.P.: SS 7.5
Thomson E.: SS 4.4, SS 4.6
Tiernan J.: SS 8.5
Tilman N.: **SS 8.9**
Tognella A.: SS 12.9
Tolan D.: SS 4.4, SS 4.7, SS 8.5, SS 12.1, SS 13.7, SS 13.8
Tong T.: **SS 8.8**, SS 17.3
Tong Y.: SS 14.8
Toniolo I.: SS 12.2
Tops-Welten M.W.: SS 14.8
Toriola A.T.: SS 13.10
Torricelli P.: SS 1.8, SS 7.10, SS 10.5, SS 14.2
Torzilli G.: SS 2.5
Tóth E.: SS 5.2
Touloupas C.: SS 5.6
Tozer P.: SS 4.8, SS 4.9
Tran D.: SS 10.3
Trebeschi S.: SS 14.4
Tremamunno G.: SS 6.9
Triay-Bagur A.: SS 7.6
Tulone A.: SS 6.3, SS 10.2
Turco L.: SS 9.2

### U

Ulrich C.M.: SS 13.10
Upponi S.: SS 4.7

### V

Vaiani M.: **SS 2.9**, SS 7.9, **SS 9.7**
Valanzuolo D.: SS 13.2
Valeri M.: SS 12.2
Valla D.: SS 6.4
Valsan A.: SS 9.2
van Amerongen M.: SS 2.7
Van Beers B.: SS 6.4
van Dam R.: SS 2.7
van Delden O.: SS 9.8
van den Bergh J.: SS 2.7, SS 2.8
van den Broek D.: SS 2.8
van der Sommen F.: SS 14.8
van Dieren S.: SS 2.7
van Eck S.: SS 2.7
van Griethuysen J.: SS 8.1
van Gulik T.: SS 2.7
van Lienden K.: SS 2.7
van Steijn D.: SS 2.8
van ,t Erve I.: SS 2.8
van Triest B.: SS 14.10
Van Waesberghe J.: SS 2.7
Vanaga I.: SS 6.10
Varshney V.: SS 5.10
Vass V.: SS 16.2
Vecchione A.: SS 13.2
Veenstra M.: SS 9.8
Velculescu V.: SS 2.8
Vendittelli P.: SS 5.1
Venturelli F.: SS 14.2
Verhoef C.: SS 2.7
Vermathen P.: SS 10.6
Vernuccio F.: SS 3.1, SS 17.4
Verpalen I.: SS 2.7, SS 2.8
Vesterhus M.: SS 7.3
Victor P.: SS 6.7
Viganò L.: SS 2.5
Vigh A.I.: **SS 5.2**, SS 15.3
Viksna L.: SS 6.10
Vilain S.: SS 16.4
Vilgrain V.: SS 1.1, SS 3.3, SS 6.4, SS 9.5, SS 9.8
Villanacci A.: SS 3.2
Villard C.: SS 7.4
Vincze Á.: SS 16.2
Vishnoi J.R.: SS 5.10
Vivarelli M.: SS 5.3
Vogel W.: SS 14.10
von Däniken S.U.: SS 10.6
von Stackelberg O.: SS 13.10
Vuyyuru S.: SS 4.7

### W

Wagner V.M.: SS 13.10
Wang J.: SS 1.2
Wang L.: **SS 11.8**
Wang M.: SS 4.5, SS 12.5
Wang W.: SS 11.7
Wang Y.: SS 3.8
Wang Y.-C.: **SS 11.7**
Watzenboeck C.: SS 9.3
Watzenboeck M.L.: **SS 10.7**
Wei J.L.: **SS 11.9**
Wei Y.: SS 3.4
Weinmann A.: SS 3.9
Wesdorp N.J.: SS 2.7, SS 2.8
West N.: SS 8.5, SS 13.7, SS 13.8
Weston W.: SS 4.7, SS 12.6, SS 12.7
Widmar M.: SS 8.2
Willemse J.R.: **SS 14.9**
Win A.: SS 4.7
Winata S.-H.: SS 7.2
Wu P.: SS 3.10
Wu Y.: **SS 1.7**, **SS 3.6**

### X

Xiao Y.: SS 1.2

### Y

Yadav T.: SS 5.10
Yang T.: **SS 1.6**
Ye Z.: SS 1.7, SS 3.6
Yee Liang T.: SS 8.3
Yeo J.: SS 4.7
Yetim B.: **SS 6.5**
Yoo J.: SS 1.3
Youn S.: **SS 15.1**
Yuen B.: SS 11.9

### Z

Zannotti A.: SS 5.3, SS 5.5
Zarate-Lopez N.: SS 12.7
Zbroja-Putowska M.: SS 11.1
Zech C.: SS 11.4
Zeeuw M.: SS 2.7, SS 2.8
Zeng Y.: SS 1.2
Zengin E.: **SS 4.2**
Zerunian M.: SS 6.9, SS 9.1, **SS 12.2**, SS 13.1, SS 13.2, SS 14.6
Zhang D.: SS 1.2
Zhang X.: SS 13.4, SS 13.6
Zhao B.: SS 8.7
Zheng J.: SS 8.4, SS 8.7
Zheng L.: SS 2.2
Zhou J.: **SS 1.2**
Zins M.: SS 5.6
Ziol M.: SS 11.5
Zucman-Rossi J.: SS 11.5
Zurru A.: SS 9.1
